# The Quantitative Biotinylproteomics Studies Reveal a WInd-Related Kinase 1 (**Raf-Like Kinase 36**) Functioning as an Early Signaling Component in Wind-Induced Thigmomorphogenesis and Gravitropism

**DOI:** 10.1016/j.mcpro.2024.100738

**Published:** 2024-02-15

**Authors:** Nan Yang, Jia Ren, Shuaijian Dai, Kai Wang, Manhin Leung, Yinglin Lu, Yuxing An, Al Burlingame, Shouling Xu, Zhiyong Wang, Weichuan Yu, Ning Li

**Affiliations:** 1 Division of Life Science, The Hong Kong University of Science and Technology, Hong Kong SAR, China; 2 Institute of Nanfan and Seed Industry, Guangdong Academy of Sciences, Guangzhou, Guangdong, China; 3 Department of Pharmaceutical Chemistry, University of California San Francisco, San Francisco, California, USA; 4 Department of Plant Biology, Carnegie Institution for Science, Stanford, California, USA; 5 Department of Electronic and Computer Engineering, The Hong Kong University of Science and Technology, Hong Kong SAR, China; 6 Shenzhen Research Institute, The Hong Kong University of Science and Technology, Shenzhen, Guangdong, China

**Keywords:** *in planta* proximity-labeling (IP-PL), 4C quantitative biotinylproteomics, force signaling, biotin occupancy ratio (BOR), WInd-Related Kinase 1 (WIRK 1), rapidly accelerated fibrosarcoma (RAF)-like kinase 36 (RAF36)

## Abstract

Wind is one of the most prevalent environmental forces entraining plants to develop various mechano-responses, collectively called thigmomorphogenesis. Largely unknown is how plants transduce these versatile wind force signals downstream to nuclear events and to the development of thigmomorphogenic phenotype or anemotropic response. To identify molecular components at the early steps of the wind force signaling, two mechanical signaling-related phosphoproteins, identified from our previous phosphoproteomic study of *Arabidopsis* touch response, mitogen-activated protein kinase kinase 1 (MKK1) and 2 (MKK2), were selected for performing *in planta* TurboID (ID)-based quantitative proximity-labeling (PL) proteomics. This quantitative biotinylproteomics was separately performed on *MKK1-ID* and *MKK2-ID* transgenic plants, respectively, using the genetically engineered TurboID biotin ligase expression transgenics as a universal control. This unique PTM proteomics successfully identified 11 and 71 MKK1 and MKK2 putative interactors, respectively. Biotin occupancy ratio (BOR) was found to be an alternative parameter to measure the extent of proximity and specificity between the proximal target proteins and the bait fusion protein. Bioinformatics analysis of these biotinylprotein data also found that TurboID biotin ligase favorably labels the loop region of target proteins. A WInd-Related Kinase 1 (WIRK1), previously known as rapidly accelerated fibrosarcoma (Raf)-like kinase 36 (RAF36), was found to be a putative common interactor for both MKK1 and MKK2 and preferentially interacts with MKK2. Further molecular biology studies of the *Arabidopsis* RAF36 kinase found that it plays a role in wind regulation of the touch-responsive *TCH3* and *CML38* gene expression and the phosphorylation of a touch-regulated PATL3 phosphoprotein. Measurement of leaf morphology and shoot gravitropic response of *wirk1* (*raf36*) mutant revealed that the *WIRK1* gene is involved in both wind-triggered rosette thigmomorphogenesis and gravitropism of *Arabidopsis* stems, suggesting that the WIRK1 (RAF36) protein probably functioning upstream of both MKK1 and MKK2 and that it may serve as the crosstalk point among multiple mechano-signal transduction pathways mediating both wind mechano-response and gravitropism.

## Highlights

• *In planta* labeling of target proteins by MKK1- and MKK2-TurboID fusion proteins.
• The quantitative biotinylproteomics identifies many putative interactors of MKK1/2.
• Biotin occupancy ratio (BOR) measures both specificity and proximity of interactors.
• WIRK1, *i.e.*, RAF36, is a crosstalk mediating both wind- and gravity-force signaling.

## Graphical Abstract

**Figure:**
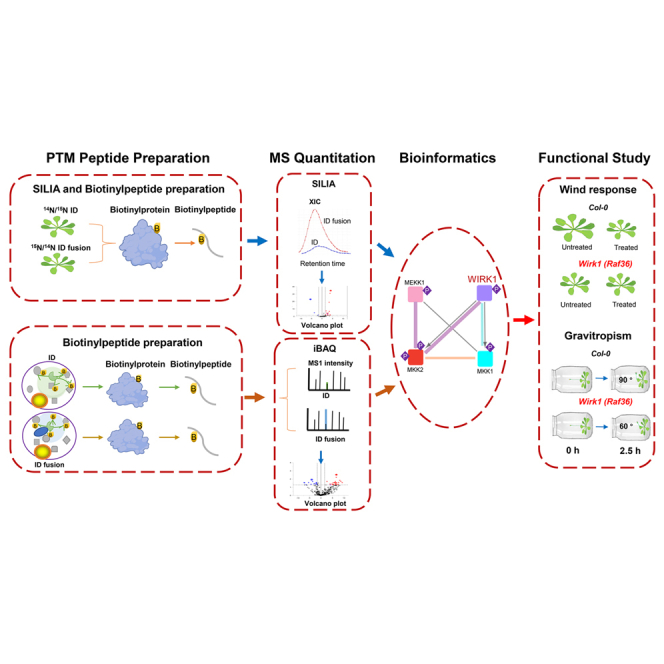

## In Brief

This quantitative biotinylproteomics was separately performed on MKK1-ID and MKK2-ID transgenic plants, respectively, using the genetically engineered TurboID biotin ligase expression transgenics as a universal control. WIRK1 was found to be a common interactor of both MKK1 and MKK2. It was verified to participate in wind regulation of the touch-responsive TCH3 and CML38 gene expression and the phosphorylation of a touch-regulated PATL3 phosphoprotein. This study demonstrated that WIRK1 gene is involved in both wind-triggered rosette thigmomorphogenesis and gravitropism of Arabidopsis stems.

The external natural forces, wind, rain, hail, snow, and touch of objects as well as earth gravitational field, are all able to influence plant growth, development, and morphological architecture. Wind is one of the most prevalent and predominant force stimuli, frequently loaded on terrestrial plants to shape the morphology as well as the physiological and mechanical properties of plants (1, 2, 3, 4). The overall wind response of a plant generally exhibits the change in growth, development, photosynthesis, and reproductivity (2, 5, 6). The salient morphological changes of plants usually include reduced leaf area and shortened stem length as well as, sometimes, decreased shoot weight (7, 8). Given that both wind and flexing produced plants of shorter stem and reduced biomass (9), and the periodic mechanical stimulations of plants generate dwarf plants of delayed flowering phenotypes (10, 11, 12, 13), it is therefore hypothesized that the wind drag force, as one of the most direct effects of wind on the plant response, might share a common signaling network with the touching, brushing, robbing or flexing-induced plant mechano-responses or called thigmomorphogenesis (13, 14, 15, 16). Other than the intermittent, turbulent, and constant (or sporadic) wind-evoked thigmomorphogenesis, plants often exhibit negative anemotropic response, a unique type of thigmotropic response (or force-triggered directional growth), under constant and unidirectional wind application (1, 16), in which plants flex in parallel with the wind direction. Interestingly found in Arabidopsis was that the primary growth region of the inflorescence stem bends windward opposite to the wind direction, exhibiting a positive anemotropism under the constant influence of unidirectional wind (17). This unique positive anemotropism has been speculated to be the consequence of the combinatorial effects of both wind drag force and gravistimulation (17, 18).

To unravel the molecular mechanisms underlying the complex wind force signaling during wind-stimulated thigmomorphogenesis and anemotropism, transcriptomic studies have been conducted on Arabidopsis several times in the past decades (19, 20, 21, 22). Hundreds of those dynamically changing transcripts, resulting from touch and brushing, were found to cover a broad spectrum of molecular and cellular functions, ranging from putative receptors, calcium-binding proteins, phosphatases, kinases, transcription factors, RNA processing enzymes to cell wall biosynthesis-related enzymes (20, 22). These genes are believed to play a similar role in wind force-triggered thigmomorphogenesis and anemotropism in plants.

The induction of the mechano-responsive gene expression and the regulation of plant mechano-response are basically mediated through a network of signaling components (20). The mechano-sensory machineries, including numerous genuine mechano-sensitive channels and multimeric mechano-signalosomes (23), may transduce wind force signals quickly downstream into nuclear events either through both calcium transients [Ca^2+^]_cyt_ (24) and calcium ion-dependent kinases/phosphatases-mediated phosphor-relays or through both receptor-like kinases and mitogen-activated protein kinase (MAPK)-mediated phosphor-relays (21, 25). In fact, many years of molecular biological studies performed on phosphorylation and function of mitogen-activated protein kinase kinase one and 2 (MKK1/2) as well as mitogen-activated *protein kinase kinase kinase 1* (MEKK1) have implicated that the phosphor-relay cascade mediated by both MEKK1 and MKK1/2 might play a role in plant mechano-response (26) and that both MKK1 and MKK2 are components of signaling pathways responsive to a variety of extracellular stimuli, like innate immunity, cold, drought, salt, and reactive oxygen species (ROS) (27, 28, 29, 30, 31). Thus, it has been postulated that protein phosphorylation, as one of the most abundant post-translational modifications (PTMs) in plant cells, may play a critical role in the early stages of mechano-signal transduction (21). Recent phosphoproteomic studies performed on the 40-s touch-stimulated *Arabidopsis* indeed revealed a group of phosphoproteins with a wide spectrum of molecular functions being phosphorylated rapidly upon force loading, among which MKK1/2 module is highly phosphorylated and regulated quickly by seconds of touch, water-sparkling and air-blowing treatments (21). The phosphorylation of a cytoskeletal protein, Touch-Regulated Phosphoprotein 1 (TREPH1), was shown to participate in mechano-signaling during thigmomorphogenesis (21). A pertinent question raised from this study would be: what are the signaling components that might function upstream of MKK1/2 in response to force loadings like the wind drag force?

**Fig. 1:**
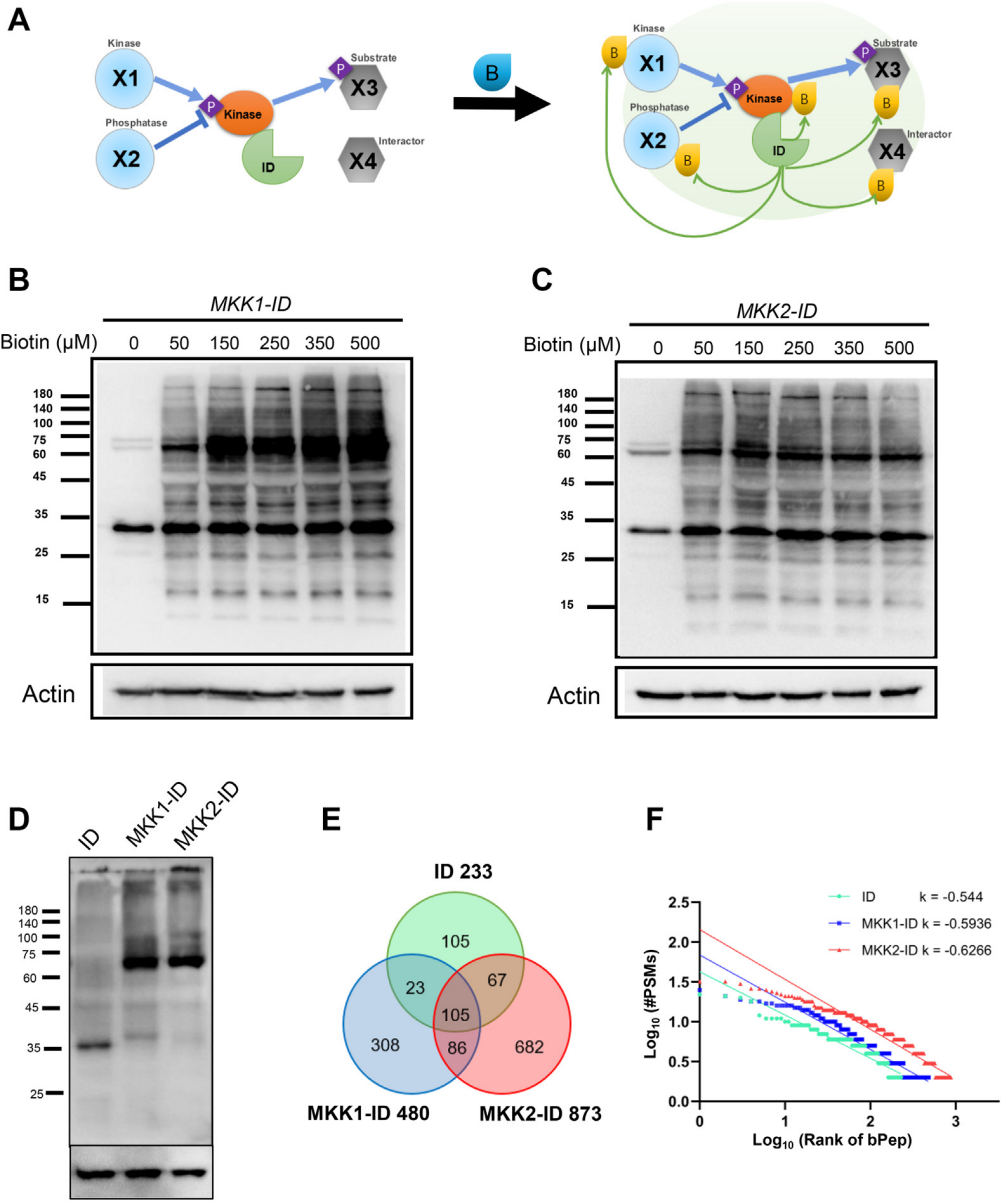
**Mechanism and condition of enzymatic proximity labeling.***A*, schematic representation of proximity-labeling system in kinase cascade. The kinase shown in *orange oval* is a bait protein linked to TurboID, annotated in *green* sector. X1 and X2 in *blue circles* represent kinases and phosphatases, respectively. X3 and X4 in *gray* represent substrate and other interactors, respectively. (*B*) in *yellow drop* represents biotin and P in *purple diamond* stands for the phosphate group. *Blue and green arrows* represent phosphorylation and biotinylation processes, respectively. The *blue line* crossing the stop line represents dephosphorylation by phosphatase. *B* and *C*, PL condition tests of different concentrations of biotin in the medium for MKK1-ID and MKK2-ID transgenics. 50 to 500 μM biotin was added in a medium with 5 μM estradiol for the growth of MKK1-ID and MKK2-ID transgenics. Anti-biotin was used to detect the *in vivo* biotinylation by MKK1-ID and MKK2-ID. Three replicates were completed, and the other two biological replicates were shown in supplemental Fig. S5, *A* and *B*. The biotin addition test was also conducted in ID transgenics, which was shown in supplemental Figure S5*C*. The foundation and verification of MKK1-ID and MKK2-ID transgenic lines are shown in supplemental Figs. S3 and S4. *D*, expression levels of TurboID in three kinds of ID transgenic plants. Anti-TurboID was used to detect the expression level of TurboID or fusion protein in three transgenics. Three biological replicates were conducted and shown in supplemental Figure S6. *E*, a Venn diagram shows the numbers of non-redundant and repeatable biotinylpeptides labeled by ID (233), MKK1-ID (480), and MKK2-ID (873), respectively (supplemental Table S3*B*). The two workflows of this project are shown in supplemental Figs. S1 and S2. The pipeline of data processing was summarized in supplemental Figs. S7 and S8. The total identified PSMs in this experiment are shown in supplemental Fig. S9. *F*, distribution of the PSM numbers of each biotinylated peptide over the rank of the entire population of biotinylpeptides labeled by ID, MKK1-ID, and MKK2-ID, respectively. The k represents the slope of the fitting curve of the rank distribution by Zipf’s law. The fitting linear equations of biotinylpeptides labeled by ID, MKK1-ID and MKK2-ID are y = −0.544x + 1.6285, y = −0.544x + 1.6285 and y = −0.6266x + 2.1588, respectively (supplemental Table S3*B*).

Although conventional interactomic approaches, such as yeast two-hybrid (Y2H) (32, 33, 34), affinity purification with mass spectrometry (AP-MS) (35), and bimolecular fluorescence complementation (BiFC) (36), can be used to discover upstream interactors of MKK1/2, however, in this study, an *in vivo* enzyme labeling-based biotinylproteomics (one of many different types of PTM proteomics), or called proximity labeling (PL) proteomics (37, 38), was chosen to identify putative protein-protein interactions (PPIs). The promiscuously labeling enzymes targeting putative protein interactors, allow the *in vivo* addition of chemical biotin to the neighboring proteins promiscuously within a distance of 10 to 20 nm (37, 38), and enable the capturing of transiently interacting proteins of lower affinity (39, 40). One of these indiscriminately enzymes, APEX2, was genetically engineered from soybean ascorbate peroxidase. It can label proximal proteins within 1 min (41, 42). However, it is toxic to living cells due to the use of H_2_O_2_ (43, 44). BioID, on the other hand, is an R118G mutant of *Escherichia coli* biotin ligase (BirA) (45, 46). Although it is non-toxic to living cells, it still needs approximately 18 h to produce sufficient biotinylation on target proteins (39). Thus, a novel genetically engineered biotin ligase, TurboID (39), was ultimately selected to perform the quantitative PL proteomics on *Arabidopsis* following 5 min of wind treatment. It was believed that this genetically engineered PL enzyme was able to label proximal proteins promiscuously within 5 to 10 min (39, 47). For example, the cell type specifically expressed protein FAMA was fused with TurboID, which was stably transformed into *Arabidopsis* young seedlings to identify cell type-specific and subcellular compartment-specific protein neighborhoods and to establish the putative local interactome (47, 48). Similarly, the biotinylation of neighboring proteins by several types of biotin ligases, BioID, BioID2, TurboID, and miniTurboID, were investigated in *Arabidopsis* cell suspension, *S. lycopersicum* root culture and transient transformed *N. benthamiana* (49). These preliminary studies have established model transgenic plant systems available for performing PL proteomics (49, 50). Recently, the PL proteomics has been applied on a 3-week-old transgenic *Arabidopsis* to identify the cargo spectrum of XPO4 (51). BIN2 protein was fused with TurboID to map its signaling networks in transgenic *Arabidopsis* (52). The successes of these *in vivo* TurboID-labeling experiments prompted us to integrate the previously established 4C quantitative PTM proteomics (53) with the TurboID-based *in planta* proximity labeling (PL) to develop a 4C *in planta* quantitative biotinylproteomics to identify the putative interactors, either upstream or downstream substrates, of MKK1 and MKK2 from the transgenic *Arabidopsis* plants expressing MKK1-ID and MKK2-ID fusion proteins (Fig. 1*A*). To that end, we first optimized a tandem enrichment protocol consisting of both biotinylprotein and biotinylpeptide isolation steps to reduce the chance of getting false positives. Secondly, biotin was supplemented to the growth medium to enhance the TurboID-mediated biotin-labeling of target proteins. Thirdly, we applied both the intensity-Based Absolute Quantification (iBAQ) quantitation (54) and the stable isotope labeling in *Arabidopsis* (SILIA)-based quantitation (55) on biotinylproteins of those transgenic plants. As a result, the improved *in planta* quantitative biotinylproteomics allowed us to identify the WInd-Related Kinase 1 (WIRK1), a previously classified *Arabidopsis* Group C Raf-like kinase 36 or called RAF 36, as a putative interactor of both MKK1 and MKK2. Finally, bioinformatic analysis and molecular biological experiments confirmed and validated that WIRK1 might function upstream of MKK1/2 and play a role in the regulation of wind force response and gravitropism. A wind mechano-signaling model, consisting of the core signaling-related kinases, WIRK1, MEKK1, MKK1, and MKK2, is consequently proposed for the wind force mechano-response and gravitropism of *Arabidopsis* aerial organs.

## Experimental Procedures

### Experimental Design and Statistical Rationale

*In planta* TurboID-based proximity labeling (PL) or *in vivo* biotin ligase-catalyzed biotin-labeling of neighboring proteins, was combined, respectively, with the intensity-Based Absolute Quantification proteomics (iBAQ; supplemental Fig. S1) (54) and SILIA-based quantitative biotinylproteomics (namely SILIA-based PTM proteomics; supplemental Fig. S2) (55, 56, 57). The iBAQ-based quantitative biotinylproteomics and the SILIA-based quantitative biotinylproteomics were defined as Experiment 1 (E1) and Experiment 2 (E2), respectively. To identify putative interactors of MKK1 and MKK2, three transgenic plants, named as *ID*, *MKK1-ID* and *MKK2-ID*, were investigated in E1 while *MKK1-ID* and *MKK2-ID* transgenic plants were studied in E2. Both experimental workflows of quantitative biotinylproteomics are composed of three steps: first C, *in vivo* **C**hemical labeling (^15^N and ^14^N) of the total cellular proteins of transgenic plants; second C, **C**hromatographic enrichment and LC-MS/MS analysis of the prepared biotinylpeptides; third C, **C**omputational analysis and bioinformatic analysis of MS data. Finally, data of both iBAQ- and SILIA-based quantitative biotinylproteomics were integrated bioinformatically to generate a model signaling network for wind drag force. A candidate interactor of choice for both MKK1 and MKK2 was eventually chosen to conduct the **C**onfirmation (fourth C) and the validation experiments using molecular biology and genetics. In total, three biological replicates (Rep1, Rep2, Rep3) were performed in each of E1 and E2 experiments. In E1, MS1 precursor ion intensities of both ^15^N- and ^14^N -coded biotinylpeptides were measured by LC-MS/MS from ID, MKK1-ID and MKK2-ID peptide samples. The six Mascot-searched MS data sets for both the heavy (m1, m2 and m3) and the light (m4, m5 and m6) nitrogen-labeled biotinylpeptides pairs were summarized in supplemental Table S1. The quantifiable biotinylproteins of both MKK1-ID/ID sample and MKK2-ID/ID sample were calculated using MS1 precursor ion intensities presented in supplemental Table S4. These Log2-ratios of MS1 precursor ion intensities of MKK1-ID/ID and MKK2-ID/ID were generated for the iBAQ quantitation. However, in E2, XIC intensities of both ^15^N and ^14^N-coded biotinylpeptides were measured by LC-MS/MS for MKK1-ID and MKK2-ID protein samples. The log2-ratios of XIC intensities of MKK2-ID/MKK1-ID biotinylproteins were generated using the previously published SILIA-based SQUA quantitation (supplemental Table S5) (55, 57) in order to identify the putative MKK1 and MKK2 interactors.

In both iBAQ- and SQUA-based quantitation, both ^15^N and ^14^N-coded biotinylpeptide identification was achieved using the Mascot search engine with a false discovery rate (FDR) of 1% (57) while the log2-ratios of biotinylproteins were firstly adjusted either based on three-way analysis of variance (ANOVA) or the empirical Bayes method depending on the batch effects (55). The statistical selection of putative MKK1 and MKK2 interactors from biotinylproteins was performed on the log2-ratios of biotinylproteins ion intensities (MS1 precursor ion intensity or XIC data) using a one-sample *t* test, the *p*-values of *t* test on log2-ratios of UPAs (Unique Peptide Arrays) of biotinylproteins were analyzed using the multiple hypothesis testing corrections by Benjamini and Hochberg (BH) procedure (58). The threshold was 5% false discovery rate (BH-FDR). The biotinylproteins with a log-ratio mean absolute value outside 1.58 (|fold change| ≥ 3) and BH-FDR value ≤ 0.05, were selected as the significant candidates, or putative interactors of MKK1 or MKK2.

### Plant Materials and Transgenic *Arabidopsis*

*Arabidopsis thaliana* ecotype *Columbia-0* (*Col-0*) and T-DNA insertion lines, SALK_015914C (insertion in AT4G26070, *mkk1*), CS1002950 (insertion in AT4G29810, *mkk2*), SALK_044426 (insertion in AT5G58950, *wirk1-1*, or called *raf36-1*), GK-459C10 (insertion in AT5G58950, *wirk1-2*, or called *raf36-2*), *acs octuple* mutant (CS16651, an ACC biosynthesis-deficient mutant) and SALK_093994 (insertion in AT1G72160, *patl3*), were purchased from *Arabidopsis* Biological Resource Center] (ABRC).

The plasmid encoding the TurboID gene was a gift from Dr Alice TING’s laboratory at Stanford University. The binary pER10 vectors harboring the recombinant gene cassettes (supplemental Fig. S3), Pro_ER_-UTRb-MKK1-TurboID-His_x8_ (MKK1-ID), Pro_ER_-UTRb-MKK2-TurboID-His_x8_ (MKK2-ID), Pro_ER_-UTRb-TurboID-His_x8_ (ID) were generated by ligation of a linearized pER10 plasmid having an XhoI and PacI site at 5′ and 3′ end, respectively, with the sticky ends generated by both XhoI and AscI restriction endonucleases on UTRb DNA fragment, as well as by both AscI and AvrII enzymes on DNA fragments of MKK1 or MKK2 genes and by both AvrII and PacI enzymes on the DNA fragment of TurboID gene. The full length of UTR of Rubisco small chain 1B (AT5G38430) was amplified from *Arabidopsis* genomic DNA using the following primers (Underlined restriction sites and 8× His):

Rbc1B-F: 5′-GTTTCTCGAGGATAAGGGTGTCAACACCTTTCC-3′;

Rbc1B-R: 5′-TTTGGCGCGCCTACTTCTTCTTCTTCTTCTTTTG-3′.

The 1.8-kb DNA fragments encoding the full-length MKK1 and MKK2 genes and the 0.9-kb DNA fragment encoding TurboID (39) were amplified by polymerase chain reaction (PCR) using the following primers, separately.

MKK1-F: 5′-TTTGGCGCGCCATGAACAGAGGAAGCTTATGC-3′

MKK1-R: 5′-TTTCCTAGGGTTAGCAAGTGGGGGAATCAAAG-3′

MKK2-F: 5′-TTTGGCGCGCCATGAAGAAAGGTGGATTCAGC-3′

MKK2-R: 5′-TTTCCTAGGCACGGAGAACGTACCAGACAG-3′

Turbo-F: 5′ TTTCCTAGGAAAGACAATACTGTGCCTCTGAAG-3′

Turbo-R:5′-CCCTTAATTAATCAAT*GATGATGGTGGTGATGATGAT*GCTTTTCGGCAGACCGCAGACTG-3′.

All primers used in this manuscript are summarized in supplemental Table S10 and are not mentioned again.

### Plant Growth and Medium Preparation

Transgenic *Arabidopsis* seeds (100 μl, about 1000 seeds each genotype) of *ID*, *MKK1-ID*, or *MKK2-ID* genotypes were sterilized with 70% ethanol, 30% bleach in 0.1% Triton X-100 sequentially, and vernalized at 4 °C for 4 to 7 days in darkness. These seeds were sown and grown on either ^14^N- or ^15^N-coded solid agar medium supplemented with 150 μM biotin and 5 μM ꞵ-estradiol in transparent glass jars of 7.7-cm diameter and 12.7-cm height. The process of stable isotope chemical labeling of *Arabidopsis* is called SILIA (59). The growth medium contains 18.8 mM KNO_3_, 5 mM NH_4_NO_3_, 100 μM H_3_BO_3_, 3 mM CaCl_2_·2H_2_O, 0.1 μM CoCl_2_·6H_2_O, 0.1 μM CuSO_4_·5H_2_O,100 μM FeSO_4_·7H_2_O, 1.25 mM KH_2_PO_4_, 5 μM KI, 1.5 mM MgSO_4_·7H_2_O, 100 μM MnSO_4_·H_2_O，100 μM Na_2_EDTA·2H_2_O, 1 μM Na_2_MoO_4_·2H_2_O, 30 μM ZnSO_4_·7H_2_O, 10 g/L sucrose, 1 mg/L thiamine HCl, 0.1 mg/L pyridoxine, 0.1 mg/L nicotinic acid and 100 mg/L myo-inositol. The pH of the medium was adjusted to 5.7 with 2 M KOH and then 0.8% bacteriological agar was added followed by both high-pressure and high-temperature sterilization (59). Into each of the sterilized jars, 45 ml of autoclaved medium were poured where 20 seeds were sown on the surface of solid agar. The Hydrophobic Fluoropore Membrane (HFM, http://www.shjiafeng.com, Jiafeng) was employed to cover the jar to keep it permeable to air and sterile. Plants were grown under a constant light of light intensity of 140 to 220 μE m^−2^ s^−1^, measured by an IL 1700 research radiometer (International Light). The temperature in the growth room was at 23 °C ± 1 deg. C. Humidity was 35 to 45%.

### Wind Treatment and Tissue Harvest

Wind force loading was achieved by generating transient and turbulent air currents through a fan (BP26 Gaiatop). Before applying wind onto plants grown in glass jars, all membrane covers were first removed to keep the humidity and the pressure of jars equal to those of the growth room. The wind speeds of 1 to 5 m/sec range were measured by an anemometer for current speed and pressure measuring instruments (KXYL-600B, Kaixiang Instrument Co, Ltd). The wind speed at the surface of plants was controlled by adjusting the distance between the fan and the plants as well as the resolution of the fan. The wind of 4 m/sec (10 Pa) was set as experimental wind for treating transgenic plants (Video Clip 1). The wind drag force (U) applied to each plant was calculated as 1 to 2 mN (24). This type of wind was defined to be a gentle breeze according to the Beauford scale. Aerial parts of plants were quickly frozen in liquid nitrogen right after the wind treatment and stored at −80 °C for later analysis. All transgenic plants of *ID*, *MKK1-ID*, and *MKK2-ID* genotypes were treated at the same time and collected simultaneously with liquid nitrogen for later protein sample preparation.

### Experimental Plant Groups and the Total Cellular Protein Extraction

In experiment 1 (E1), three groups of transgenic plants, *ID, MKK1-ID*, and *MKK2-ID*, were grown separately on ^15^N- or ^14^N-coded growth medium for three consecutive times to produce three biological replicates of plant tissues for each genotype of transgenic plants, whereas in experiment 2 (E2), *MKK1-ID* and *MKK2-ID* plants were grown separately in ^15^N- or ^14^N-coded growth medium again for three consecutive times to generate three biological replicates of plant tissues for each genotype of plants. From each biological replicate, 12 to 28 g of plant aerial tissues were harvested from 40 to 60 glass jars. The mixing strategy of isotope-coded plant tissues followed the 4C quantitative PTM proteomics protocol published previously (21, 57, 59).

The total cellular proteins were extracted under the fully denaturing condition where the frozen tissues were thawed inside of urea extraction buffer (UEB) (59). The UEB buffer contained 8 M Urea, 150 mM Tris (pH 7.6), 0.8% sodium dodecyl sulfate (SDS), 1.2% Triton X-100, 20 mM ethylenediamine tetraacetic acid (EDTA), 20 mM ethylene glycol-bis(β-aminoethyl ether)-*N,N,N′,N′*-tetraacetic acid (EGTA), 50 mM NaF, 1% glycerol 2-phosphotase disodium hydrate, 5 mM dithiothreitol (DTT), 1 mM phenylmethylsulfonyl fluoride (PMSF), 0.5% phosphotase cocktail 2, Complete EDTA free protease inhibitors cocktail (3 tablets in 200 ml of buffer), 5 mM ascorbic acid and 2% polyvinyl polypyrrolidone (PVPP). The fine frozen tissue powders were gradually dripped into the swirling UEB buffer to reach a ratio of 1: 4 (w/v) and stirred for 3 min. Plant cell debris was removed using centrifugation at a speed of 10,000*g* for 20 min at 12 °C. The supernatant containing the total cellular proteins was precipitated with a pre-cooled solution of acetone to methanol at 12: 1 (v/v) at −20 °C overnight. The precipitated proteins were collected by centrifugation of the same speed for 20 min, and the residue pigment and urea precipitates were rinsed with 20 volumes (solution/protein, v/w) of pre-cooled mixed solution (acetone/methanol/H_2_O at a ratio of 12:1:1.4, v/v/v). The air-dried protein pellets were resuspended with Biotinylated Protein Enrichment Buffer (BPRB) consisting of 8 M urea, 200 mM NaCl, 0.5% SDS, 10 mM sodium phosphate (pH 7.4), 100 mM Tris (pH 7.4) (60, 61). The protein amount of each biological repeat was measured to be 0.22 to 0.45 g. The protein extraction yield was 1 to 2%. The concentration of protein was quantified by a calculation based on bovine serum albumin (BSA) standard curve made using DC protein assay (Bio-Rad DC Protein Assay Kit).

### Enrichment of Biotinylproteins

A milliliter (mL) of streptavidin beads (Pierce High Capacity Streptavidin Agarose, Thermo Scientific Inc, USA) were equilibrated with 5 ml of BPRB for three times. The equilibrated beads were mixed with the 0.22 to 0.45 g of total cellular proteins and incubated at room temperature for at least 6 h and washed three times using Biotinylated Protein Washing Buffer 1 (BPWB1), including 8 M urea, 0.2 M NaCl, 0.5% SDS, 100 mM Tris (pH 7.4), three times with BPWB1 plus 2% SDS, and 8 to 10 times with one × PBS. In each step of washing, the streptavidin beads were collected by centrifugation at 1000*g* for 2 min. Biotinylproteins were eluted by an elution buffer containing 2% SDS, 3 mM biotin, and 15 mM Tris (pH 6.8) by rotating the solution at room temperature for 15 min, followed by another 15 min of heating at 96 °C. After 1 min of centrifugation at 1000*g*, the supernatant containing biotinylproteins was collected and vacuum-dried by Speed Vac (SPD1030, Thermo Scientific Inc) to be concentrated, which was consequently supplemented with 30% glycerol and 1% β-mercaptoethanol, leading to a final concentration of biotinylproteins to one to 2 μg/μl.

### In-gel Digestion and Peptides Harvest

The biotinylproteins were run on SDS-PAGE, a 6 × 9-cm slab gel, to remove SDS and extra biotin, which was followed by an in-gel trypsin digestion according to a previously published procedure (62). Usually, 0.3 to 0.5 mg of biotinylproteins were fractionated on SDS-PAGE. After running at 120 V for 1 h, the protein gel was stained by Coomassie Brilliant Blue and excised into 1 × 1 × 1 (mm) cubic pieces. Biotinylproteins were digested by trypsin protease (Promega) at ratio of 20: 1 (w/w) at 37 °C. The protein samples were rotated overnight followed by an addition of extra trypsin (protein: trypsin is 40: 1, w/w) and digestion for another 6 h. The peptides from biotinylprotein samples were harvested by sonification in a solution containing 50% acetonitrile and 1% trifluoracetic acid in ice water. After acetonitrile was removed by volatilization in Speed Vac, the peptides were desalted and enriched by an HLB cartridge (Oasis HLB, WAT094225, Waters).

### Enrichment of Biotinylpeptides

The peptides of 100 to 300 μg prepared from the biotinylprotein samples were dissolved in 50 mM HEPES (pH7.5) and incubated with 50 μl equilibrated streptavidin beads for 2 h at room temperature under constant rotation. Following 2 min of centrifugation at 1000*g*, the supernatant was transferred to another Eppendorf tube, and the beads were washed at least five times with a solution of 50 mM HEPES. Biotinylpeptides were eluted twice off beads at room temperature for 1 h using an elution buffer containing 0.5% formic acid and 70% acetonitrile (63, 64). The eluent was vacuum-dried by Speed Vac and desalted by ZipTip (Merk Millipore) for subsequent MS analysis.

### LC-MS/MS Analysis

Biotinylpeptides were dissolved in solvent A (0.1% formic acid), 0.3 to 0.5 μg of which were used in a run-through LC-MS/MS. The LC-MS/MS analysis of biotinylpeptides was performed on an EASYSPRAY HPLC C18 column (3 μm, 100 Å, 75 μm × 150 mm) (Thermo Fisher Scientific, Odense, Denmark) coupled with an Orbitrap Fusion Lumos Tribrid Mass Spectrometer (Thermo Fisher Scientific). This system used to separate the peptides was at a constant flow rate of 300 nl/min on a gradient of 0 to 10 min 2% solvent B, 10 to 13 min 2 to 5% solvent B, 13 to 85 min 5 to 30% solvent B, 85 to 87 min 30 to 50% solvent B, 87 to 89 min 50 - 5% solvent B, 89 to 94 min 5% solvent B, 94 to 95 min 5 - 2% solvent B where solvent B is 0.1% formic acid in acetonitrile. Mass resolution was set at 120,000 for intact peptides (MS1) and at 30,000 for ion fragments (MS2) with a m/z range of 375 to 1500 under normalized collision energy (NCE) of 30 activated by Higher-energy collisional dissociation (HCD). The data-dependent acquisition (DDA) mode was adopted for the intensity threshold which exceeded the ion count of 2E^4^, and the exclusion duration was 30 s. A filtered charge state of two to seven for a maximum injection time of 50 ms, an automatic gain control (AGC) target value of 4E^5^ charges, and the AGC target value of MS/MS is 5E^4^ whose maximum injection time is 100 ms. An isolation Window of 1.6 m/z was employed.

### Peptide Searching and SILIA-Based Biotinylprotein Quantitation

The MS raw data obtained from a mass spectrometer was converted into Mascot generic format (mgf) and mzXML by MSCovert (65) (vertion: 3.0.20236 64 bit). The mgf files were searched and matched MS2 spectra on Mascot (65) (version: version 2.7.0, 64 bit, Matrix Science) based on TAIR10 (35,387 proteins, https://www.arabidopsis.org/download_files/Sequences/TAIR10_blastsets/TAIR10_pep_20101214_updated) database. The false discovery rate (FDR) was estimated by the target-decoy strategy as previously described (57, 66). In this experiment, protein digestion was carried on by trypsin and set with a maximum of missed cleavage as 2. When MS2 spectra matched with the database on Mascot, the setting was as follows: The mass tolerance was ±10 ppm for MS1 and 0.02 Da for MS2. Carbamidomethyl (57.021 Da) on Cystine was set as a fixed modification. The variable modifications for biotin peptide-searching were oxidation (15.995 Da) on Methionine, biotinylation (226.078 Da) and sulfoxide biotinylation (242.073 Da) either at N-terminus or lysine(K) of peptides (67, 68). The quantitation method “^15^N metabolic labeling” was specified. Mascot Percolator (Version 3.1) was appended to estimate the FDR. The peptide-spectrum match (PSM) cut-off threshold was set at 1%. The data files generated from Mascot were consequently subjected to SQUA-N software (Stable isotope labeling-based QUAntitation-Heavy Nitrogen labeling, Version 1.0) (55, 69) for further protein quantification. The quantification of peptides and biotinylated peptides was consequently converted into protein quantification as previously described (55). During the SQUA software analysis, the criteria for the selection of quantifiable peptides were the followings: 1. the PSM number of ^15^N-labeled PTM peptides ≥1; 2. the PSM number of ^14^N-labeled PTM peptides ≥1; 3. the number of different biological replicates ≥1 for each peptide; 4. the number of different biological replicates ≥3 for each protein; 5. the number of PSMs identified from either forward or reciprocal experiment divided by the total number of PSMs ≥0.2; 6. the Mascot delta score ≥10 for protein quantification; 7. the MS1 tolerance ≤0.05 Da. The batch effect adjustment was applied to the quantification to eliminate the variances from tissue harvesting and mixing, protein extraction, protein digestion, and peptide enrichment in different biological repeats (55, 69, 70). In E1, all six sets of Mascot-searched results from three biological replicates of ID, MKK1-ID, MKK2-ID samples have been deposited into ProteomExchange repository, in which there were three sets of Mascot-searched heavy peptides (labeled as m1, m2, and m3) and three sets of light peptides (labeled as m4, m5, and m6), which (supplemental Table S1) were used for iBAQ quantitation. In E2, the Mascot-searched results from six experimental replicates, containing both forward (F1, F2, F3) and reciprocal (R1, R2, R3) mixing data generated from three biological replicates of samples (supplemental Table S2), were labeled as n1, n2, n3, n4, n5 and n6 as our previously published papers (21).

### iBAQ-Based Biotinylprotein Quantitation

The MS1 precursor ion intensities of peptides were first retrieved by in-house built software SQUA from the Mascot-searched data. The peptide ion intensity data from three biological replicates (comprising six experimental replicates, m1, m2, m3, m4, m5, and m6) of transgenic plants (ID, MKK1-ID, MKK2-ID) were subsequently compiled in supplemental Table S1. The quantification was performed using the standard intensity Based Absolute Quantitation (iBAQ) method (54). First, the MS1 precursor ion intensity values of biotinylpeptides were extracted from each fraction. A half value of the minimum intensity was used to replace the zero intensity of all extracted intensities found in each fraction (55). The number of PSM of each quantifiable biotinylpeptide is ≥2. All peptides from biotinylproteins were combined for biotinylprotein quantitation. Only the biotinylproteins of ≥3 biological replicates were used for further quantification. For each quantifiable biotinylprotein, the calculation of iBAQ value of biotinylprotein was normalized by the total MS1 precursor ion intensity of peptides on a run and the amount of transgenic protein (54, 71). The log2-ratios of the biotinylproteins in each replicate were calculated by dividing the two iBAQ values of two isotope-coded biotinylprotein samples. All log2-ratios were subsequently analyzed by Grubb’s test to remove outliers, followed by one-sample *t* test and multiple hypothesis testing corrections (72, 73). Eventually, all the quantifiable biotinylproteins resulted in a mean value of corresponding log2-ratios and a *q*-value calculated according to Benjamini-Hochberg procedure (58). The significantly selected putative interactors of the bait protein had a *q*-value ≤0.05 and an absolute value of log-ratio ≥1.58 (|fold change| ≥ 3).

### Bioinformatics Analysis

Functional analysis, including Gene Ontology Analysis and protein domain analysis, was performed using DAVID Bioinformatics Resources (74, 75), where InterPro method was applied in protein domain analysis (76). The figures of Gene Ontology Analysis including biological process enrichment, cellular component enrichment, and molecular function enrichment were made using the R with ggplot2 packages.

The primary sequences of biotinylproteins were downloaded from Uniprot (https://www.uniprot.org/). ICM-BrowserPro 3.9 software was used for the visualization of 3D structures of biotinylproteins and the positions of those biotinylated lysine residues.

*Biotin occupancy* (BO_n_) of a biotinylprotein (denoted as n) is equal to the ratio of the number of biotinylated lysine (k_biot_) to the percentage of lysine number in the total polypeptide length (K%) of this biotinylprotein. The equation is: BO_n_ = k_biot_/(K%). *Biotin occupancy ratio* (BOR) of a biotinylprotein n is calculated by dividing the BO_n_ value of a biotinylprotein n identified from either *MKK1-ID* or *MKK2-ID* plants by the BO_n_ value of the same putative interactor identified from *ID* transgenic plants. If the biotinylprotein n is missing from MS data of the transgenic plant ID, the zero is normally substituted with a small number according to the published approach (21, 57, 68).

### Generation of Module and Volvox Plot

MKK1 and MKK2 putative interactors (or significantly selected biotinylproteins based on iBAQ quantification) were submitted to STRING (77) (https://string-db.org/) database analysis with a confidence score ≥0.7 and BioGrid (78) database analysis to retrieve the protein-protein interaction data of MKK1 and MKK2 putative interactors. In combination with results from the TurboID-based proximity labeling and quantitative biotinylproteomics, the degree of protein interactions (DPIs) and edges (protein-protein interaction, PPI) of significantly selected interactors were calculated. iTAK (http://itak.feilab.net/cgi-bin/itak/index.cgi) was used to predict the functions of kinases and transcription factors of these MKK1- and MKK2-interacting proteins (79). According to the information, a group of selected putative interactors of MKK1 and MKK2 kinases that have both DPI and PPI values were subjected to MONET (80) modularization analysis. Both PPI module and the Volvox network of interactors were visualized using Cytoscape (81). To establish putative interactions between kinases and substrates among all interactors of MKK1 and MKK2, we used GPS 5.0 to predict the possible kinase family for the specific substrates (82, 83), and the phosphosites information were obtained from both the identified phosphopeptides of MKK1 and MKK2 biotinylproteins and reanalyzed touch induced phosphoproteomics data by SQUA-N (21).

### Western Blotting

The tissues were collected in liquid nitrogen, ground into fine powders, and extracted with UEB buffer as above mentioned. Proteins of 50 μg were loaded onto either 10% or 12% SDS-PAGE gel. Fractionated proteins were transferred to PVDF membranes (GE Healthcare). The membrane was incubated with both primary and secondary antibodies. Immobilon forte western hrp substrate (WBLUF0500, Millipore) was used for membrane exposure and signal detection. The secondary antibodies were anti-biotin-HRP polyclonal antibodies (7075, Cell Signaling Technology, 1:2000), while the useful primary antibodies were the anti-TurboID polyclonal antibodies (AS20 4440, Agrisera, Sweden, 1:2000), anti-actin monoclonal antibody (a0480, Sigma Aldrich,1:5000) and anti-*p*PATL3 polyclonal antibodies (GL Biochem, 1:1000) targeted to the sequence of MIPQNLG*p*SFKEESSC.

### RT-qPCR

RNA sample preparation for RT-qPCR was carried out based on the standard protocols. The total cellular RNA was isolated through TRIzol (Thermo Fisher Scientific) extraction method and incubated with DNase I (New England Biolabs) to remove DNA contamination. The total RNA was reversely transcribed into cDNA using SuperScript III First Strand Kit (Invitrogen, Thermo Fisher Scientific). The quantitative PCR was performed on a LightCycler 480 instrument II (Roche) through LightCycler 480 SYBR Green I Master (Roche) based on a 20 μl reaction volume system containing 6.6 μl PCR-grade water, 10 μM Primer, and 10 μl SYBR Green with a 384-well plate according to the manufacturer’s specifications. The PCR program contained pre-incubation at 95 °C for 10 min, 45 cycles of denaturation at 95 °C for 10 s and annealing at 60 °C for 18 s, and last extension at 72 °C for 15 s. The melting curve and cooling steps were carried out according to the manufacturer’s protocol. All the primer sequences were listed in the supplemental Table S10. According to a previous study, UBQ10 (AT4G05320) considered to be a housekeeping gene was selected as an internal control (84). The cycle number at the threshold was applied for gene expression quantification since the previous 2^−ΔΔ^Ct method (85).

### Gravitropic Response

*Arabidopsis* plant bolted with 2 to 5 cm long inflorescence was selected for the negative gravitropic response assay. Plants of different genotypes were placed horizontally in a dark tent. Photographs were taken from the horizontal side every 15 min until the end of 150 min according to the previously published method (70, 86). The angle of gravicurvature was measured using ImageJ prior to comparison and plotting (version 1.52i, National Institutes of Health).

To assay for the negative gravitropism of mutants’ etiolated seedlings, the sterilized and vernalized *Arabidopsis* seeds were sown in a square Petri dish (120 mm × 120 mm), and three rows were sown in a single Petri dish. After plates were placed vertically under constant light (70 μmol m^−2^ s^−1^) to induce the seed germination for 24 h, they were placed back to a light-proof tent for 3 days (87). The etiolated seedlings were turned 90° while the seedlings’ rows were vertical to the surface of the earth. After 12 h of gravistimulation, the photographs were taken under the dim green light (88), and the angles of seedlings were measured by ImageJ to continue the analysis.

## Results

### Construction of *ID*, *MKK1-ID*, and *MKK2-ID* Transgenic Plants and *In planta* Biotin-Labeling

A chemical estradiol-inducible promoter was chosen to control the expression of transgenes, TurboID (denoted as ID) biotin ligase or its fusion proteins, MKK1-ID and MKK2-ID, in *Arabidopsis*. The binary vector pER10 of the estradiol-inducible promoter that drives the *MKK1-ID* and *MKK2-ID* transcript production (89, 90, 91) was transformed separately into *mkk1* or *mkk2* mutant backgrounds (supplemental Fig. S3). T3 homozygous transgenic lines of both *MKK1-ID* and *MKK2-ID* genotypes were collected, confirmed using PCR and DNA sequencing (supplemental Fig. S3) and subjected to subsequent quantitative biotinylproteomic experiments. The morphology of these transgenic plants was compared with those of *Col-0*, *mkk1* and *mkk2* mutant plants (supplemental Fig. S4, *A–C*). It was found that the morphologies of both *MKK1-ID* and *MKK2-ID* transgenic plants were indistinguishable from those of *Col-0* plants. Both MKK1-ID and MKK2-ID fusion proteins were successfully expressed and clearly biotinylated as compared to that of wild-type, *mkk1* and *mkk2* mutant plants (supplemental Fig. S4*D*). Because both *mkk1* and *mkk2* mutants have been shown to exhibit a certain level of resistance during mechano-response (21, 92), we therefore examined whether or not these two fusion kinase transgenic plants were able to restore the normal mechano-response of transgenic *Arabidopsis*. Thus, we applied touch treatment on both *MKK1-ID* and *MKK2-ID* transgenic plants and *Col-0*. As expected, the touched *MKK1-ID* and *MKK2-ID* transgenic plants exhibited a delayed bolting phenotype as did *Col-0* (supplemental Fig. S4, *E–G*). Taken together, these preliminary experiments suggested that these transgenic fusion biotin ligases are neither inactive in labeling cellular proteins nor substantially interfering with the normal mechano-response. The transgenic plant expressing *ID* gene was constructed under the same chemical-inducible promoter, and the genetically engineered biotin ligase transgene was transformed into the wild type (*Col-0*) to serve as a control during the quantitative PL proteomics.

The biotinylation of cellular proteins, such as histones, in all three transgenic plant cells are supposed to be catalyzed by both plant endogenous enzymes and foreign bacterium biotin ligase (93). The external supplementing of biotin molecule enhances the biotin homeostasis and promotes biotin-labeling of cellular proteins (52). Subsequently, we measured the effect of externally supplemented biotin on the biotin-labeling of cellular proteins in all three transgenic plants (Fig. 1, *B* and *C* and supplemental Fig. S5). The concentrations of testing biotin supplement varied 10-fold within a range of 50 to 500 μM. It was found that 150 μM biotin supplement allowed all three transgenic plants to have the highest biotin-labeling capability. Most importantly, this level of biotin supplement was non-toxic to the growth of transgenic *Arabidopsis*.

Because the 5 μM of estradiol has been shown to be sufficient to induce the transgene expression in plants (94, 95), all three transgenic plants used for quantitative biotinylproteomics were subjected to the same 5 μM of estradiol induction (Fig. 1*D* and supplemental Fig. S6) in the presence of optimal 150 μM of biotin supplement. As the previous experiments have tested with a wind speed of 1 to 5 m/sec during the wind mechano-response study (7, 24, 56, 96), the wind force stimulus of our choice was 4 m/sec. To reduce the interference of mechano-responsive transcripts and newly synthesized proteins on profiling of the putative kinase interactors, we also applied the wind force loading only for 5 min on all transgenic plants. It was believed that this relatively short period of force loading would help enhance the identification of putative interactors of MKK1 and MKK2 present at the initial time of wind response rather than the latently synthesized protein modifiers of wind force signaling as the transcription of both wound-responsive and touch-responsive begin after 3 to 5 min of treatment (97).

### Computational and Bioinformatic Analysis of Biotinylproteomic Data

In this study, two sets of biotinylproteomic data were generated for both E1 (supplemental Fig. S1 and supplemental Table S1) and E2 (supplemental Fig. S2 and supplemental Table S2) experiments. The workflows for computational analysis of MS/MS data as well as iBAQ- and SILIA-based PTM peptide quantification are summarized in supplemental Figs. S7 and S8, respectively. As a result, E1 and E2 biotinylproteomics allowed us to identify 680, 1428 and 1543 non-repeatable (PSM ≥1) biotinylpeptides, of which 233, 480 and 873 biotinylpeptides were repeatable (PSM >1), respectively (Fig. 1*E*, supplemental Fig. S9, *A*–*C* and supplemental Tables S1, S2, S3, *A* and *B*). The relationship between the PSM discovery frequency of each biotinylated peptide and the ranking of the biotinylated peptides can be described by Zipf’s law, with slopes of −0.4373, −0.436 and −0.3885 for ID, MKK1-ID and MKK2-ID, respectively (Fig. 1*F* and supplemental Table S3*B*). The slope rate suggests the combinatorial state of the degree of protein biotinylation and the coverage of biotinylproteomics. Under the same biotinylproteomic protocol, the smaller slope rate stands for a lower PTM protein coverage of biotinylproteomics. Analysis of peptide length distribution of biotinylpeptides over the ranking of detected biotinylpeptide showed that 86.2%, 86.4% and 82.4% of the biotinylpeptides had sequence lengths ranging from 7 to 25 amino acids for ID, MKK1-ID and MKK2-ID, respectively (supplemental Fig. S9, *D*–*F* and Supplemental Table S3*B*).

**Fig. 2:**
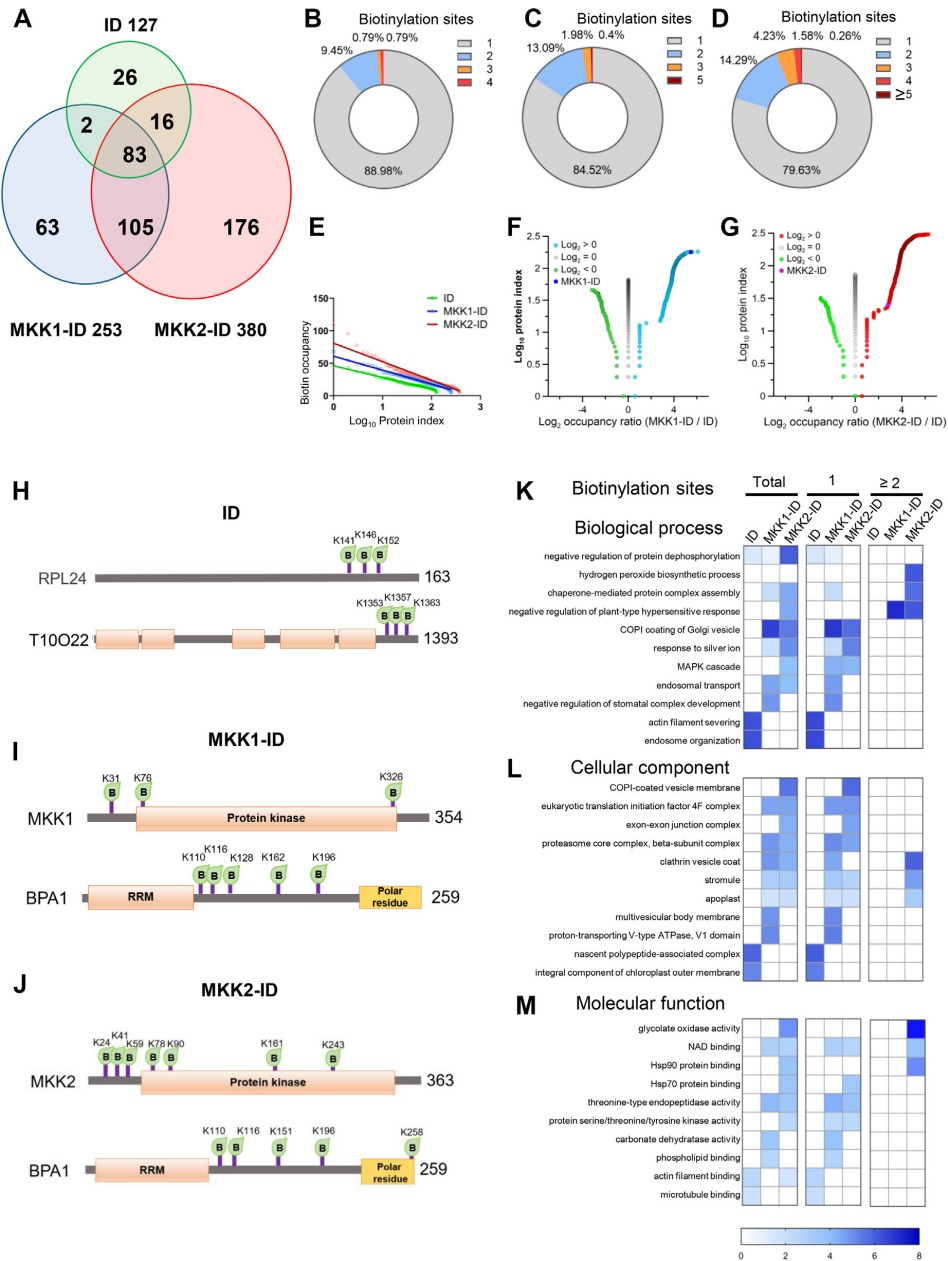
**Proteomic analysis of biotinylproteins in transgenics.***A*, a Venn diagram shows the numbers of biotinylproteins identified in *ID*, *MKK1-ID* and *MKK2-ID* transgenics. A total of 127, 253 and 380 biotinylproteins were identified in *ID* (*green*), *MKK1-ID* (*blue*) and *MKK2-ID* (*red*) plants, respectively. Overlapping colors and numbers mean that the corresponding transgenic lines contain the biotinylproteins and the corresponding number (supplemental Table S3*C*). *B*–*D*, the percentage of biotinylproteins over the specific number of biotinylation sites on a biotinylated protein labeled by ID, MKK1-ID, and MKK2-ID, respectively (supplemental Table S3*C*). *E*, biotin occupancy (BO) is a value for the relative amount of biotinylation, which is the ratio of the number of biotinylation sites divided by the percentage of lysine in the protein. The protein index refers to the log10-ratio of the ranks of protein occupancy for this protein. The fitting linear equations of ID, MKK1-ID and MKK2-ID labeled proteins are y = −17.88x + 46.054, y = −20.862x + 59.677, and y = −28.393x + 80.794, respectively (supplemental Table S3*D*). *F* and *G*, Volcano plots of biotin occupancy (BO) from biotinylproteomic analysis. The log2-ratio is the binary logarithmic ratio of biotin occupancy (BOR) of MKK1-ID to ID for *F* and MKK2-ID to ID for *G*. And Figure 2, *E*–*G* are related to Figure 3, *G–J*. Protein index is the rank value of log2-BOR greater than 0, equal to 0, and less than 0, respectively (supplemental Table S3*D*). *H*–*J*, schematic representation of the top two biotinylation sites for biotinylproteins labeled by ID, MKK1-ID, and MKK2-ID, respectively. *K*–*M*, Gene ontology (GO) enrichment analysis of Arabidopsis ortholog of total biotinylproteins and biotinylproteins with one biotinylation site and equal to or more than two biotinylation sites from *ID*, *MKK1-ID* and *MKK2-ID* transgenics listed in supplemental Table S3*C*, respectively. GO analysis was performed from the classification of biological processes, cellular components, and molecular functions, respectively. The total enrichment results are shown in supplemental Figs. S11–S13.

After converting the biotinylpeptides into biotinylproteins, there were 26, 63 and 176 biotinylproteins specific to ID, MKK1-ID and MKK2-ID protein sample, respectively. Only 83 biotinylproteins were found from all three transgenic plants (Fig. 2*A* and supplemental Table S3*C*). Of these biotinylproteins, 83.6%, 86%, and 86.7% had molecular weights ranging from 20 kDa to 100 kDa in *ID, MKK1-ID*, or *MKK2-ID* plants (supplemental Fig. S10, *A–C* and supplemental Table S3*C*), respectively. To compare the number of biotinylsites on these biotinylproteins, we found that approximately 80% of biotinylproteins had only one biotinylsite from all three genotypes of plants (Fig. 2, *B*–*D* and supplemental Table S3*C*).

The level and the overall distribution of biotinylation of the detected cellular proteins in *ID*, *MKK1-ID* and *MKK2-ID* transgenic plants were defined with linear regression lines between the biotin occupancy (BO) of a protein and the ranking of this biotinylprotein (Fig. 2*E* and supplemental Table S3*D*). The slope rate of the linear regression line of those biotinylproteins was 17.88, 20.862, and 28.393, respectively, for *ID*, *MKK1-ID*, and *MKK2-ID* plants, indicating that there were more overly biotinylated proteins (OBP) measured from *MKK1-ID* and *MKK2-ID* plants than that from *ID* plants. A biotinylprotein of higher BO value usually means that it is closer to the bait kinase-ID fusion protein as the prokaryotic biotin ligase only non-specifically catalyzes the biotinylation of neighboring proteins. The bait protein influences the biotinylation of protein substrates carried out by the biotin ligase (ID). The reason why the percentage of lysine in the total polypeptide length, instead of the absolute number of lysine, was used to calculate the BO value of a biotinylprotein was that the K% reduced the contribution of the higher number of lysine on the BO calculation assuming that the number of lysine residue present in a protein is positively related to the length of this biotinylprotein on average (supplemental Fig. S10, *D*–*L* and Supplemental Table S3*C*). Thus, the BO value of a biotinylprotein is a measure of the proximity of the targeted biotinylprotein to the bait fusion protein.

Consequently, the biotin occupancy ratio (BOR), which was defined by dividing the biotin occupancy (BO) value of a biotinylprotein from *MKK1-ID* and *MKK2-ID* plant to that from the control *ID* plant (supplemental Table S3*D*; See Experimental Procedure for details). The BOR values of biotinylproteins were classified into three groups (Fig. 2, *F* and *G*). The groups of biotinylproteins of positive BOR (>0) were considered to be either MKK1- or MKK2- proximal proteins (or the bait protein-influenced biotin modification) while those of negative BOR (<0) or BOR = 1 considered to be ID either ligase-proximal or background biotinylproteins independent to the effect of bait protein. To eliminate the possibility that the abundance of cellular proteins may affect the level of biotinylation of a protein, the Pearson correlation coefficient was calculated between the biotinylation site number and the abundance of this biotinylprotein determined by both iBAQ measurement and PSM count (supplemental Fig. S10, *M*–*R* and supplemental Table S3*C*). The lower Pearson correlation coefficient found for all three transgenic plants suggested that such an influence is small. Both higher BO and BOR values of a biotinylprotein should indicate the proximity and the specific association of this targeted protein to the bait fusion protein.

To examine the distribution of biotinylsites on the top biotinylproteins, the biotinylation site map was constructed for each highly biotinylprotein. It was found that there were identical biotinylproteins with similar biotinylation sites labeled by MKK1-ID and MKK2-ID on BPA1 (binding partner of ACD11, AT5G16840) (Fig. 2, *H*–*J* and supplemental Table S3*C*). Interestingly, most biotinylation sites were not located in the conserved domains. Furthermore, Gene Ontology analysis was used to analyze the molecular biological functions of these biotinylated proteins which were classified according to different numbers of biotinylation sites (Fig. 2*K* and supplemental Fig. S11). In the analysis of Biological Processes, the biotinylproteins from *MKK1-ID* and *MKK2-ID* plants with biotinylation sites >1 were particularly enriched in the negative regulation of plant-type hypersensitive response, which is a ubiquitous feature of plant/pathogen interactions under control of NLR-mediated immune response and is consistent with the role of MEKK1-MKK1/2-MPK4 in NLR protein SUMM2-mediated defense (98). By the analysis of Cellular Component, the highly biotinylated (biotinylsites >1) proteins of *MKK2-ID* plants were specifically enriched in clathrin vesicle coat, stromule and apoplast (Fig. 2*L* and supplemental Fig. S12), and clathrin vesicle coat component and apoplast were confirmed to participate in immunity response (99, 100). In terms of Molecular Function classification, the highly biotinylated (biotinylsites >1) proteins from *MKK2-ID* plants were particularly enriched in glycolate oxidase activity (Fig. 2*M* and supplemental Fig. S13). The MEKK1–MKK1/2–MPK4 cascade is a key regulator of ROS metabolism (101), and the overexpressed glycolate oxidase accumulates both hydrogen peroxide and glyoxylate in *Arabidopsis* (100). Interestingly, the function of Hsp90 and Hsp70 protein-binding was enriched only in biotinylproteins from *MKK2-ID* plants. It is reported that the transcripts of Hsp90 and Hsp70 were up regulated by pathogen infection and the expression levels of Hsp70 and MKK2 were increased by cold induction (102).

### Determination of Putative Interactors of MKK1 and MKK2 Using iBAQ-Based Quantitative Biotinylproteomics

**Fig. 3:**
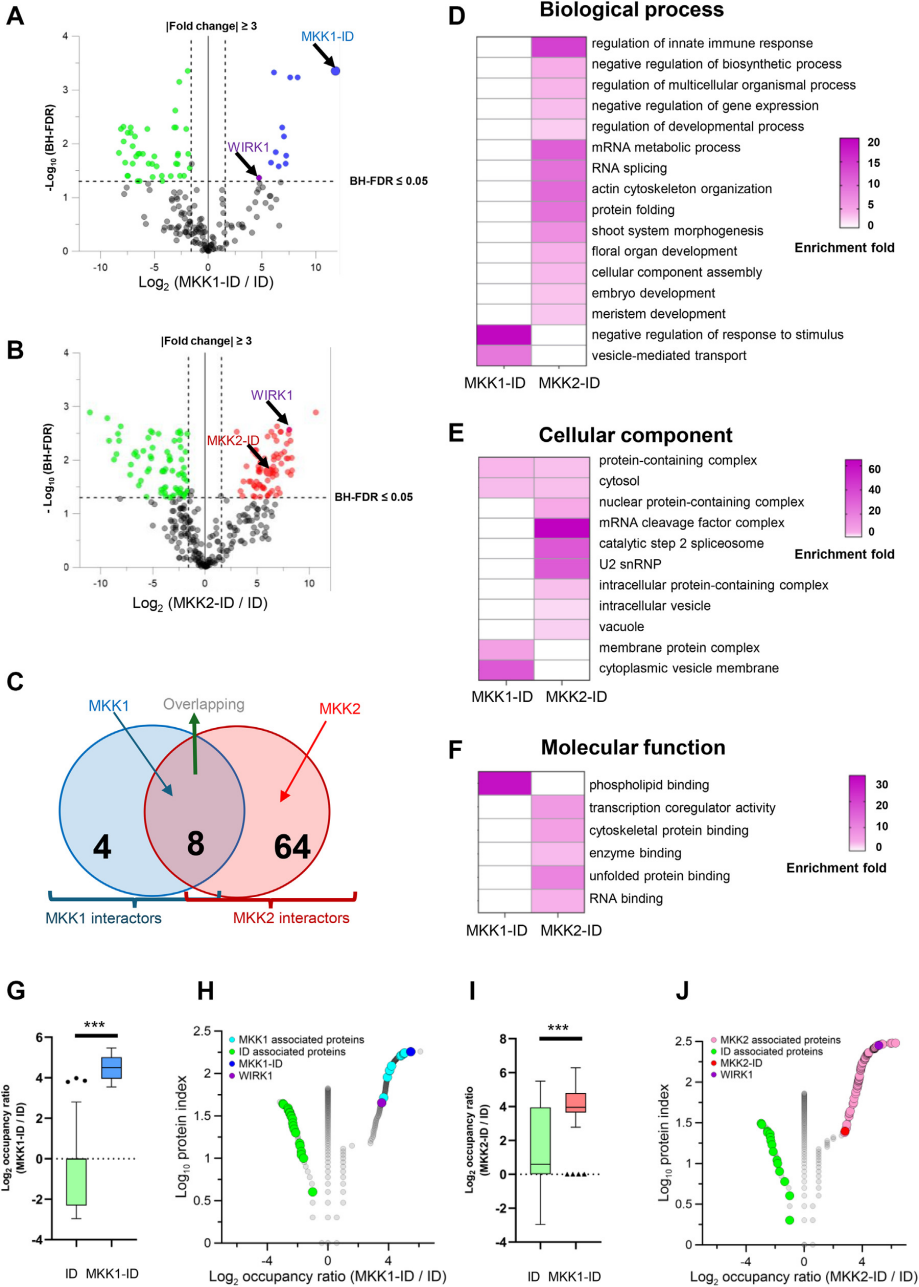
**Quantitative proteomic analysis of TurboID-based cultivars significantly biotinylproteins by different fusion proteins.***A* and *B*, volcano plots of quantitative biotinylproteomics. The Log2-ratio is the average binary logarithmic ratio of MS1 precursor ion intensity based on the intensity Based Absolute Quantification (iBAQ) method, and BH-FDR is the adjustment value of the *p*-value determined from Student’s *t* test. The *vertical and horizontal dashed line* indicates the mean ±1.58 (*i.e.*, |fold change| ≥ 3) of the distribution and the cut-off of the Benjamini–Hochberg multiple hypothesis test corrected FDR (≤0.05), respectively. *Green, blue, and red* for each quantified protein spot represent significantly quantified ID, MKK1-ID, and MKK2-ID-labeled biotinylproteins, defined as ID, MKK1-, and MKK2-associated proteins. Larger *blue* and *red* dots represent MKK1-ID and MKK2-ID, respectively. The dot pointed to by the *purple* arrow in each figure is WInd-Related Kinase 1 (WIRK1, RAF36) (supplemental Table S4, *C* and *E*). *C*, a Venn diagram showing the number of MKK1 and MKK2 interactors. *Brown area* represents the number of common interactors of MKK1 and MKK2. *D*, biological process analysis of Arabidopsis ortholog of MKK1 or MKK2 interactors, respectively. *E*, cellular component analysis of Arabidopsis ortholog of MKK1 or MKK2 interactors, respectively. *F*, molecular function analysis of Arabidopsis ortholog of MKK1 or MKK2 interactors, respectively. All GO data were from supplemental Table S5*E*. *G* and *I*, the box and whisker plots show the distribution of Log2-BOR (biotin occupancy ratio) in ID and MKK1 interactors or MKK2 interactors. Unpaired student’s *t* test was applied: ∗∗∗*p* < 0.001. *H* and *J*, volcano plots of the overlapping results between Log2-BOR from biotinylproteomic analysis and quantitative analysis results. The protein index is the rank value of Log2-BOR greater than 0, equal to 0, and less than 0, respectively. ID associated protein results with Log2-BOR less than 0 are annotated in *green*. Overlap results between MKK1 and MKK2 interactors with Log2-BOR greater than 0 are annotated in *blue* or *pink*, respectively. MKK1-ID and MKK2-ID are colored *navy blue* and *red*, and *purple* circle represents WIRK1 (RAF36, supplemental Table S5*E*). The results of an overall comparison of biotin occupancy ratio between associated proteins and non-significantly defined proteins are shown in supplemental Fig. S15.

The MS1 precursor ion intensities of both ^15^N- and ^14^N-coded biotinylproteins measured from either *MKK1-ID* or *MKK2-ID* plants were quantified against those from control *ID* plants according to iBAQ method (supplemental Table S4, *A–E*). As a result, out of the 178 biotinylproteins from both *MKK1-ID*, 12 proteins were found to be significantly evaluated as MKK1-associated proteins (BH-FDR ≤0.05 and Log2-ratio ≥1.58; Fig. 3*A* and supplemental Table S4*C*), including the MKK1-ID bait protein. As to the quantification of biotinylproteins from *MKK2-ID* plants, 72 MKK2-associated proteins, including the MKK2-ID bait protein, were significantly defined from 333 biotinylated proteins (BH-FDR ≤0.05 and Log2-ratio ≥1.58; Fig. 3*B* and supplemental Table S4*E*). These statistically and significantly associated proteins were defined as putative interactors of MKK1 and MKK2 or simply called interactors.

Out of all those MKK1 and MKK2 interactors, there were eight common interactors (Fig. 3*C*). MKK1 was one of those common interactors, implying that MKK1 might interact with MKK2. Among these MKK1 interactors, two out of 12 (16.67%) and five out of 12 (41.67%) were found to be kinases including MKK1 and WIRK1 (WInd-Related Kinase 1, an *Arabidopsis* Raf-like kinase 36 (RAF36), AT5G58950) and protein-binding proteins, respectively. Among MKK2 interactors, five out of 73 (6.85%), seven out of 73 (9.59%), and 27 out of 73 (36.99%) were found as kinases including MKK1, MKK2, WIRK1 (RAF36), MEKK1 and DYRKP-2A (Plant-Specific Dual-Specificity Tyrosine Phosphorylation-Regulated Kinase 2a, AT1G73460), transcription factors and protein-binding proteins, respectively. The discovery of interactors functioning in MAPK cascade supports the quantitative PL proteomics being able to identify novel components mediating phosphor-relay. WIRK1 is one common kinase interactor of MKK1 and MKK2, and WIRK1 (RAF36) has a homologous protein Raf-like kinase 43 (RAF43, AT3G46930) (103). When we compared the sequences and structures of the two kinases, we found that the kinase domains of the two proteins were highly identical (supplemental Fig. S14). It is reported that RAF43 is required for drought stress tolerance, salt stress tolerance, and oxidative stress tolerance (104), supporting that RAF36 could play a role in the wind signaling pathway.

By Gene Ontology enrichment of these interactors (Fig. 3, *D*–*F*), MKK1 interactors are specifically enriched in membrane structure to mediate signal transduction. SNX2B (Sorting Nexin 2B, AT5G07120), as one component of these enriched groups, was reported to module the expression of ABA-responsive genes (105) while the MKK2 interactors were particularly enriched in plant developmental processes, RNA-related processes, and various regulatory processes for different stresses. ACIP1 (Acetylated Interacting Protein 1, AT3G09980), one protein in this enriched group, is associated with punctae on the cell cortex and shows aggregation in response to pathogens through acetylation by AvrBsT (106). BOB1 (Bobber1, AT5G53400), an interactor of MKK2, is required for both development and thermotolerance despite being a small heat shock protein (107). Loss of function of BOB1 presented are embryo lethal phenotype, and homozygous mutants of *mekk1* and *mpk4* also showed lethal phenomena (28). To our surprise, when comparing all the interactors of MKK1/2-ID with those of ID, in terms of their biotin occupancy ratios (BORs), the MKK1 or MKK2 interactors were all of a significantly higher BOR as compared to that of ID biotin ligase produced biotinylproteins (*p < 0.01*; Fig. 3, *G*–J, supplemental Fig. S15 and supplemental Tables S3*D* and S5*E*). It is therefore concluded that the biotin occupancy ratios (BORs) between biotinylproteins originated from the bait fusion protein plants and those from the ID ligase expression plants are able to determine the putative interactors of a bait protein, either in parallel or in combination with the significant Log2 ratios of these biotinylproteins.

### Determination of MKK1- and MKK2-specific Interactors

**Fig. 4:**
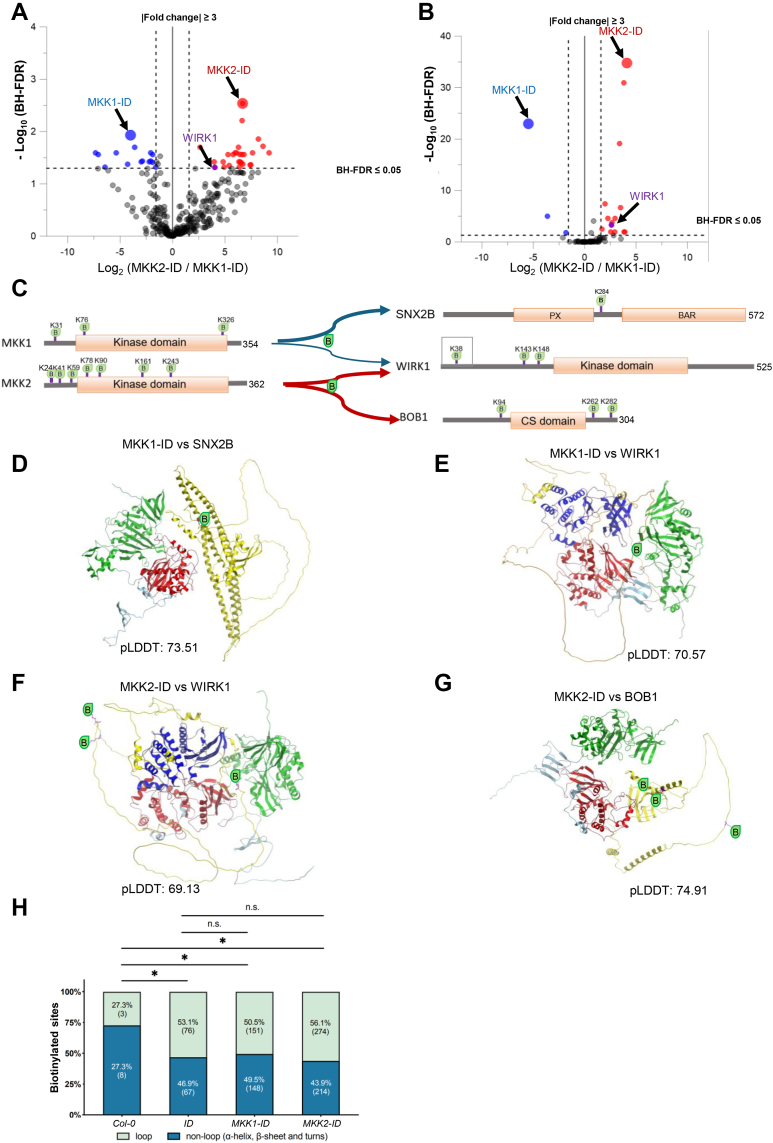
**Quantitative proteomic analysis of TurboID-based cultivars significantly biotinylproteins by MKK1-ID and MKK2-ID.***A* and *B*, volcano plots of quantitative biotinylproteomics between MKK2-ID and MKK1-ID. The Log2-ratio is the average binary logarithmic ratio of MS1 precursor intensity (*A*) and of MS1 isotopologue areas (*B*) of biotinylproteins, and BH-FDR is the adjustment value of *p*-value determined from Student’s *t* test. The *vertical and horizontal dashed lines* indicate the mean ±1.58 (*i.e.*, |fold change| ≥ three) of the distribution and the cut-off of Benjamini–Hochberg multiple hypothesis test corrected FDR (≤0.05), respectively. *Blue and red dots* represent quantified significantly MKK1- and MKK2-specific interactors. *Larger blue and red dots* represent MKK1-ID and MKK2-ID, respectively. The *dot* pointed to by the *purple arrow* in each figure is WIRK1 (RAF36, supplemental Table S5, *B* and *D*). The comparison results of the two quantification methods are shown in the supplemental Fig. S16*A*. *C*, schematic representation of biotinylation sites for MKK1 and MKK2 interactors. The *blue and red lines* indicate that the interactors belong to MKK1 and MKK2, respectively. *Thick lines* are used to annotate the significantly quantified specific proteins. The biotinylation site marked by *gray square* is common biotinylation site in MKK1-ID and MKK2-ID. *D*–*G*, the predicted 3D complex structures of the MKK1 with SNX2B, MKK1 with WIRK1 (RAF36), MKK2 with WIRK1 (RAF36), and MKK2 with BOB1. MKK1 and MKK2 are colored *gray* and TurboID at the C-terminus of MKK1 or MKK2 is annotated in *green*. Other interactors are colored *yellow*. The kinase domains of MKK1 or MKK2 are annotated in *red* and those of interactors are annotated in *blue*. Biotinylated lysine residues are annotated in *purple*. AlphaFold uses the score of pLDDT to estimate the confidence of the predicted model. According to AlphaFold, regions with pLDDT between 70 and 90 are expected to be well modeled, while regions with pLDDT between 50 and 70 have low confidence and should be treated with caution. The predicted structures of MKK1-ID, MKK2-ID, SNX2B, WIRK1 and BOB1 are shown in supplemental Fig. S16*B*. *H*, The bar chart represents a comparison of the percentage of biotinylated sites located at the loop region of the secondary structure of biotinylated proteins on four different plants (Col-0, ID, MKK1-ID and MKK2-ID). The *green and blue bar* indicates the percentage of biotinylated sites located at the loop and non-loop region (α-helix, β-sheet and turns), respectively. The number in parentheses shows the exact number of biotinylated sites. The fisher exact test was applied to statistically compare the percentage of biotinylated sites located on loop region between different plants. The n.s and ∗ represents *p*-value ≥ 0.05 and *p*-value < 0.05, respectively (supplemental Tables S3*C* and S6).

In the literature, it has been reported that MKK1 and MKK2 have an overlapping function (108). Thus, from the 84 interactors of MKK1 and MKK2 determined above, we further determined either MKK1- or MKK2-specific interactors using iBAQ quantification methods. There were 2 and 15 of MKK1- and MKK2-specific interactors identified, respectively (Log2-ratio ≥ ∣ ±1.58∣; Fig. 4*A*; supplemental Table S5*B*). In addition, we applied the SILIA-based quantification in E2 using SQUA-N software, and there were one and five significantly selected MKK1- and MKK2- specific interactors, respectively (Fig. 4*B* and supplemental Table S5*D*). Combination of results of two biotinylproteomic quantification methods (Table 1, supplemental Fig. S16*A* and supplemental Table S5*E*), there was one and three significantly selected interactors of MKK1 and MKK2, respectively, that were commonly identified by both XIC and MS1 precursor ion intensity-based approaches. Both BOB1 and WIRK1 (RAF36) were defined as MKK2-specific interactors, meaning that these interactors might have a higher frequency of interacting transiently with the bait protein MKK2 than with MKK1.

**Table 1:** Summary of MKK1 and MKK2 interactors by SILIA- and iBAQ-based quantification

Accession no.^a^	Protein description^b^	MKK1 interactors^c^	MKK2 interactors^d^	MKK1 specific interactors^e^	MKK2 specific interactors^f^	GO Categories^g^	GO Categories^h^
AT5G07120	Sorting nexin 2B (SNX2B)	Y		Y		vesicle-mediated transport; phospholipid binding; cytoplasmic vesicle membrane	
AT4G25680	PPPDE putative thiol peptidase family protein	Y					
AT5G13260	Unknown protein	Y					
AT5G09300	e1a2 subunit of branched chain ketoacid dehydrogenase (BCKDH) complex (E1A2)	Y					
AT4G26070	Mitogen activated protein kinase kinase 1 (MKK1)	Bait	Y	Bait	Bait		
AT4G17720	RNA-binding (RRM/RBD/RNP motifs) family protein (BPL1)	Y	Y			negative regulation of response to stimulus	regulation of innate immune response; cytosol
AT2G36480	Pre-mRNA cleavage complex 2 Pcf11-like protein	Y	Y				mRNA metabolic process; RNA binding; enzyme binding; nuclear protein-containing complex; mRNA cleavage factor complex; protein-containing complex
AT2G43160	Involved in plant trans-Golgi network (TGN) transport (EPS2)	Y	Y			vesicle-mediated transport; cytoplasmic vesicle membrane; phospholipid binding	protein-containing complex; cytosol
AT3G16760	Tetratricopeptide repeat (TPR)-like superfamily protein	Y	Y				
AT1G13190	RNA-binding (RRM/RBD/RNP motifs) family protein	Y	Y				mRNA metabolic process; RNA binding; nuclear protein-containing complex; mRNA cleavage factor complex
AT5G03660	Transcriptional activator (DUF662)	Y	Y				
AT5G58950^i^	A group C Raf-like protein kinase (RAF36); Wind-Related Kinase 1 (WIRK1)	Y	Y		Y	negative regulation of response to stimulus	cytosol
AT5G53400	BOBBER1, HSP20-like chaperones superfamily protein (BOB1)		Y		Y		protein folding; embryo development; unfolded protein binding; floral organ development; regulation of developmental process; meristem development; shoot system morphogenesis
AT3G12640	RNA binding (RRM/RBD/RNP motifs) family protein		Y				RNA binding; mRNA metabolic process
AT3G21790	UDP-Glycosyltransferase superfamily protein		Y		Y		cytosol
AT2G38710	AMMECR1 family		Y				
AT5G43830	Aluminium induced protein with YGL and LRDR motifs		Y				cytosol
AT2G25080	Glutathione peroxidase 1 (ATGPX1)		Y				cellular component assembly
AT4G08500^i^	MAPK/ERK kinase kinase 1 (MEKK1)		Y				meristem development; enzyme binding
AT2G32600	Hydroxyproline-rich glycoprotein family protein		Y				nuclear protein-containing complex; RNA splicing; catalytic step 2 spliceosome; U2 snRNP; mRNA metabolic process; protein-containing complex
AT4G11260	AtSGT1a and AtSGT1b are functionally redundant in the resistance to pathogenes (ATSGT1B)		Y				regulation of multicellular organismal process; regulation of developmental process; intracellular protein-containing complex; embryo development; protein-containing complex; cytosol
AT1G09310	ABA responsive trichome formation regulator (SVBL)		Y				cytosol
AT3G13570	SC35-like splicing factor 30A (AT-SCL30A)		Y		Y		RNA binding; RNA splicing; mRNA metabolic process
AT2G45990	Ribosomal RNA small subunit methyltransferase G		Y				
AT1G30070	SGS domain-containing protein		Y		Y		enzyme binding; cytoskeletal protein binding
AT4G21660	Proline-rich spliceosome-associated (PSP) family protein (BRX)		Y		Y		nuclear protein-containing complex; RNA splicing; catalytic step 2 spliceosome; U2 snRNP
AT4G04885	PCF11P-similar protein 4 (PCFS4)		Y				mRNA cleavage factor complex; regulation of developmental process; nuclear protein-containing complex; enzyme binding; RNA binding
AT3G52230	Unknown protein		Y				
AT5G23080	SWAP (Suppressor-of-White-APricot)/surp domain-containing protein (TGH)		Y		Y		negative regulation of gene expression; RNA binding
AT5G26360	TCP-1/cpn60 chaperonin family protein (CCT3)		Y				intracellular protein-containing complex; protein folding; unfolded protein binding
AT3G17300	COMPLEX 1 LYR-like protein (EMB2786)		Y				
AT2G41370	Ankyrin repeat family protein/BTB/POZ domain-containing protein (BOP2)		Y		Y		regulation of innate immune response; shoot system morphogenesis; floral organ development; meristem development
AT3G09980	A microtubules-associated protein required for bacterial immunity (ACIP1)		Y		Y		cytoskeletal protein binding; regulation of innate immune response
AT2G39900	A member of the Arabidopsis LIM proteins: a family of actin bundlers with distinct expression patterns (WLIM2A)		Y				actin cytoskeleton organization; cytoskeletal protein binding
AT3G49010	60S ribosomal protein L13 (EL13Z)		Y				RNA binding
AT2G33730	Homolog of the DEADbox pre-mRNA splicing factor Prp28 which regulates abundance of miRNA (SMA1)		Y		Y		nuclear protein-containing complex; RNA splicing; catalytic step 2 spliceosome; mRNA metabolic process; RNA binding
AT1G15280	CASC3/Barentsz eIF4AIII binding (BTZ2)		Y				nuclear protein-containing complex; negative regulation of gene expression; RNA binding; mRNA metabolic process
AT1G54080	Oligouridylate-binding protein 1A (UBP1A)		Y		Y		RNA binding
AT4G10070	KH domain-containing protein (ATKH18)		Y				RNA binding
AT5G05610	Alfin-like 1 (AL1)		Y		Y		transcription coregulator activity
AT4G29810	mitogen activated protein kinase kinase 2 (MKK2)	Bait		Bait	Bait		
AT3G08740	Elongation factor P (EF-P) family protein		Y				embryo development
AT2G30710	Ypt/Rab-GAP domain of gyp1p superfamily protein		Y				
AT5G52580	RabGAP/TBC domain-containing protein		Y				
AT5G14240	Thioredoxin superfamily protein		Y				protein folding
AT4G22670	HSP70-interacting protein 1 (ATHIP1)		Y				protein folding; nuclear protein-containing complex
AT5G07980	A specific subunit of SYD-associated SWI/SNF (SAS) complexes		Y				regulation of developmental process; negative regulation of biosynthetic process
AT2G37190	Ribosomal protein L11 family protein (UL11Z)		Y				RNA binding
AT1G02080	Scaffold protein in the CCR4-NOT complex (NOT1)		Y				negative regulation of biosynthetic process; negative regulation of gene expression; intracellular protein-containing complex; mRNA metabolic process
AT1G73460	Plant-specific dual-specificity tyrosine phosphorylation-regulated kinase 2A (DYRKP-2A)		Y				
AT5G44200	CAP-binding protein 20 (ATCBP20)		Y		Y		RNA splicing; negative regulation of gene expression; RNA binding; intracellular protein-containing complex; mRNA metabolic process
AT5G04280	One of the zinc finger-containing glycine-rich RNA-binding proteins involved in cold tolerance (ATRZ-1C)		Y		Y		negative regulation of biosynthetic process; RNA binding
AT4G15900	Pleiotropic regulatory locus 1 (PRL1)		Y				RNA splicing; catalytic step 2 spliceosome; negative regulation of biosynthetic process; intracellular protein-containing complex; embryo development; nuclear protein-containing complex
AT2G16860	GCIP-interacting family protein (ATSYF2)		Y				catalytic step 2 spliceosome; nuclear protein-containing complex; regulation of developmental process
AT2G41350	AUG1, likely to be in an augmin-like complex with AUG3 (ATAUG1)		Y				
AT3G05520	Capping protein A (ATCPA)		Y				actin cytoskeleton organization; cytoskeletal protein binding
AT1G43850	SEUSS transcriptional co-regulator (SEU)		Y				floral organ development; transcription coregulator activity; negative regulation of biosynthetic process; embryo development; regulation of developmental process
AT3G60820	N-terminal nucleophile aminohydrolases (Ntn hydrolases) superfamily protein (PBF1)		Y				intracellular protein-containing complex
AT3G48590	Nuclear factor Y, subunit C1 (ATHAP5A)		Y				nuclear protein-containing complex; regulation of developmental process
AT5G41520	RNA binding Plectin/S10 domain-containing protein (RPS10B)		Y				shoot system morphogenesis; RNA binding; regulation of developmental process; meristem development
AT2G23760	BEL1-like homeodomain 4 (BLH4)		Y				shoot system morphogenesis
AT1G73030	SNF7 family protein (VPS46.2)		Y				embryo development
AT2G22475	GRAM domain family protein (GEM)		Y				
AT1G10200	A member of the Arabidopsis LIM proteins (WLIM1)		Y				RNA binding; cytoskeletal protein binding; actin cytoskeleton organization
AT1G33050	Unknown protein		Y				
AT5G08660	D-lactate dehydrogenase (DUF668) (PSI3)		Y				
AT3G21865	Peroxin 22 (PEX22)		Y				
AT3G52300	ATP synthase D chain, mitochondrial (ATPQ)		Y				
AT3G06480	RNA helicase 40 (RH40)		Y		Y		RNA binding; regulation of developmental process
AT1G30480	D111/G-patch domain-containing protein (DRT111)		Y				RNA splicing; mRNA metabolic process
AT5G64270	Splicing factor, putative		Y				catalytic step 2 spliceosome; U2 snRNP; RNA binding; RNA splicing; nuclear protein-containing complex; mRNA metabolic process
AT3G09350	One of the Arabidopsis orthologs of the human Hsp70-binding protein 1 (HspBP-1) and yeast Fes1p (FES1A)		Y				
AT3G61140	Shaggy-like kinase 31 (SK31)		Y				embryo development; nuclear protein-containing complex
AT1G73350	Ankyrin repeat protein		Y				
AT5G14540	Basic salivary proline-rich-like protein (DUF1421) (FLOE2)		Y				
AT3G11200	Alfin-like 2 (AL2)		Y		Y		transcription coregulator activity

a The accession number of the protein.

b The protein annotation is based on the description of TAIR10.

c MKK1 interactors were defined as MKK1-significantly associated proteins by iBAQ-based quantitative comparison of MKK1-ID to ID.

d MKK2 interactors were defined as MKK2-significantly associated proteins by iBAQ-based quantitative comparison of MKK2-ID to ID.

e MKK1-specific interactors were obtained from proteins for which MKK1 was significantly and specifically associated by quantitatively comparing iBAQ-based and SILIA-based MKK2-ID with MKK1-ID and then filtering the results for MKK1 interactors.

f MKK2-specific interactors were obtained from proteins for which MKK2 was significantly and specifically associated by quantitatively comparing iBAQ-based and SILIA-based MKK2-ID with MKK1-ID and then filtering the results for MKK2 interactors.

g Gene Ontology categories of proteins were the result of GO enrichment for MKK1 interactors, consistent with Figure 3, *D*–*F*.

h Gene Ontology categories of proteins were the result of GO enrichment for MKK2 interactors, consistent with Figure 3, *D*–*F*.

i Stands for that the interactor has been validated in a published paper, Y represents that the protein meets the criteria for that column, Bait in this study represents the bait proteins MKK1 and MKK2, which was fused to TurboID.

The biotinylsites were depicted on those highly biotinylproteins (Fig. 4*C*). They included those on both bait proteins MKK1-ID (K31, K76 and K326) and MKK2-ID (K24, K41, K59, K78, K90, K161 and K243) as well as that on the interactors, SNX2B (K284) specific to MKK1, and BOB1 (K94, K262 and K282) and WIRK1 (K143 and K148) specific to MKK2 (Fig. 4, *D*-*G*), but WIRK1 (K38 of RAF36) was a common biotinylsite for MKK1 and MKK2. WIRK1 (RAF36) was determined to be an interactor for both MKK1 and MKK2. However, it seemed to be labeled to a higher biotinylation level in *MKK2-ID* plants, thus being considered to be an MKK2-specific interactor.

By profiling those interactors of MKK1 and MKK2 (those bait proteins significantly biotinylated target proteins and were free of background biotinylproteins and ID interactors), it was found that the biotinylation sites were enriched in the non-conserved domains of these interactors. The structures of MKK1/2 with their interactors were predicted by AlphaFold and were drawn by ICM-Browser Pro (Fig. 4, *G*–*J* and supplemental Fig. S16*B*) (109, 110). The spatially close positions of biotinylated lysine residue on the target interactors suggest that the target proteins interact with the bait fusion enzymes from a fixed direction or through the docking sites of bait kinase and its substrates. It seems that the short linker existing in between ID biotin ligase and MKK1/2 kinases indeed produced two distinct protein domains mutually independent to each other structurally. However, the linker does not seem to allow the ID biotin ligase to swing around and it can only label interactors through a specifically oriented direction (Fig. 4, *D*–*G*).

By integrating the biotinylation site information from PTM proteomics and the information from the predicted 3D structures of target proteins using Alphafold, it was interesting to find that most of the biotinylation sites were located at the loop region (Fig. 4*H*). From *ID*, *MKK1-ID* and *MKK2-ID* transgene expression plants, 50.5 to 56.1% of the total biotin sites were found to locate in the loop region of biotinylproteins (Fig. 4*H* and supplemental Table S3*C*). The biotinylation statuses on four different types of structural domains, α-helix, β-sheet, turn, and loop, of biotinylproteins were compared between the transgenic and the wild-type plants. The plant endogenous biotinylproteins, such as methylcrotonyl-coenzyme A (CoA) carboxylase (111), may have a specific *in vivo* biotin-labeling pattern according to the enzyme-substrate interaction principles (112). Statistical Fisher Exact test analysis of the biotinylation site distribution on biotinylproteins of both the transgenic plants and the wild type plants suggested that there was a significant difference in the level of loop biotinylation between the TurboID-labeled and the endogenous enzyme-labeled proteins (Fig. 4*H* and supplemental Tables S3*C* and S6) (47, 113, 114).

### Modeling of the Putative Local Interactome of MKK1 and MKK2

**Fig. 5:**
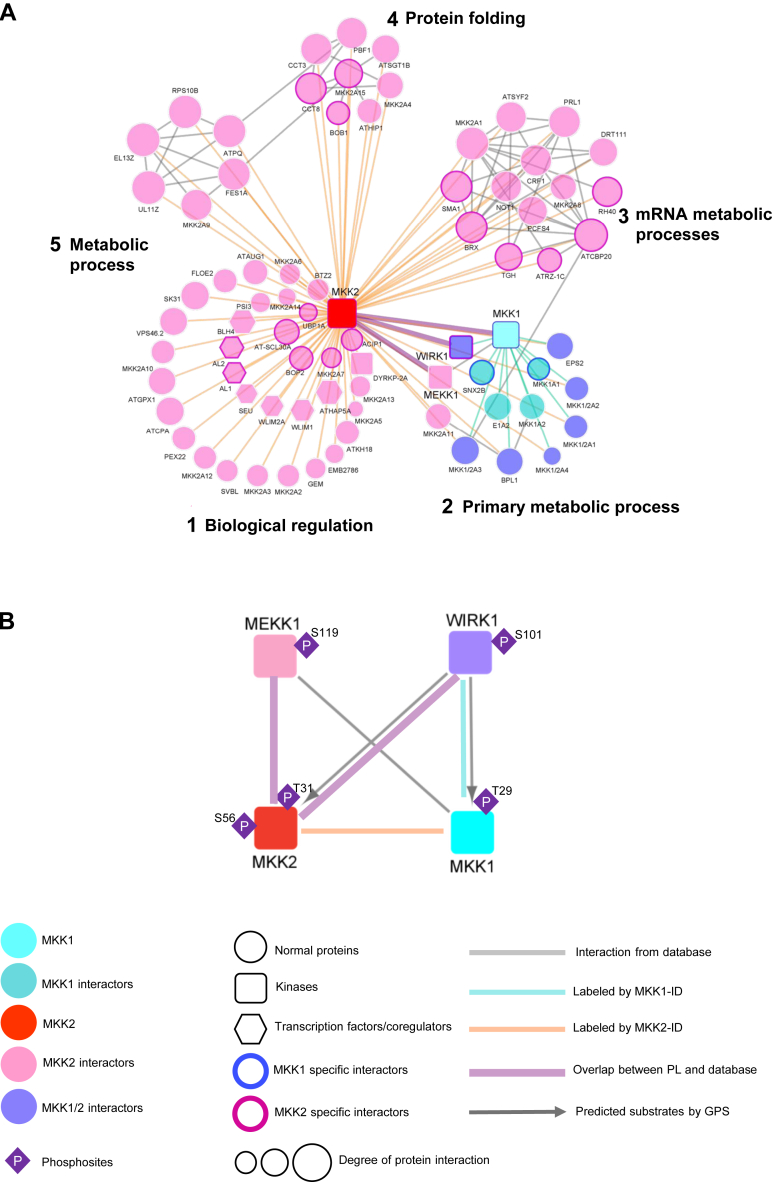
**The module-module communication network and functional characterization.***A*, volvox graphic representation of the putative protein-protein interaction. All nodes represent significantly defined interactors of MKK1 or MKK2. The *navy blue, blue, red, pink and purple nodes* stand for MKK1, MKK1 interactors protein, MKK2, MKK2 interactors and common interactors, respectively. Nodes bordered in *navy blue or pink* indicate that they are specific interactors of MKK1 or MKK2. Circular node, square, and hexagon represent normal protein, kinase and transcription factor, respectively. The size of the node indicates the level of degree of protein interaction (log10-DPI). The *grey line* shows the interaction from STRING database with a confidence score ≥0.7 and BioGrid database. The *orange, blue, and brown lines* represent the interaction from MKK1, MKK2, and both databases and PL experiment, respectively. The module analysis was performed by MONET, and Gene Ontology analysis was used to name each module in the network by GENEONTOLOGY. All proteins in a module are dashed into a bubble (supplemental Table S7, *A* and *B*). *B*, putative kinase and substrates interacting network in this study. *Navy blue, red, pink and purple squares* stand for MKK1, MKK2, MEKK1 and WIRK1 (RAF36), respectively. The *grey line* shows the interaction from STRING database with confidence score ≥0.7 and BioGrid database. The *orange, blue and brown lines* represent the interaction from MKK1, MKK2 and both databases and PL experiment, respectively. *Gray lines with arrows* indicate kinase-substrate relationships, with arrows pointing to substrates. The putative relationship between kinases and substrates was predicted by GPS 5.0 and the workflow of data process is shown in supplemental Fig. S17. The P in *purple diamonds* stands for the a phosphosite (supplemental Tables S8 and S9).

To identify the putative local interactomes of MKK1 and MKK2 using the biotinylproteomics data, both the published and the predicted protein-protein interaction (PPI or called edge) information was extracted from BioGRID and STRING (interaction score >0.7) databases (supplemental Table S7). Of the 76 total interacting protein components, we identified 56 edges for 36 (47%) interactors from these two databases. At the same time, ID-based PL biotinylproteomics provided 82 edges for MKK1 and MKK2, of which only three edges were published before and the rest 78 edges were novel or called non-confirmative edges (Fig. 5*A* and supplemental Table S7, *A* and *B*). All interactors (or called nodes) and edges were subjected to modularization analysis by MONET software (68, 80). As a result, five modules were generated. Module one and module two were classified as the biological regulation (19/35) and the primary metabolic process (8/14), respectively, around MKK1 and MKK2. Modules 3, four, and five revealed denser interactions and classified them as mRNA metabolic process (10/14), protein-folding (5/8), and metabolic process (4/6), respectively. The size of the nodes represents the extent of protein-protein interactions across the total databases, and we found UL11Z (Ribosomal Protein UL11Z, AT2G37190), EL13Z (Ribosomal Protein EL13Z, AT3G49010) and RPS10B (Ribosomal Protein S10E B, AT5G41520) to be the top three proteins with the most interactors fetched from the published databases. All three candidates belonged to ribosomal proteins and they were MKK2 interactors located in module 5.

To elucidate MKK1- and MKK2-centered MAPK cascade, we only selected the kinases, predicted the putative substrates using the phosphosites extracted from the biotinylproteins and reanalyzed touch-inducible phosphoproteomics data (21) through GPS 5.0 (Fig. 5*B*, supplemental Fig. S17 and supplemental Tables S8 and S9). All of the predicted kinase-substrate pairs were filtered through BioGRID, STRING, and the PL biotinylproteomic results. In the end, WIRK1 was speculated to act upstream of MKK1 and MKK2 and catalyze the phosphosite T29/T31 of these two touch-responsive kinases. Provided that MKK1 and MKK2 are substrates of MEKK1 during the cold response and/or pathogen resistance (98, 108), that the yeast two-hybrid and BiFC (Bimolecular fluorescence complementation) (37) confirmed the interactions among MKK1, MKK2, and MEKK1 (27, 115) and that the interaction of RAF36 with MKK2 had been validated by yeast two-hybrid experiments (116, 117), we therefore model that the WIRK1 (RAF36) and MEKK1 -> MKK1/2 functions as MAPK cascade to transduce the wind force signals down to thigmomorphogenesis.

### Functional Validation of RAF36 in Wind Force Signaling

**Fig. 6:**
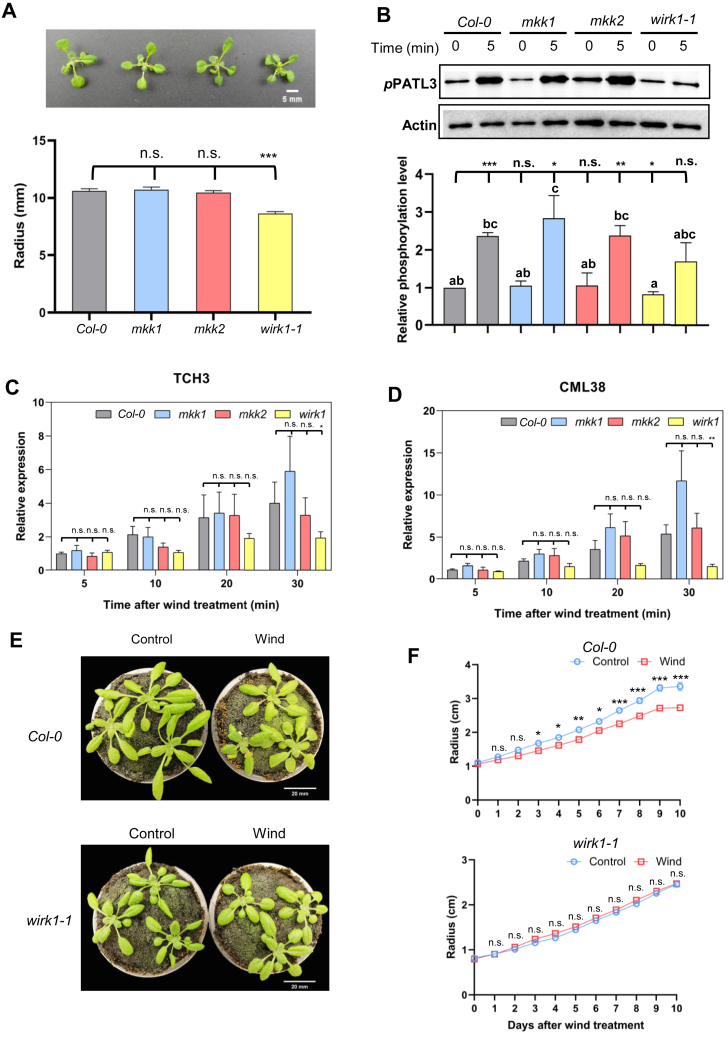
**Functional analysis of WIRK1 (RAF36).***A*, plant morphology (*upper panel*) and rosette radius (*lower panel*) of the Arabidopsis *Col0*, *mkk1*, *mkk2* and *wirk1-1 (raf36-1)*. Unpaired Student’s *t* test was performed. The genotyping result of *wirk1-1* (raf36-1) is shown in supplemental Fig. S18. *B*, the enhanced phosphorylation level of PATL3 in Arabidopsis *Col0*, *mkk1 mkk2* and *wirk1-1* (raf36-1) after 5-min wind treatment. The *upper blot* was one of four biological replicates, and the lower bar graph showed statistical analysis by Student’s *t* test. Homogeneity of variance in each panel with multiple (more than two) values was analyzed using Tukey’s range test. The specificity of the antibody was verified and shown in supplemental Fig. S18. Four biological replicates were performed, and all results (including the complete blot of *B*) are shown in supplemental Fig. S19. *C* and *D*, TCH3 or CML38 was induced within 30 min after 1-min wind treatment in *Col0*, *mkk1*, *mkk2* and *wirk1-1* (raf36-1) plants. mRNA levels were quantified by RT-qPCR and analyzed by Student’s *t* test based on four biological replicate experiments. All primers are summarized in supplemental Table S10. *E*, phenotypes of 10-days-treated plants of *Col0* and *wirk1-1* (raf36-1) mutant plants. *F*, curves of changes in plant size of control plants and wind-treated plants for 10 days after wind treatment. 16-day-old plants grown in soil were treated with or without wind stimulation. Four biological replicates were performed, and this is the pooled result of the four biological replicates (total n > 50 for each genotype per treatment group), the morphology and results of the four biological replicates are shown in supplemental Figs. S20 and S21. The statistical test was performed with student’s *t* test. Data are means ± standard error (SE). ∗*p* < 0.05, ∗∗*p* < 0.01, ∗∗∗*p* < 0.001 and n.s. (not significant) *p* ≥ 0.05. Different letters indicated significant differences at the 5% level based on Tukey’s range test.

To validate whether RAF36 (WIRK1) participates in wind force-triggered thigmomorphogenesis, we first screened out two T-DNA homozygous mutant plants *raf36-1* (*wirk1-1*) and *raf36-2* (*wirk1-2*) for the *RAF36* (*WIRK1*) gene (supplemental Fig. S18, *A–C*). We then compared the morphology of 16-day-old *mkk1*, *mkk2*, and *raf36-1* (*wirk1-1*) mutants to *Col-0*. *Raf36-1* (*Wirk1-1*) mutant exhibited a smaller rosette size, whereas both *mkk1* and *mkk2* single mutant and the wild type plant displayed a normal rosette size (Fig. 6*A*).

The quantitative phosphoproteomic analysis of 40-s mechanically (touch) stimulated *Arabidopsis* has provided a group of phosphoproteins responsive to mechano-stimulation (21, 55). Reanalysis of the published MS data (ProteomExchange, PXD006180 and PXD006181) using SQUA-N quantification software revealed that three kinases MKK1, MKK2, MEKK1 have an increased phosphorylation level upon mechanical stimulation. Another protein, PATL3 (Patellin-3, AT1G72160), which could bind phosphoinositides (118), has significantly increased its phosphorylation by 5.65 folds at S108 residue in response to touch stimulation (supplemental Table S8).

To validate these phosphorylation changes, we generated polyclonal antibodies against the phosphorylated S108 site of PATL3 and confirmed the specificity of the antibodies by comparing the phosphorylation level of *patl3* mutant line, which was unable to produce PATL3 protein, with that of the wild-type upon touch-stimulation (supplemental Fig. S18, *D–G*). Immunoblot-based quantitation of phosphorylation using the anti-*p*PATL3 polyclonal antibodies demonstrated that the phosphorylation of PATL3 increased 2.4 ± 0.1 folds in response to wind force induction in *Col-0* (Fig. 6*B* and supplemental Fig. S19). Upon wind force stimulation, PATL3 phosphorylation was increased by 2.8 ± 0.5, 2.4 ± 0.2, and 1.7 ± 0.4-folds in *mkk1*, *mkk2*, and *raf36-1* (*wirk1-1*) mutant plants, respectively. The same experiment was also performed on *raf36-2* (*wirk1-2*) mutant plant with three biological replicates. These results indicated that the phosphorylation level of PATL3 in *raf36-2* under wind stimulation exhibited a similar trend to the phosphorylation level in *raf36-1* (*wirk1-1*) mutant plants (supplemental Fig. S19, *E–G*). The partial blockade of phosphorylation of PATL3 under wind force stimulation suggested that RAF36 (WIRK1) is required for wind-stimulated signal transduction either through the direct or indirect phosphorylation of PATL3.

To investigate if RAF36 (WIRK1) might play a role in wind force-induced gene expression, we selected two mechano-responsive genes, *TCH3* (*TOUCH 3*, AT2G41100) and *CML38* (*Calmodulin-like protein 38*, AT1G76650) (19, 21) to perform RT-qPCR analysis on the wind-induced transcription of these two genes (Fig. 6, *C* and *D*). TCH3 was induced 4.1 ± 0.8, 5.3 ± 1.1, 3.4 ± 0.6, and 2.1 ± 0.2-fold at 30 min following the application of a minute of wind stimulus in wild type, *mkk1*, *mkk2*, and *wirk1*-*1* (*raf36-1*), respectively, in comparison with its expression at 0 min. The expression of *CML38* gene was also increased 5.4 ± 1, 11.7 ± 3.3, 6.1 ± 1.6 and 1.5 ± 0.2-fold at 30 min of wind induction in the wild type, *mkk1*, *mkk2* and *wirk1-1* mutant, respectively.

Taken together, WIRK1 plays a role in wind force-induced phosphorylation change on PATL3 and in gene expression of *TCH3* and *CML38*. However, a similar analysis performed on *patl3* mutant revealed that it plays no dramatic role in the regulation of *TCH3* and *CML38* gene expression (supplemental Fig. S20, *A* and *B*). These results indicated that the RAF36-regulated phosphorylation of PATL3 protein and the gene expression of *TCH3* and *CML38* may go through separate signal transduction pathways.

To study the role of RAF36 in mediating wind force triggered thigmomorphogenesis, 16-day-old *raf36-1* (*wirk1-1*) mutant and *Col-0* plants were treated with wind loading for 16 h per day at a velocity of 4 m/sec according to the published wind treatment condition (7, 119, 120). 10 days after wind-blowing, the rosette sizes of plants were measured and compared between the treated and untreated plants. Wind-blown plant rosette had a radius ranging from 2.37 to 3.33 cm, while the untreated control plant rosette had a radius of 2.83 to 4.32 cm in *Col-0* based on four biological replicates (supplemental Figs. S20*C* and S21). As to *raf36-1* (*wirk1-1*) mutant, the average sizes of wind-blown plant rosettes were between 2.24 to 2.84 cm, and the radius of untreated plants was 2.32 to 2.74 cm. Consistent with these results, the growth curves of both genotypes over a 10-day period when the air-blown was applied to treat the wind-blown group (Fig. 6, *E* and *F*), *Col-0* grew significantly slower than the untreated plants from day 3 onwards, whereas there was no significant difference between the treated and the untreated plants of *raf36-1* (*wirk1-1*). These results strongly suggested that *raf36-1* (*wirk1-1*) mutant blocks the wind drag force-triggered thigmomorphogenesis and RAF36 (WIRK1) is required for normal wind mechano-response.

### The Role of RAF36 in Shoot Gravitropism

**Fig. 7:**
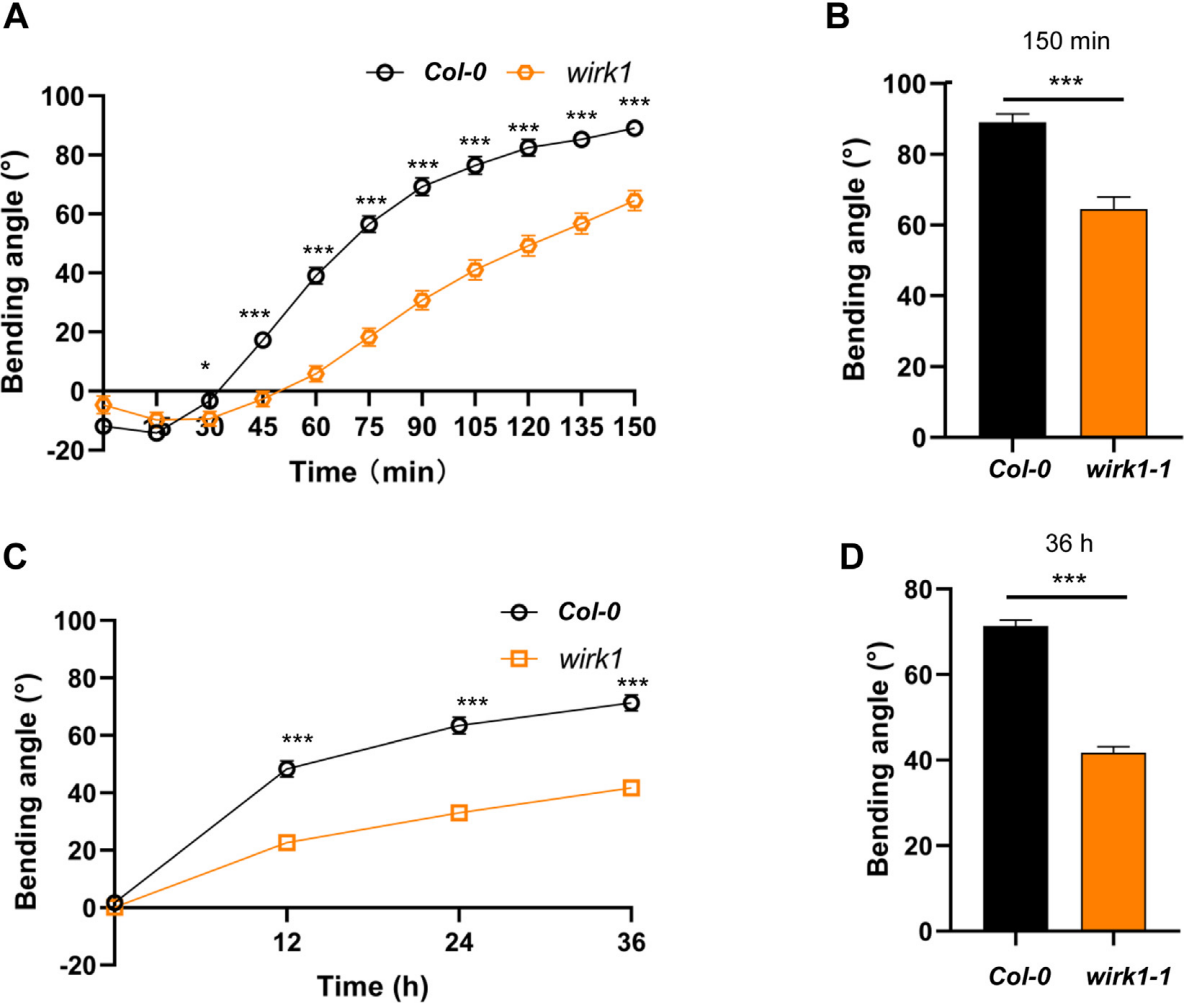
**Gravitropic response of *Col-0* and *wirk1-1 (raf36-1)* mutant plants.***A*, inflorescence stem bending curves of *Col-0* and *wirk1-1 (raf36-1)* plants within 2.5 h of gravity vector change. *B*, Infloresecence stem bending of the *Col-0* and *wirk1-1 (raf36-1)* mutant plants after gravity alteration for 150 min. Three biological replicates were performed, and this is the summary result of all replicates (n > 20 for each genotype in each replicate). The results of the three biological replicates are shown in supplemental Figs. S22*A* and S23. *C*, hypocotyl bending curves of *Col0* and *wirk1-1 (raf36-1)* plants within 36 h of gravity vector change for three biological replicates. *D*, hypocotyl bending of the *Col-0* and *wirk1-1 (raf36-1)* mutant plants after gravity alteration for 36 h. Three biological replicates were performed, this is the summary result of all replicates (n > 40 for each genotype in each replicate). The results of the three biological replicates are shown in supplemental Figs. S22*B* and S24. The statistical test was performed employing the student’s *t* test. Significance of *p* < 0.05, *p* <0.01 and *p* < 0.001 are shown as ∗, ∗∗ and ∗∗∗, respectively.

In literature, it was reported that PATL3 was significantly upregulated in phosphoprotein upon the gravity vector change (70). As gravity force is also considered another type of mechanical signal, and WIRK1 Raf-like kinase acts upstream of PATL3 phosphorylation, we investigated the role of RAF36 (WIRK1) in gravitropism. To that end, inflorescence stems were placed horizontally by rotating the glass bottle when the inflorescence grew to 2 to 5 cm (70). After changing the gravity vector for 2.5 h, the inflorescence stem of *Col-0* was bent by about 90° in the dark, while the inflorescence stems of *wirk1-1* mutant plants only bent by 60° under the same dark condition (Fig. 7, *A* and *B* and supplemental Figs. S22*A* and S23), supporting that RAF36 (WIRK1) plays a role in shoot gravitropism.

Further performing gravitropism assays on hypocotyls of 4-day-old dark-grown etiolated seedlings of both of *Col-0* and *wirk1-1* mutant plants every 12 h for a total of 36 h, we found that *wirk1-1* plants exhibited a slower bending speed as compared with that of *Col-0* seedlings (Fig. 7, *C* and *D* and supplemental Figs. S22*B* and S24). To confirm the function of RAF36 (WIRK1) in gravitropism, we also examined the curvature of etiolated seedlings of *raf36-2* (*wirk1-2*) after changing the gravity vector for 24 h, thus exhibiting a slower bending speed as compared to *Col-0* (supplemental Fig. S22*G*). This result supports that RAF36 (WIRK1) plays a role not only in the gravitropism of the inflorescence stem but also in that of the hypocotyl. However, *patl3* seedlings showed a similar hypocotyl gravitropic response to *Col-0* seedlings (supplemental Fig. S22, *C*–*F*), suggesting that PATL3 is not a critical component of the RAF36 (WIRK1)-mediated signaling pathway in response to the gravity vector changes.

## Discussion

### The Choice of TurboID (ID)-Based Proximity-Labeling (PL) Strategy

BioID, BioID2 (46) and APEX2 enzymes (42) are well-known biotin ligases that are capable of catalyzing the covalent conjugation of biotin (or called labeling) onto the putative interactors promiscuously. The TurboID (40) was, however, selected for this putative local interactomics study because it integrates the advantages of both BioID and APEX2 enzymes. TurboID was claimed to have a relatively rapid target protein-labeling rate (5–10 min) as compared to that of BioID ligase (46), and it is able to catalyze the biotin-labeling without a necessity of infiltration of substrates and reactive chemicals into cells during labeling in comparison with APEX2 (42). TurboID (or ID) is, in fact, capable of utilizing the cellular biotin to rapidly label the target proteins. As a result, TurboID biotin ligase (ID)-based proximity-labeling (PL) (39) has been widely applied in deciphering the putative local interactome of a bait fusion protein in mammalian cells, flies, worms (39) and various plants (*e.g.*, *Arabidopsis* and tobacco) (48, 52). Furthermore, to circumvent both problems of the wounding and the excess amount of biotin resulting from vacuum infiltration of plant cells (49), the *in planta* TurboID-based biotin-labeling inevitably became the PL method of our choice, in which *Arabidopsis* were cultivated on a special growth medium supplemented with exogenous biotin (47, 48, 52, 121, 122). It is believed that *Arabidopsis* takes up biotin through its roots from the growth medium. The increase of the supplemented biotin level is hypothesized to increase the biotin availability to biotin ligase in plant cells, thus leading to an increased biotin labeling of cellular proteins catalyzed by both recombinant TurboID biotin ligase and other endogenous biotin ligases (47, 48, 52). It was interesting to find that the higher concentrations of biotin used in plant cultivation result neither in toxicity to *Arabidopsis* nor abnormal development nor insensitive mechano-response. Presumably, the higher concentration of cellular biotin also did not affect the interaction of ID biotin ligase, MKK1-ID, and MKK2-ID fusion proteins with their respective target proteins. The uniqueness of *in planta* ID-mediated biotin-labeling is therefore suitable for collecting tissues from a short-time wind-treated transgenic *Arabidopsis*, *ID, MKK1-ID, and MKK2-ID*, where aerial plants were harvested and frozen immediately by liquid nitrogen following 5 min of gentle breeze treatment (Video Clip 1). The *in planta* PL method avoids the interference of infiltration force to the wind drag force loading.

As compared to those biotinylation sites catalyzed by plant endogenous enzymes, it was also interesting to find that the ID biotin ligase of bacterium origin as well as the recombinant fusion biotin ligase preferentially label the nearby target proteins at their loop regions (Fig. 4*H*). These experiments demonstrated that the prokaryotic ID biotin ligase has a quite different catalytic specificity from that of the endogenous biotin transferases of plant origin.

External biotin supplement to the growth medium dramatically increases the biotin modification (a type of post-translational modification, PTM) events on proteins in plant cells, which implies that a simple counting of biotinylation of target proteins discovered from biotinylproteomics cannot suggest its proximity to the bait ID fusion protein. Thus, both quantitative biotinylproteomics and bioinformatic analysis are required to determine the proximal proteins of the bait fusion protein.

### SILIA- and iBAQ-Based Quantitative Biotinylproteomics

The extracted ion chromatograph (XIC)-based peptide quantification has been widely applied in the quantitative PTM proteomics field in past decades as shown in SILAC approach (123). Especially, when it is in combination with the ^15^N-isotope metabolic labeling (59), the XIC-based quantitative PTM proteomics has clearly demonstrated its advantage in the ease of isotope labeling of the total cellular protein in the whole plant as demonstrated in both the Stable Isotope Labeling in Plant (SILIP) (124) and the ^15^N SILIA (59). The heavy nitrogen-coded salts can be supplemented together with the substrate biotin and transgene expression inducers (such as estradiol in this case) on solid medium to support normal growth and mechano-responsive properties of transgenic *Arabidopsis* and, at the same time, to regulate the bacterial biotin ligase fusion protein expression. The other conspicuous advantage of isotope labeling-based quantitative PTM proteomics is that the mixing of the total peptides or the total proteins of two plants before the affinity purification (AP) of PTM peptides, which is able to eliminate the experimental errors introduced by chromatographic affinity enrichment and separation processes. This type of quantitative PTM proteomics has been applied successfully in the finding of a group of 40-s touch-regulated phosphoproteins and multiple ethylene-signaling pathways in *Arabidopsis* (21, 57). Thus, in this study, we integrated the SILIA-based quantitative PTM proteomic protocol with the ID-based proximity-labeling in biotinylprotein quantitation to uncover the putative interactors specific to MKK1 and/or MKK2 (Figs. 1*A*, 4*B* and supplemental Fig. S2).

On the other hand, since the label-free quantification (LFQ, which frequently uses the MS1 precursor ion intensity to quantify peptides and proteins) is a relatively user-friendly quantitative proteomics approach (47, 49, 51, 125) and the MS1 precursor ion intensity-Based Absolute Quantitation (iBAQ) is able to include more quantifiable and unique (or non-redundant) peptide and biotinylpeptides (or PSM ≥1) into the identification of the proximal biotinylproteins of bait proteins (39, 48, 52, 126), we therefore selected iBAQ to perform the quantitative biotinylproteomics in search for the putative MKK1 and MKK2 interactors (Fig. 3, *A* and *B*). To compare the results of iBAQ method with that of SILIA-based quantitative biotinylproteomics, three genotypes of transgenic plants, *ID, MKK1-ID*, and *MKK2-ID*, were also labeled with both light (^14^N) and heavy (^15^N) nitrogen separately (supplemental Table S1). The MS1 precursor ion intensities from both types (light and heavy) of isotope-coded peptides derived from single biological replicate tissue were combined together to calculate the log2-ratios between MKK1-ID and ID as well as between MKK2-ID and ID samples using an in-house modified iBAQ quantification method (supplemental Tables S1 and S4*A*). As a result, the iBAQ quantitation method was able to identify an equal or a greater number of MKK1- and MKK2-specific interactors (Fig. 4, *A* and *B* and supplemental Fig. S15*A*), suggesting that both quantitative PL proteomics approaches are useful in the identification of putative interactors of a bait protein of interest.

The quantification procedures used by iBAQ (54) and SILIA-based quantification method (55) were quite different especially in the use of MS data. There were 11.7% (4020 out of 34,503; supplemental Tables S1, *G–R* and S5*A*) and 13.6% (5676 out of 41,877; supplemental Tables S2 and S5*C*) quantifiable peptides were included in iBAQ (54) and SILIA-based quantification method, respectively. By combining these two quantification methods in this experiment, both the accuracy and the sensitivity of quantification can be improved as compared to SILIA-based quantification alone. Regarding accuracy, XIC-based quantitation can be influenced by co-eluting peptides (127, 128), while MS1 precursor ion-based quantitation is less susceptible to such interference, as it is associated with identified PSM. However, MS1 precursor ion-based quantitation may be affected by dynamic exclusion, which temporarily places a mass into an exclusion list for a selected duration of time, thereby reducing the likelihood of the MS1 precursor ion being subjected to MS2 analysis at its highest intensity (129, 130). Therefore, the combination of both methods improves the accuracy of the experiment. In terms of sensitivity, it was interesting to find that iBAQ method had produced more significant results. A further examination of MS data of experiments E1 and E2 showed that in the experiment E1, *MKK1-ID* and *MKK2-ID* transgenic plants generated 745 quantifiable biotinylpeptides (18.5% of the total quantifiable peptides; supplemental Table S5*A*) while in the experiment E2, these two genotypes of plants produced 317 quantifiable biotinylpeptides (5.6% of the total quantifiable peptides; supplemental Table S5*C*). Thus, more biotinylpeptides used in quantitation lead to more quantification results for biotinylproteins. Second, the quantifiable peptides in E1 contained more missing values as compared to that in E2. In summary, in E1, 2187 quantifiable peptides (54.4% of the total quantifiable peptides; supplemental Table S5*A*) were derived from only one transgenic plant (either MKK1-ID or MKK2-ID) while 1030 quantifiable peptides (18.1% of the total quantifiable peptides; supplemental Table S5*C*) were derived from only one transgenic plant in E2. As we have used half of the lowest MS intensity of each MS data to represent the missing value, the differences in the iBAQ quantitation of MKK1-ID and MKK2-ID samples in E1 are likely to increase more findings of significantly labeled biotinylproteins (or putative interactors) as compared to that of the SILIA-based method in E2.

Why did we have to use the ID biotin ligase as a universal control for an ID fusion protein like MKK1/2-ID during quantitative PTM proteomics? It has been reported that the higher activity and the rapid proximity-labeling (PL) using TurboID (ID) may result in non-specific labeling due to the labeling distance of TurboID (39, 131). A properly chosen control transgenic plant expressing *ID* gene should be critical for distinguishing the true proximal candidate proteins from those nonspecifically bound to beads or those randomly labeled by ID fusion protein (47). As MKK1 and MKK2 are two kinase components of the canonical MAPK cascade localized in the cytoplasm and ID biotin ligase is also a cytosolic protein in transgenic plant cells, the *ID* transgenic plant is therefore considered as an appropriate control to remove the non-specific biotinylprotein background during pairwise quantification (132). In addition, the use of estradiol-induction in transgenic plants can control the expression levels of both the bait ID-fusion protein expression plants and *ID* plants (89). Further normalization of MS data against either the ID protein or the ID fusion protein expression level in a transgenic plant can reduce the interference, resulting from the different levels of TurboID biotin ligase activities, on the significant quantification of interactors or specific interactors of a bait protein.

Both transgenic MKK1-ID and MKK2-ID fusion proteins were found to be overly biotinylated as compared to the zero biotinylation event on the ID biotin ligase (Fig. 2, *I* and *J* and supplemental Table S3*C*). The phenomena are the best manifestations that the biotin ligase indeed biotinylates any proximal proteins or protein domains regardless of this polypeptide being physically linked or disconnected from the ID biotin ligase as long as the substrate polypeptide locates nearby the biotin ligase. Thus, the more biotinylsites found from a protein, the more proximal it is to the bait fusion protein when *ID* transgenic plant biotinylproteins were compared with those of bait fusion protein-expressing plants (given the percentage of lysine residues on this biotinylpolypeptide unchanged). We therefore proposed to calculate the biotin occupancy ratio for each biotinylprotein identified from *ID* and those bait fusion protein plants (Fig. 3, *G*–*J*). The volcano plots of BOR calculated from biotinylproteins of two different transgenic plants provided an alternative parameter to determine the specificity and the proximity of an interactor to a bait protein of choice.

### Modeling of Signal Transduction Pathways for Plant Wind Mechano-Response and Gravitropism

The wind force loading inflicted on plants is able to press the plant aerial organs to sway back and forth. Under the influences of wind tensile and compressive forces, plants undergo thigmomorphogenesis (13, 133). At the same, the passive movement of plants may cause the involuntarily tilting of organs, or called reorientation of plants (134). Consequently, the reorientation of plant organs against the gravity vector above a certain angle is able to trigger gravity force signaling (135). The negative anemotropism is the very term used to describe such reorientation of plants, in which the inflorescence stem orients itself toward the direction of wind under the constant and unidirectional wind treatment (16). However, if younger *Arabidopsis* plants were observed in a wind tunnel, the primary growth region bend themselves toward the source of wind probably due to the influence of gravitational force (16). This wind-induced directional growth is defined as positive anemotropism or positive thigmotropism. Thus, it is likely that both wind mechanical perturbation (wind drag force) and gravistimulation force signals may be simultaneously transduced and integrated intracellularly and extracellularly among cells of wind-evoked aerial organs of a plant to trigger both thigmomorphogeneiss and thigmotropism (including anemotropism). The relevant question is how plant cells sense, transduce and integrate these two classes of mechanical signals during the wind mechano-response regardless of the dry effects brought about by transpiration, temperature and gas exchange resulted from the wind-blowing (136).

**Fig. 8:**
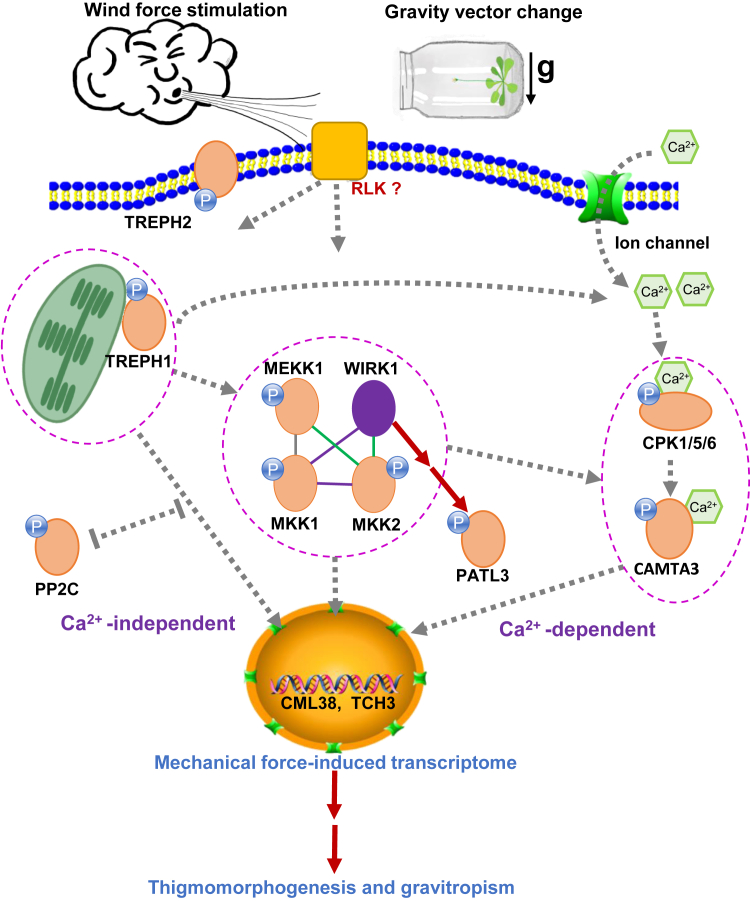
**A model of WIRK1 (RAF36)-mediated thigmomorphogenesis and gravitropism.** The model of the mechanical force for wind drag stimulation and gravity vector change was built based on the information of biotinylproteomics analysis, bioinformatic analysis, function confirmation from this study, and reanalyzed touch data. WIRK1 (RAF36) and MEKK1 transduce mechanical signals through MKK1/MKK2 to regulate the expression of CML38 and TCH3, in addition, PATL3 is also a downstream component of WIRK1 (RAF36)-regulated wind resistance. Phosphoproteins quantified from reanalyzed 40-s touch data are annotated by *orange ovals* (supplemental Table S8), and the *blue* P in the *circle* represents the phosphorylation site. *Purple oval* represents WIRK1 (RAF36) and P in *blue purple circle* represents phosphorylation against 40s-touch stimulation. The *yellow square* represents mechanosignaling receptors, a question mark followed with RLKs represents this receptor could be receptor-like kinase, and the *green* channel is representative of the calcium channel. Ca^2+^ in *green* hexagon represents calcium ion. *Green solid lines*, common PPIs between this study and database including STRING and BioGRID; *purple solid lines*, new PPIs in this study; *grey solid lines*, PPIs in databases; *solid double red lines with arrows*, verified signal transduction pathway in this manuscript; *dashed grey line with arrows*, direction of signal transduction; *horizontal line* at the ends of the dashed line, inhibitory effect; *dashed purple circle*, putative signal cluster.

Unravelling of the mechano-sensors and early signaling components of plant mechano-responses has been a daunting task. According to the current state of knowledge, two classes of mechano-sensory apparatuses have been hypothesized to mediate a network of force signaling in thigmomorphogenesis and thigmotropism. The first class would be mechanosensitive divalent and monovalent ion channels including Mechanosensitive channels of Small conductance Like (MSL), Mid1-Complementing Activity (MCA), hyperosmolality-gated calcium-permeable channels (OSCA), Piezo one and Two-Pore K^+^ channel (TPK) (137, 138) while the other class would be membrane-bound receptor-like kinases (RLKs) (139). Upon a wind or touch mechanical perturbation, a single cytoplasmic calcium spike [Ca^2+^] _cyt_ is normally observed within seconds to minutes (24, 140). Consequently, these cytoplasmic calcium ions may interact with calcium-dependent protein kinases (CDPKs or CPKs), calcium-binding proteins (CBPs or calmodulins) and calmodulin-dependent protein kinases (CAMKs) to activate the phosphorylation of downstream proteins such as transcription factors and kinases (141). Alternatively, those membrane-bound putative receptor-like kinases, such as the wall-associated kinases (WAK), may function as mechanosensors to phosphorylate downstream proteins and deliver the force signals directly down to nuclear events (25). Indeed, the recent quantitative phosphoproteomics found that a module of protein phosphorylation-related enzymes, including MAPK kinases, calcium-dependent kinase, calmodulin-dependent protein kinases and PP2C phosphatases, were rapidly phosphorylated within 40 s of mechanical perturbation (21). The phosphorylation of both MKK1 and MKK2 was upregulated upon water sprinkling, wind, and touch treatment (21). It is therefore conceivable to hypothesize that both calcium-dependent and -independent phosphor-relays might crosstalk during mechanotransduction. As to the molecular mechanism of gravisensing, the current knowledge is quite limited. Out of three hypotheses proposed for gravisensing, *i.e.*, starch-statolith theory (142), gravitational pressure theory (143) and tensegrity theory (144), the current popular molecular and cellular model for gravisensing (18) involves both the amyloplast (one type of statolith) movement (145) and a cytoskeleton-plasma membrane-cell wall (CPMCW) continuum (146). TREPH1, which is a touch-regulated cytoskeletal protein and a member of chloroplast (or statolith) movement-related phosphoproteins, might be the integral component of this CPMCW continuum to regulate the touch response (Fig. 8) (21). Upon the initial gravistimulation accompanied by the moving organelles and the unbalanced cellular architecture, the gravitational force signals are subsequently and hypothetically transduced through both the secondary messenger, a biphasic calcium transient (147), and phosphor-relays (70). How these versatile mechanical signals, wind force, and gravity force, would be converted into multiple calcium-dependent and -independent phosphor-relays and how the protein phosphorylation cascades would be integrated together and decoded into diverse gene expression and plant mechano-response became interesting and puzzling questions.

In our effort to identify a novel component of force signaling, a Raf-like kinase 36 (WIRK1) was indeed found to serve as a novel interactor of both mechano-responsive MKK1 and MKK2 (Figs. 3 and 4), and especially, it played a role in both thigmomorphogenesis and gravitropism (Figs. 6 and 7). This data, for the first time, suggested to us that both wind force-induced phosphor-relay may indeed integrate together with that of gravistimulation signaling at the Raf-like kinase family level. Given that Raf-like kinase 36 (or now called WIRK1) functions upstream of MKK2 as in signaling of plant response to pathogen (148), and that MKK1 is a substrate of MEKK1 in regulating plant innate immunity (27, 115), we performed bioinformatic modularization analysis of putative interactors of MKK1 and MKK2 kinases identified by the quantitative PL proteomics (Fig. 5*A*) and proposed a module of core WIRK1/MEKK1-MKK1/2 phosphor-relay cascade (Fig. 5*B*). This core mechanical phosphor-relay module may receive the phosphorylation activation signals from a few upstream activities of chloroplast movement (related to TREPH1), RLKs or CPKs or other ion channels to further deliver the phosphor-relays to downstream either MAPKs or transcription factors like the phosphorylated CAMTA3 and PATL3 (supplemental Table S8), leading to gene expression (TCH3 and CML38) (21) and mechano-responses and gravicurvature (Fig. 8). The newly proposed mechanical signaling model is analogous to that of phosphor-relay mediated cold-stress cell signaling model, where cold stress activates CRLK1, which increases phosphorylation of both MEKK1 and MKK2, consequently leading to activation of MAPK4/6 (149, 150). Experiments might be performed in the future to examine if *wirk1* mutant would affect the development of touch-induced thigmomorphogenesis and positive anemotropic response in *Arabidopsis*.

### Phytohormones Probably Play a Role Downstream of WIRK1 in Mechanical Signaling and Thigmomorphogenesis

Phytohormones interact extensively to regulate mechanical responses. Ethylene and jasmonic acid (JA) are two hormones known to be involved in signaling cascades under mechanical stress stimulation (151). In *Arabidopsis*, TCH1-4 mRNA expression levels were induced by mechanical stress, including touch, wound, and wind stimulation (19). Among these TCH genes, TCH3 also showed an increase after 1 h of exposure to 100 ppm ethylene, although the induced response was delayed and weaker than that induced by touch (19). Using *ein6* mutants in *Arabidopsis*, the function of the EIN6 protein required for TCH3 expression after mechanical stimulation was confirmed (152). Besides, researchers compared the evolution of ethylene in two ecotypes of *Stellaria longipes* (alpine and grassland) and found that alpine plants reflect an increased ethylene evolution in response to wind stimulation (153). ACS6, which is a 1-aminocyclopropane-1-carboxylic acid (ACC) synthase 6, was induced by touch and reached maximal transcription at 15 min in *Arabidopsis* (154). Meanwhile, the ACC increased sharply between 15 and 30 min after touch stimulation. These findings supported that touch affects ethylene production and that the presence of high concentrations of ethylene also affects touch signals. However, in *A. thaliana*, the ethylene-insensitive mutants *etr1-3* and *ein2-1* responded to wind similarly to what wild type did, in terms of the delayed flowering, indicating that ethylene is not required for *Arabidopsis* response to mechanical stimuli (155). Furthermore, in *Nicotianum tabacum*, the flexed ethylene-insensitive transgenic (Tetr) plants produced shorter, thicker stems than those non-flexed ones, similar to that of the wild type (156). On the other hand, the results showed that although flexure reduced the growth of wild-type shoots, it did not affect the growth of Tetr plants. In addition, the expression of EIN3, which is a transcription factor, was increased after touch stimulation, and biochemical analysis indicated that EIN3 binds to the promoter of GA2ox8 (encoding gibberellin two-oxidase 8) and represses GA2ox8 transcription (157). The interaction between EIN3 and the JA-activated transcription factor MYC2 has been confirmed and mutually inhibits each other’s transcriptional activity, regulating the antagonistic effects of JA and ET in different pathways, such as wound-responsive gene expression (158). Furthermore, EIN3 has been reported to be directly associated with the PGX3 (POLYGALACTURONASE INVOLVED IN EXPANSION3) promoter and is required for touch repressed PGX3 expression (159). These results indicated that ethylene might be important for mechanotransduction. However, the precise function and the signaling of ethylene in thigmomorphogenesis following mechanical stimulation are not clear. In the case of jasmonic acid (JA), many reports supported that it plays a crucial role in mechanical stress and thigmomorphogenesis. In *Arabidopsis*, the expression *OPR3* gene, which encodes a 12-oxophytodienoate reductase that is required for jasmonate biosynthesis, was induced by different stresses such as touch, wind and wounding in wildtype (160). In addition, OPR3 transcription was also induced in an ethylene-insensitive mutant (*etr1-3*) under touch stimulation (161). The JA was shown to be required for and to promote the unique features of *Arabidopsis* thigmomorphogenesis using various JA-related mutants (162). It has been reported that MYC2 functions together with MYC3 and MYC4 to regulate wound-induced JA accumulation through binding to the promoters of genes of JA biosynthetic and catabolic functions, thereby promoting their transcription (163). The latest paper confirms that MYC2 directly promotes the expression of GA2ox7 (GIBBERELLIN 2-OXIDASE 7) gene by binding to the G-box motif wihtin the GA2ox7 promoter (157). It also mentioned that through genetic analysis, the ethylene and JA pathways appear to independently control the expression of GA2ox8 and GA2ox7 genes, respectively. Mechanical stress induces the expression of ET, JA, and GA-related genes, indicating the convergence of multiple phytohormone signaling pathways. These phytohormones may interact extensively in plant to regulate thigmomorphogenesis (151).

To address if ethylene participates in the regulation of wind-induced WIRK1-PATL3 signaling pathway, we used an octuple (eight ACC synthase genes) *acs*-deficient line, whose ACC production was severely hindered, resulting in a decrease in ethylene production (91, 164) (supplemental Fig. S25). Firstly, *wirk1-1* showed a similar triple response to that of *Col-0* upon ethylene or ACC treatment (supplemental Fig. S25*A*). After 5 min of wind stimulation, we can see a regulatory pattern similar to that of the wild type in the *acs octuple* mutant plants (supplemental Fig. S25*B*), and the low expression of EIN3 in the *acs octuple* mutant indicates reduced ethylene in plants (supplemental Fig. S25*C*), which supports wind-induced WIRK1-PATL3 signaling pathway is independent of changes in plant ethylene concentration.

Furthermore, we treated plants of different genotypes with exogenous ethylene treatment (5 ppm). We found that ethylene treatment for 5 min did not change the phosphorylation of PATL3 in all three genotypes (supplemental Fig. S25*D*), but after long-term stimulation, the phosphorylation level of PATL3 decreased. We are unable to determine if ethylene stimulation leads to PATL3 degradation. Therefore, mechanical signaling is independent of ethylene signaling in the WIRK1-PATL3 signaling pathway at least within a short period of force laoding time. To discover the specific roles of ethylene and other hormones in thigmomorphogenesis, hormone synthesis mutants may be more useful than those hormone-sensing mutants assuming that some components of hormone signaling might participate in force signaling. For example, we have performed a touch response assay on this octuple *acs*-deficient mutant line. The preliminary data indicated that the ethylene biosynthesis-deficient mutant exhibited a normal touch response (data not shown here), suggesting that ethylene may not play a critical role in *Arabidopsis* thigomomorphogenesis.

## Data Availability

All MS data have been deposited at the ProteomeXchange Consortium *via* PRIDE with the table identifier PXD048765 (**Username:** reviewer_pxd048765@ebi.ac.uk; **Password:** rnbaZmCT). The MS data have also been deposited at ScienceDB with the https://doi.org/10.57760/sciencedb.09578 **(Private link for reviewer** https://www.scidb.cn/s/juE7Vj).

## Supplemental data

This article contains supplemental data (54, 55).

## Conflict of interest

The authors declare that they have no known competing financial interests or personal relationships that could have appeared to influence the work reported in this paper.

## Supplemental Data

Supplemental Table S10

Video Clip 1
