# Supplementary material for: The Quantitative Biotinylproteomics Studies Reveal a WInd-Related Kinase 1 (Raf-Like Kinase 36) Functioning as an Early Signaling Component in Wind-Induced Thigmomorphogenesis and Gravitropism
Source: Mol Cell Proteomics. 2024 Feb 15;23(3):100738. doi: 10.1016/j.mcpro.2024.100738 (PMC10951710; doi:10.1016/j.mcpro.2024.100738)
Supplement: Supplemental Figures S1–S25 [file mmc12.pdf]

Supplemental FIG. S1

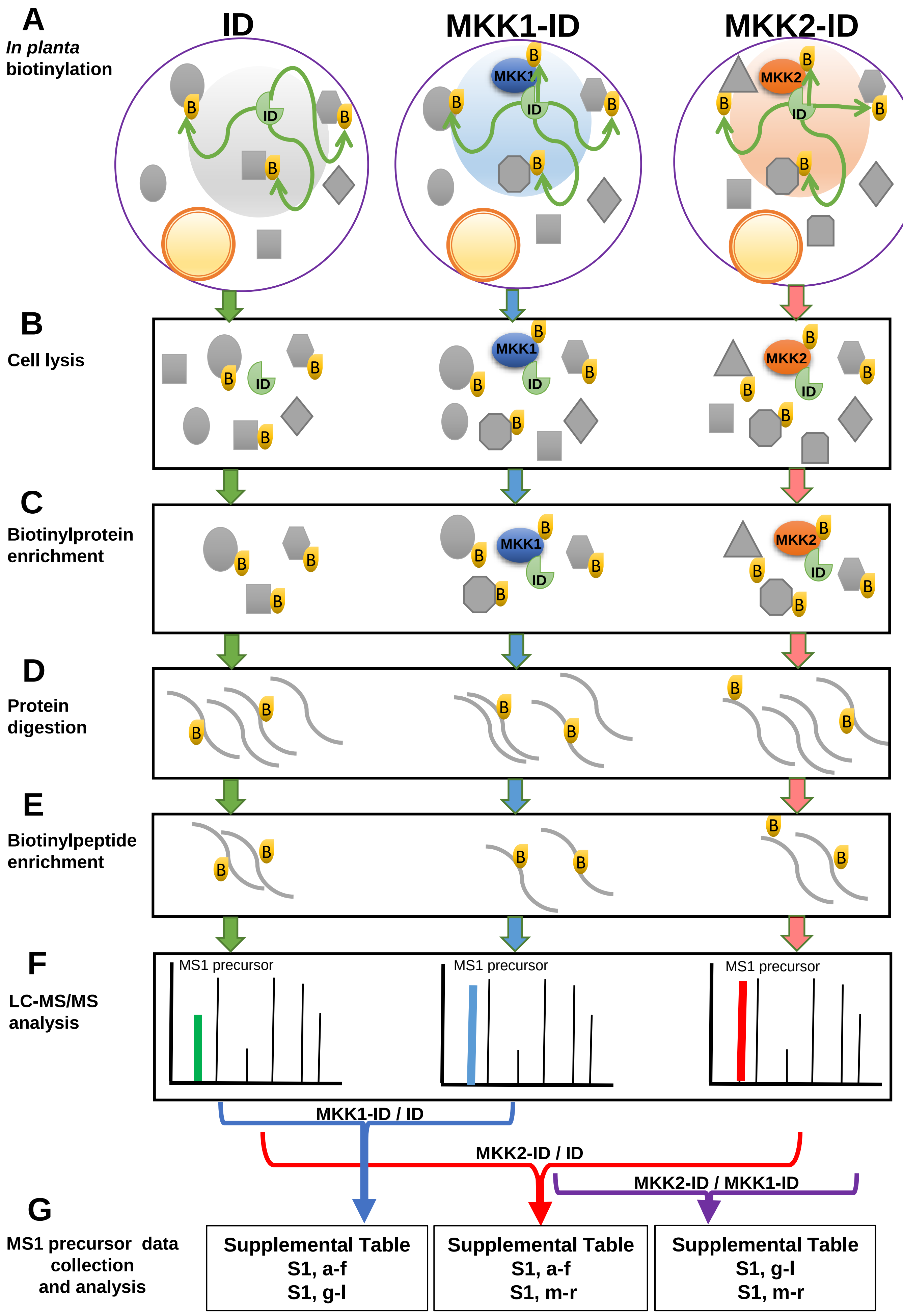

Supplemental FIG. S1. Overview of the TurboID-based Proximity Labeling followed by intensity-based absolute quantification (iBAQ). *A*, turboID–based proximity labeling happened in *ID*, *MKK1-ID* and *MKK2-ID* Arabidopsis adult plants under wind treatment when biotin was added into the plant growth medium at the beginning of medium preparation and then the treated tissue were subsequently collected with liquid nitrogen. ID in green sector, TurboID; grey colored oval, square, rhombus, triangle, octagon, hexagon and irregular shapes, different proteins in cell; blue and orange ovals, MKK1 and MKK2; circle wrapped in orange thread, nuclear; B in drop shape, biotin. *B*, the total proteins were extracted with Urea-based protein extraction buffer (UEB) and biotinylated and normal proteins (without biotinylation) were lysed from cells. *C*, biotinylproteins were enriched with streptavidin agarose beads in denature buffer. D, biotinylproteins were digested into peptides with in-gel digestion method. Irregular lines represent peptides. *E*, with the help of streptavidin agarose beads, biotinylpeptides were enriched. *F*, the biotinylpeptides were analyzed by LC-MS/MS with three biological replicates per genotype. *G*, three sets of quantitation including MKK1-ID vs ID, MKK2-ID vs ID and MKK2-ID vs MKK1-ID were quantified by the iBAQ method. It is related to Figure 1-4 and Supplemental Table S1, S3, S4 and S5.

Supplemental FIG. S2

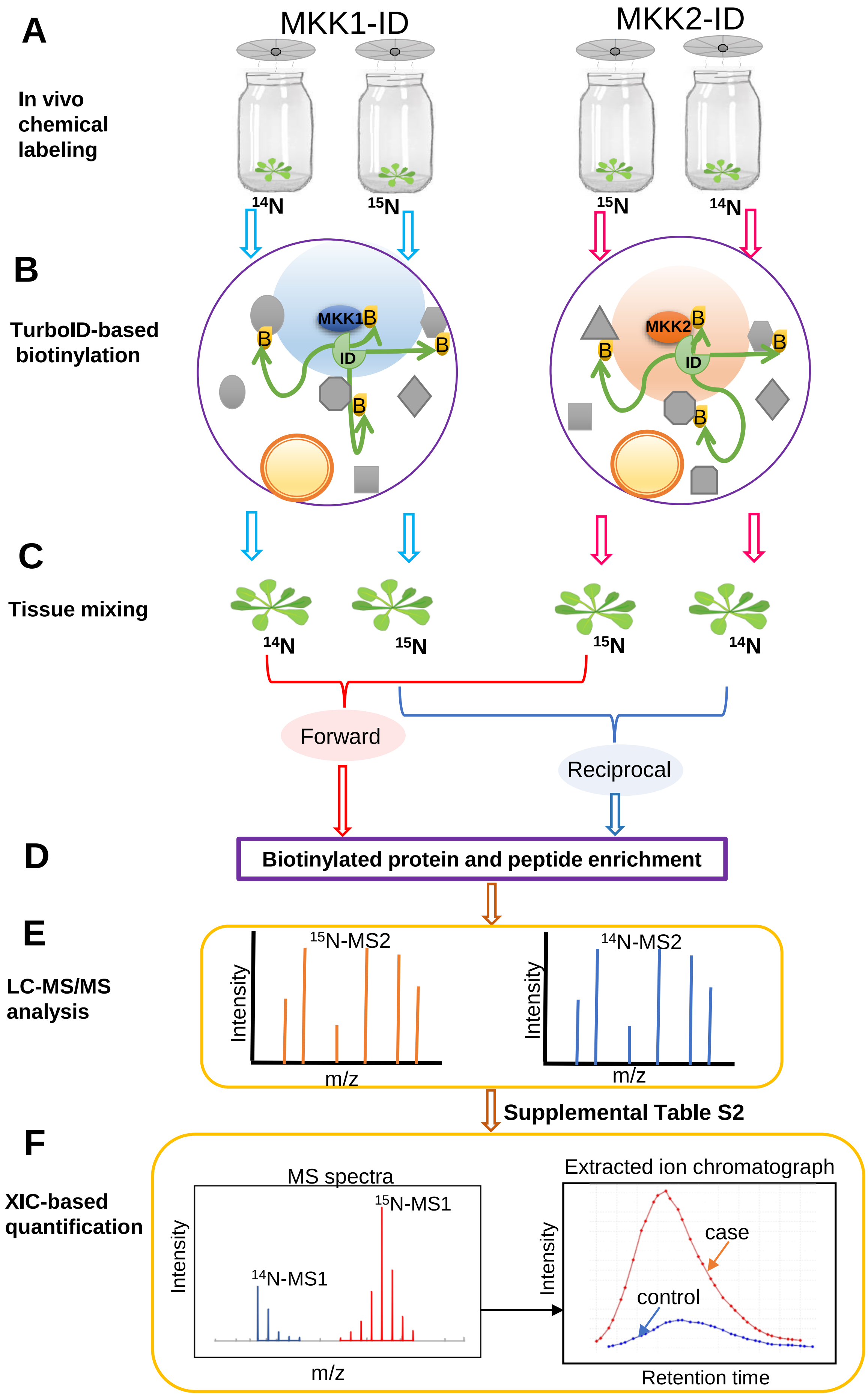

Supplemental FIG. S2. Overview of the TurboID-based Proximity Labeling followed by XIC-based quantification. *A*, half of *MKK1-ID* and *MKK2-ID* transgenics were grown and labeled with ^{14}N or ^{15}N stable isotope-coded salt, respectively. *B*, both MKK1-ID-based and MKK2-ID-based proximity labeling occurred with biotin in the medium. ID in green sector, TurboID; grey colored oval, square, rhombus, triangle, octagon, hexagon and irregular shapes, different proteins in cell; blue and orange ovals, MKK1 and MKK2; circle wrapped in orange thread, nuclear; B in drop shape, biotin. *C*, after processing, the tissue was collected with liquid nitrogen and ground to a fine powder, then mixed into Forward (F) and Reciprocal (R) groups. In Forward (F) mixing group, ^{14}N labeled MKK1-ID tissue and ^{15}N labeled MKK2-ID tissue were mixed while in Reciprocal (R) mixing group, ^{15}N labeled MKK1-ID tissue and ^{14}N labeled MKK2-ID tissue were mixed. *D*, the procedures of biotinylprotein and biotinylpeptide enrichment were the same as supplemental Fig. S1, *B*-*E*. *E*, the biotinylpeptides were analyzed by LC-MS/MS. *F*, the biotinylrotein quantitation was conducted based on the extracted ion chromatograms (XIC) from MS1 by an in-house program SQUA. It is related to Figure 1, 2 and 4 and Supplemental Table S2, S3 and S5.

Supplemental FIG. S3

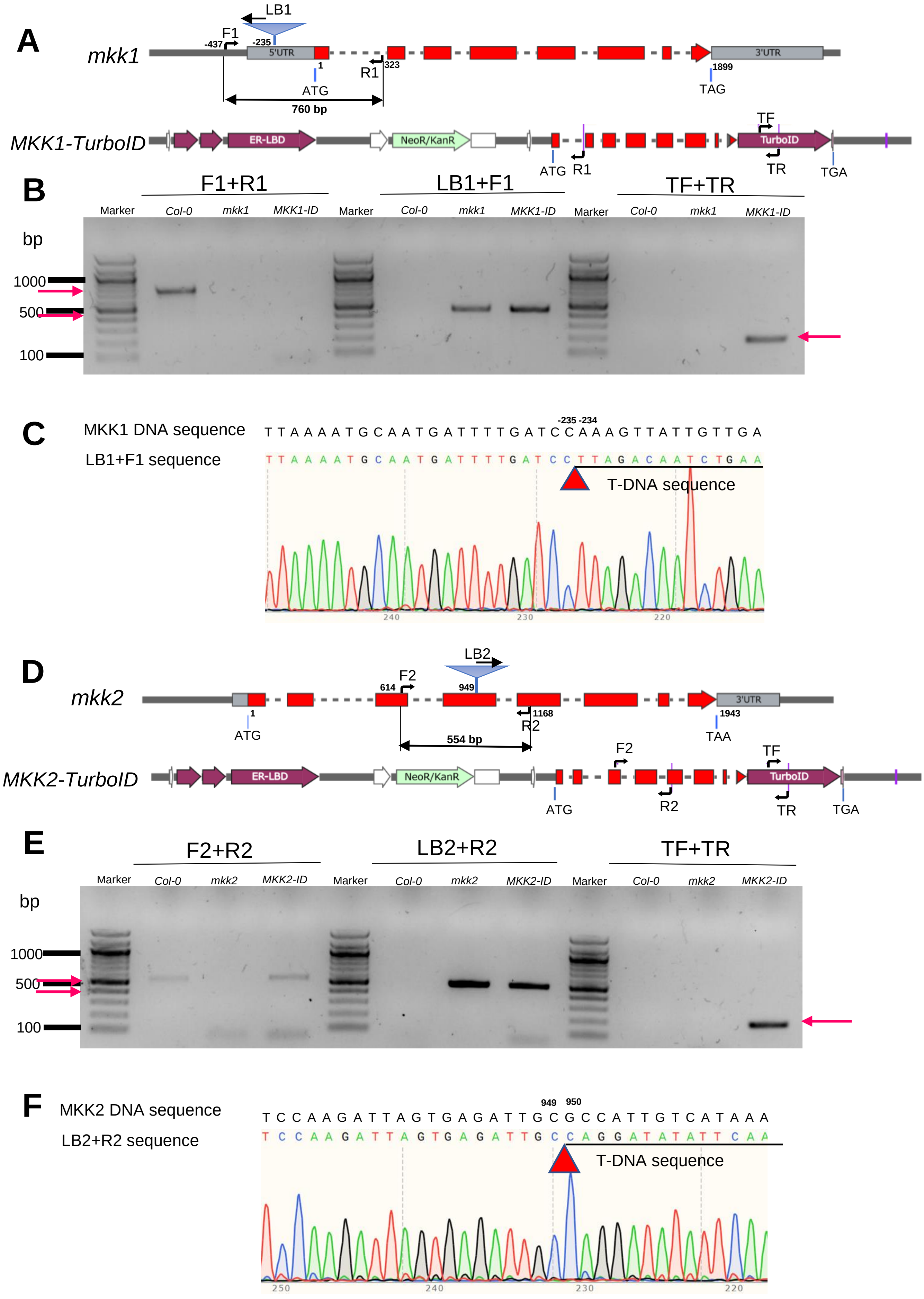

Supplemental FIG. S3. Genotyping of T-DNA insertion mutants and transgenic lines. *A* and *D*, DNAs schematic representation of *mkk1 or mkk2* mutant and *MKK1-ID* or *MKK2-ID* constructs. Triangles represent T-DNA insertion sites in T-DNA insertional *mkk1* or *mkk2* mutants. Primers used to genotype are presented with arrows. Grey boxes indicate as 5' UTR or 3'UTR. Red boxes are exons of MKK1 or MKK2 including dotted intron. ER-LBD which produces estrogen nuclear receptor alpha ligand binding domain, kanamycin resistant gene and TurboID are showed in purple and green color. ATG and TAA/TGA/TAG represent the start and stop codon, respectively. *B*, genotyping of *mkk1* T-DNA insertions and *MKK1-ID* transgenic plants by PCR. Primer pairs used to verify *mkk1* mutant were F1+R1 and LB1+F1. The size of F1+R1 is 760 bp. *MKK1-ID* transformation was verified by the TF+TR, F1+R1 and LB1+F1. *C*, T-DNA insertion site verification of *mkk1* by DNA sequencing. The insertion site is at 5'UTR of *MKK1* gene. *E*, genotyping of *mkk2* T-DNA insertions and *MKK2-ID* transgenic plants by PCR. Primer pairs used to verify *mkk2* mutant were F2+R2 and LB2+R2. The size of F2+R2 is 554 bp. *MKK2-ID* transformation was verified by the TF+TR, F2+R2 and LB2+R2. *F*, T-DNA insertion site verification of *mkk2* by DNA sequencing. The insertion site is at 946bp of *MKK2* gene. All primers are summarized in Supplemental Table S10. It is related to Figure 1.

Supplemental FIG. S4

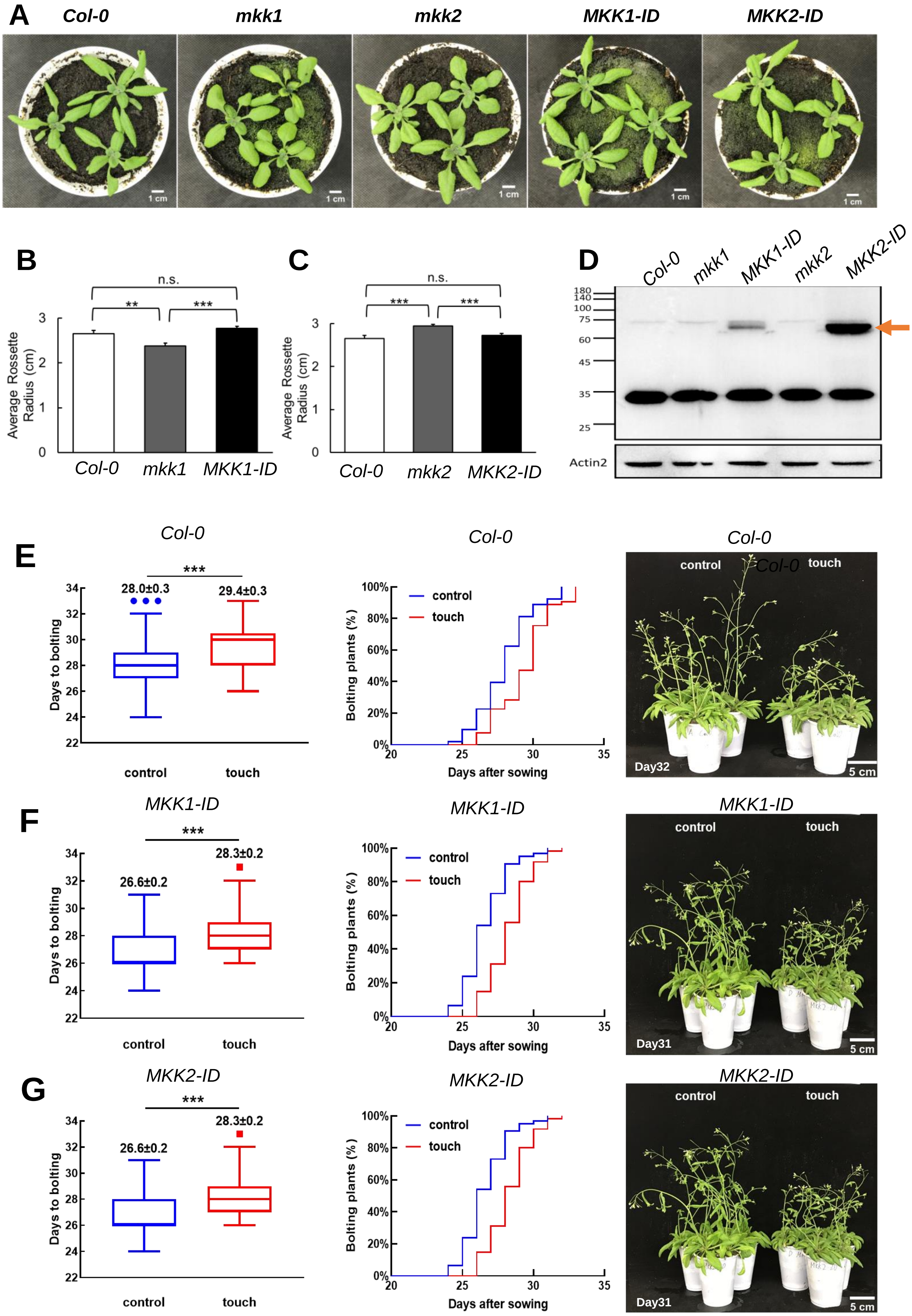

Supplemental FIG. S4. Phenotype and restoration of gene function of the transgenics. *A*, whole-plant phenotype of Arabidopsis *Col-0*, *mkk1*, *mkk2*, *MKK1-ID*, *MKK2-ID*. *B* and *C*, radius of Arabidopsis rosettes. 16-day-old plants grown in soil were used to measure the plant size. *D*, biotinylation level of Arabidopsis *Col-0*, *mkk1*, *mkk2*, *MKK1-ID*, *MKK2-ID*. The upper blot was incubated with anti-biotin and the lower blot was incubated with anti-actin. *E*, *F and G*, touch responses of *Col0, MKK1-ID, MKK2-ID.* Box and whisker plots of bolting days of individual plants and Kaplan Meier survival lines were used to compare the bolted plants over the days after sowing in untouched control and auto-machine touched *Col0*, *MKK1-ID* and *MKK2-ID* plants. The representative photos of untouched and touch-treated plants are in right panels. Values in *C*-*G* are means ± SEM ($n \geq 60$). Unpaired student's t test was applied: $^{**}P < 0.01$, $^{***}P < 0.001$ and n.s. (nonsignificant) $P \geq 0.05$. It is related to Figure 1.

Supplemental FIG. S5

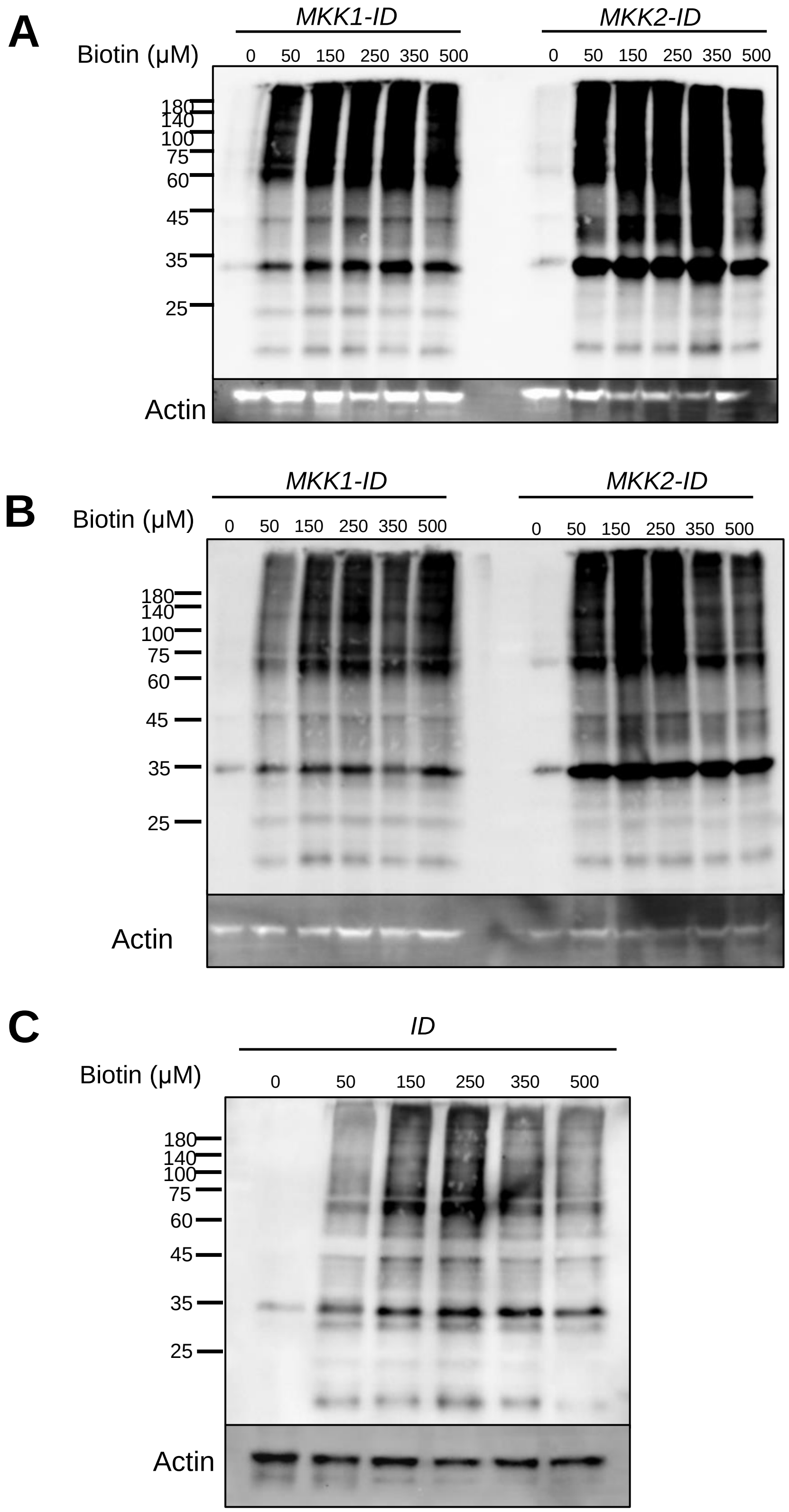

Supplemental FIG. S5. Immunoblot analysis of biotinylproteins using anti-biotin. *A* and *B*, two additional biological replicates of total western blots showing biotinylation levels with different concentrations of biotin in the growth medium. The left six lanes were the biotinylation results of *MKK1-ID*, while other right six lanes showed *MKK2-ID*'s results. *C*, one biological replicate of total western blots showing biotinylation levels by ID with the addition of different concentrations of biotin in the growth medium. It is related to Figure 1.

Supplemental FIG. S6

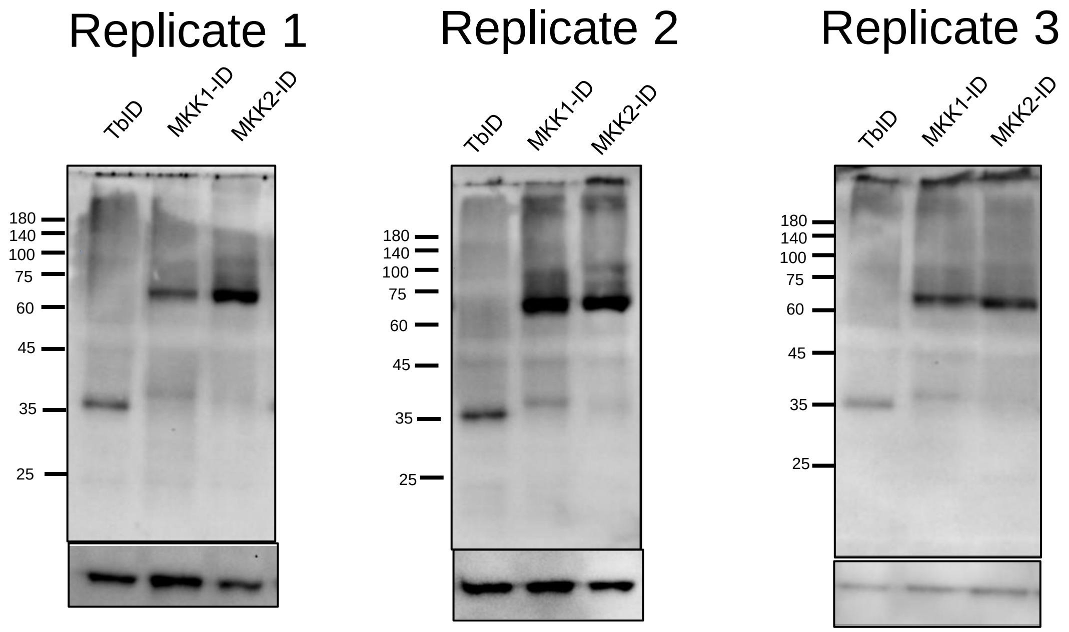

Supplemental FIG. S6. TurboID and fusion protein expression by immunoblots. Expression of fusion proteins from three biological replicates. Anti-TurboID was used to detect the expression level of TurboID or fusion protein in three types of transgenics. It is related to Figure 1.

Supplemental FIG. S7

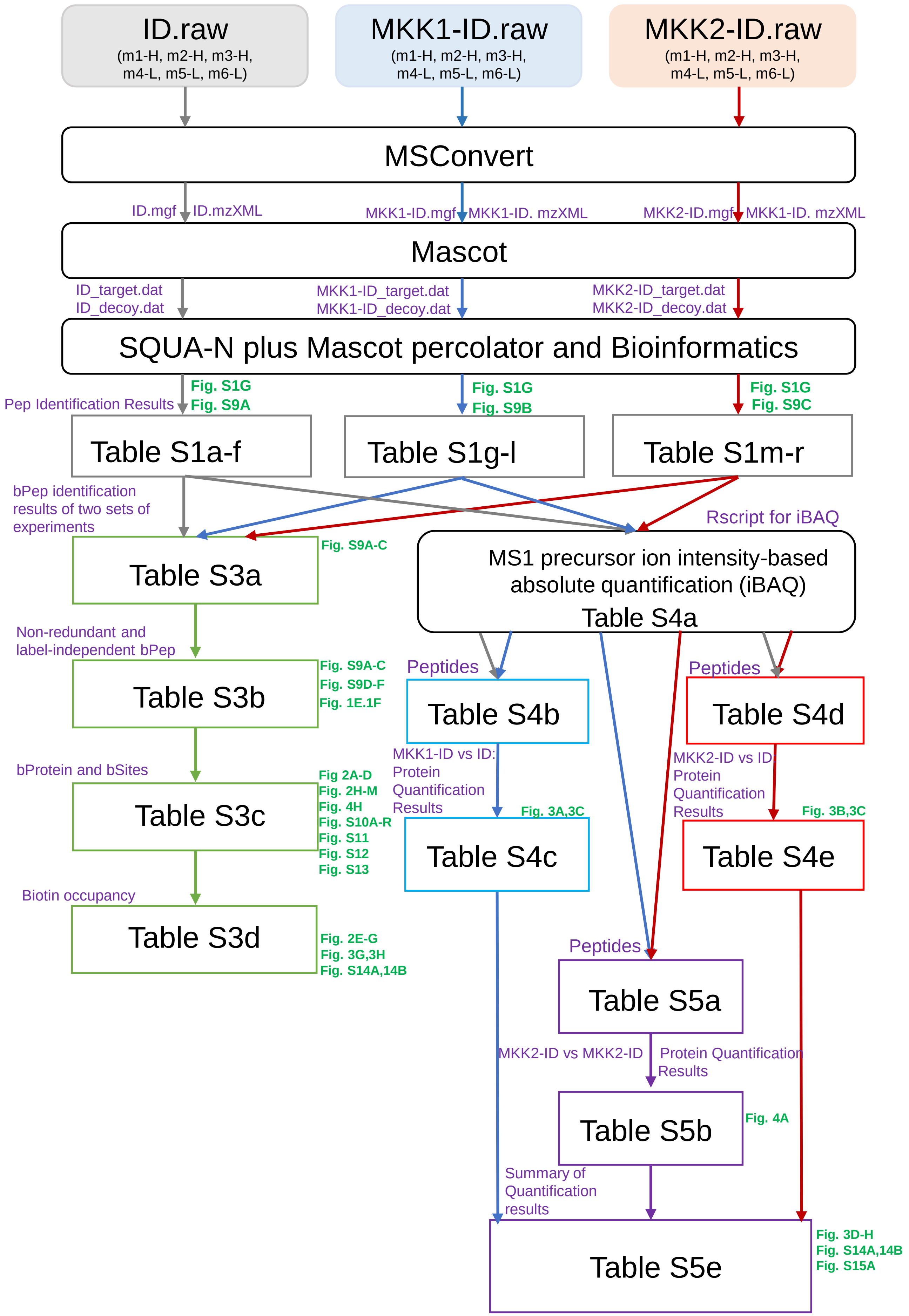

Supplemental FIG. S7. Pipeline of data analysis from MS1 precursor ion intensity-based quantification in Experiment 1 (E1).

The raw data of ID, MKK1-ID and MKK2-ID were converted to mzXML and mgf files by MSConverter, respectively. The mgf files of ID, MKK1-ID and MKK2-ID were searched against the Arabidopsis target and decoy TAIR10 database using the Mascot search engine. The output of the Mascot are pairs of target.dat and decoy.dat files. Paired dat files with corresponding mzXML files were used as input for Percolator (version 3.02) and SQUA-N (Stable isotope-based Quantitation, Version 2.0) to obtain identification results, and with MS1 precursor ion intensity-based absolute quantification (iBAQ) method to obtain the quantification results. The process of data processing and graph generation is shown in the figure: Supplemental Table S1 contains all the ambiguous, redundant, non-repeatable and label dependent peptides of FDR < 0.01 from ID, MKK1-ID and MKK2-ID in three biological replicates, respectively. Supplemental Table S3a contains unambiguous, redundant, non-repeatable and label-dependent biotinylpeptides from two sets of experiments including E1 and E2. Supplemental Table S3b contains unambiguous, non-redundant, repeatable and label-independent biotinylpeptides from two sets of experiments including E1 and E2. Supplementary Table S3c contains all the biotinylproteins from two sets of experiments. Supplemental Table S3d contains all biotinylproteins with biotin occupancy from two sets of experiments. Supplemental Table S4a contains the Rscript of IBAQ-based quantification. Supplemental Table S4b contains total peptides of MKK1-ID VS. ID in E1. Supplemental Table S4c contains IBAQ-based quantification results of MKK1-ID VS. ID in E1. Supplemental Table S4d contains total peptides of MKK2-ID VS. ID in E1. Supplemental Table S4e contains IBAQ-based quantification results of MKK2-ID VS. ID in E1. Supplemental Table S5a contains total peptides of MKK2-ID VS. MKK1-ID of IBAQ method in E1. Supplemental Table S5b contains IBAQ-based quantification results of MKK2-ID VS. MKK1-ID in E1. Supplemental Table S5c contains total peptides of MKK2-ID VS. MKK1-ID of MS1-based method in E2. Supplemental Table S5d contains MS1-based quantification results of MKK2-ID VS. MKK1-ID in E2. Supplemental Table S5e contains the summary of quantification results from Supplemental Table S4c, e and Supplemental Table S5b, d.

Supplemental Figure S1G was generated using Supplemental Table S1. Supplemental Figure S9A-C was generated using Supplemental Table S1, Supplemental Table S3a and Supplemental Table S3b. Supplemental Figure S9D-F and Figure 1E-F were generated using Supplemental Table S3b. Figure 2A-D, Figure 2H-M, Figure 4H, Supplemental Figure S10A-R and Supplemental Figure S11-13 were generated using Supplemental Table S3c. Figure 2E-G were generated using Supplemental Table S3d. Figure 3A,C and Figure 3B,C were generated using Supplemental Table S4c and S4e, respectively. Figure 4A was generated using Supplemental Table S5b. Figure 3D-F and Supplemental Figure S15A were generated using Supplemental Table S5e. Figure 3G-H and Supplemental Figure S14, A and B were generated using Supplemental Table S3d and Supplemental Table S5e.

It is related to Figure 1-5, Supplemental Figure S1, S2, S9-S15, and Supplemental Table S1, S3-S5.

Supplemental FIG. S8

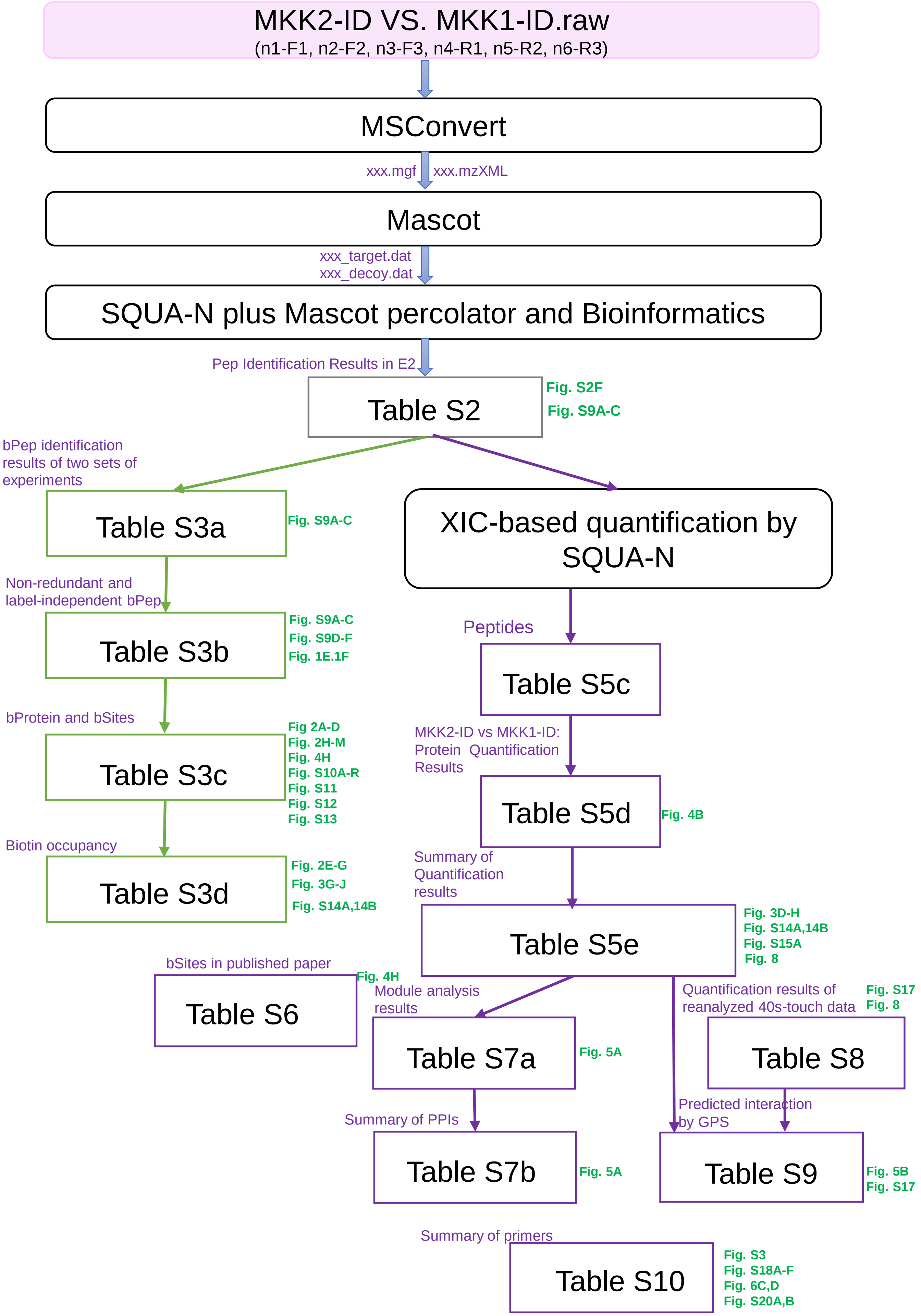

Supplemental FIG. S8. Pipeline of data analysis from XIC-based quantification

The raw data of MKK2-ID VS. MKK1-ID in Experiment 2 (E2) were converted to mzXML and mgf files by MSConverter. The mgf files were searched against the Arabidopsis target and decoy TAIR10 database using the Mascot search engine. The output of the Mascot are pairs of target.dat and decoy.dat files. Paired dat files with corresponding mzXML files were used as input for Percolator (version 3.02) and SQUA-N (Stable isotope-based Quantitation, Version 2.0) to obtain both identification and quantification results. The process of data processing and graph generation is shown in the figure: Supplemental Table S2 contains all the ambiguous, redundant, non-repeatable and label dependent peptides of FDR < 0.01 from MKK2-ID VS. MKK1-ID in E2 in three biological replicates. Supplemental Table S3a contains unambiguous, redundant, non-repeatable and label-dependent biotinylpeptides from two sets of experiments including E1 and E2. Supplemental Table S3b contains unambiguous, non-redundant, repeatable and label-independent biotinylpeptides from two sets of experiments including E1 and E2. Supplementary Table S3c contains all the biotinylproteins from two sets of experiments. Supplemental Table S3d contains all biotinylproteins with biotin occupancy from two sets of experiments. Supplemental Table S5c contains total peptides of MKK2-ID VS. MKK1-ID of MS1-based method in E2. Supplemental Table S5d contains MS1-based quantification results of MKK2-ID VS. MKK1-ID in E2. Supplemental Table S5e contains the summary of quantification results from Supplemental Table S4c, e and Supplemental Table S5b, d. Supplemental Table S6a contains module analysis results of the interactors of MKK1/2. Supplemental Table S6b contains protein-protein interactions from database and PL experiment between the interactors of MKK1/2. Supplemental Table S7 contains the reanalyzed quantification results of all UPAs from 4 biological replicates of 40s touch data. Supplemental Table S8 contains the identified phosphopeptides from MKK1/2-ID labeled proteins and predicted connection between kinases and their substrates.

Supplemental Figure S1G was generated using Supplemental Table S1. Supplemental Figure S2F was generated using Supplemental Table S2. Supplemental Figure S9A-C was generated using Supplemental Table S2, Supplemental Table S3a and Supplemental Table S3b. Supplemental Figure S9D-F and Figure 1E-F were generated using Supplemental Table S3b. Figure 2A-D, Figure 2H-M, Supplemental Figure S10A-R and Supplemental Figure S11-13 were generated using Supplemental Table S3c. Figure 2E-G were generated using Supplemental Table S3d. Figure 3A, C and Figure 3B, C were generated using Supplemental Table S4c and S4e, respectively. Figure 4B was generated using Supplemental Table S5d. Figure 3D-F and Supplemental Figure S15A were generated using Supplemental Table S5e. Figure 3G-J and Supplemental Figure S14, A and B were generated using Supplemental Table S3d and Supplemental Table S5e. Figure 4H was generated using Supplemental Table S3c and Supplemental Table S6. Figure 5A was generated using Supplemental Table S7a and Supplemental Table S7b. Supplemental Figure S17 was generated using Supplemental Table S8 and Supplemental Table S9. Figure 5B was generated using Supplemental Table S9. Figure 8 were generated using Supplemental Table S5 and Supplemental Table S8. Supplemental Figure S3, Supplemental Figure S18, A-F, Figure 6C, and Supplemental Figure S21, A and B were generated using Supplemental Table S10.

It is related to Figure 1-6 and 8, Supplemental Figure S1, S2, S9-S20, and Supplemental Table S2-S10.

Supplemental FIG. S9

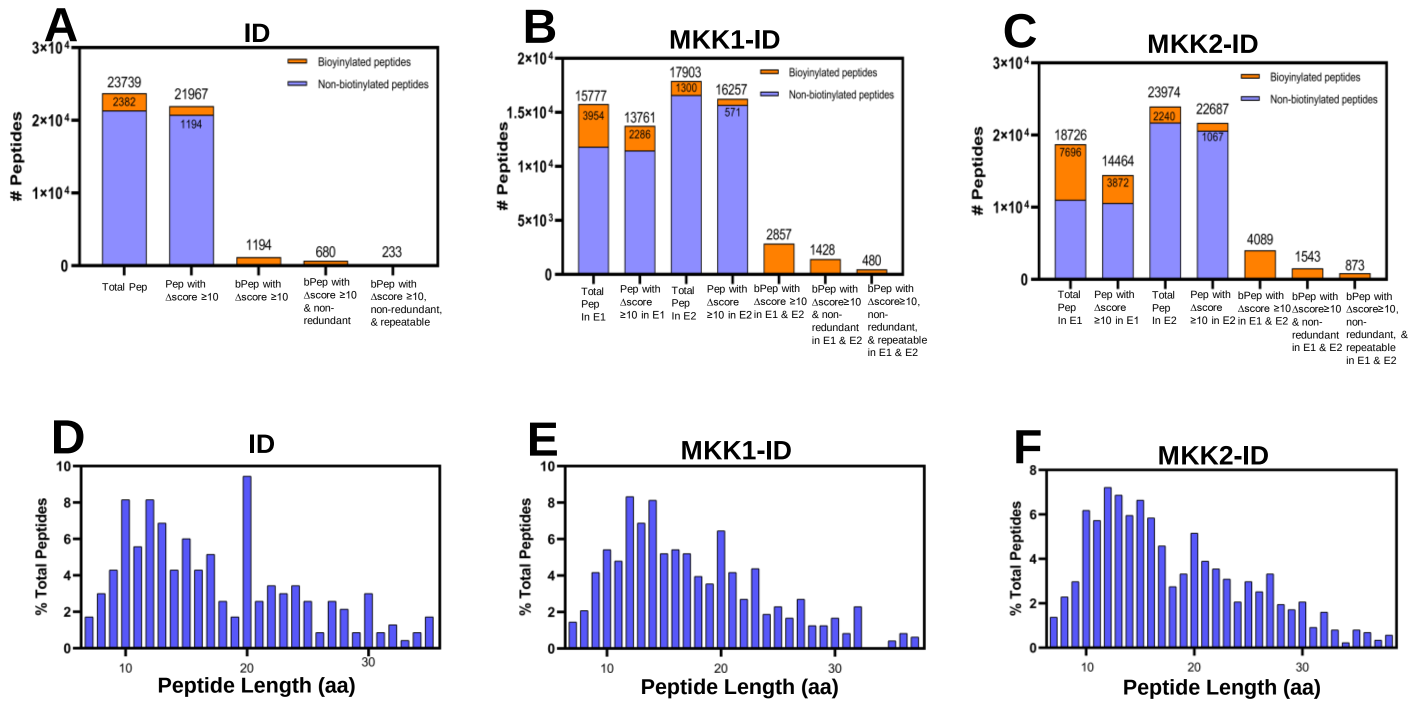

Supplemental FIG. S9. Analysis of identified biotinylpeptides from mass spectrometry. *A*, the bar represents the number of ambiguous, redundant and non-repeatable total peptides (23739) of a FDR <0.01 including 2382 biotinylpeptides, the number (21967) of unambiguous, redundant and non-repeatable total peptides of both FDR <0.01 and delta score ≥10 including 1194 biotinylpeptides, the total number (1194) of unambiguous, redundant and non-repeatable biotinylpeptides of both FDR <0.01 and delta score ≥10, the number (680) of unambiguous, non-redundant, and non-repeatable biotinylpeptides of FDR <0.01 and delta score ≥10 and the number (233) of unambiguous, non-redundant, and repeatable biotinylpeptides of both FDR < 0.01 and delta score ≥ 10 in ID line, respectively. This last group of biotinylpeptides is used for all the whole rest ID biotinylated proteomic analysis (supplemental Table S1*a-f* and *s*upplemental Table S3*a-b*). *B*, the bar represents the number of ambiguous, redundant and non-repeatable total peptides (15777) of a FDR <0.01 including 3954 biotinylpeptides in *MKK1-ID* line in Experiment 1 (E1), the number (13761) of unambiguous, redundant and non-repeatable total peptides of both FDR <0.01 and delta score ≥10 including 2286 biotinylpeptides in *MKK1-ID* line in E1, the number of ambiguous, redundant and non-repeatable total peptides (17903) of a FDR <0.01 including 1300 biotinylpeptides in *MKK1-ID* line in Experiment 2 (E2), the number (16257) of unambiguous, redundant and non-repeatable total peptides of both FDR <0.01 and delta score ≥10 including 571 biotinylpeptides in *MKK1-ID* line in E2, the total number (2857) of unambiguous, redundant and non-repeatable biotinylpeptides of both FDR <0.01 and delta score ≥10 in *MKK1-ID* line in both experiments, the number (1428) of unambiguous, non-redundant, and non-repeatable biotinylpeptides of FDR <0.01 and delta score ≥10 in *MKK1-ID* line in both experiments and the number (480) of unambiguous, non-redundant, and repeatable biotinylpeptides of both FDR < 0.01 and delta score ≥ 10 in *MKK1-ID* line in both experiments, respectively. This last group of biotinylpeptides is used for all the whole rest MKK1-ID biotinylated proteomic analysis (supplemental Table S1*g-l*, supplemental Table S2, and supplemental Table S3*a*,*b*). *C*, the bar represents the number of ambiguous, redundant and non-repeatable total peptides (18726) of a FDR <0.01 including 7696 biotinylpeptides in *MKK2-ID* line in E1, the number (14464) of unambiguous, redundant and non-repeatable total peptides of both FDR <0.01 and delta score ≥10 including 3872 biotinylpeptides in *MKK2-ID* line in E1, the number of ambiguous, redundant and non-repeatable total peptides (23974) of a FDR <0.01 including 2240 biotinylpeptides in *MKK2-ID* line in E2, the number (22687) of unambiguous, redundant and non-repeatable total peptides of both FDR <0.01 and delta score ≥10 including 1067 biotinylpeptides in *MKK2-ID* line in E2, the total number (4089) of unambiguous, redundant and non-repeatable biotinylpeptides of both FDR <0.01 and delta score ≥10 in *MKK2-ID* line in both experiments, the number (1543) of unambiguous, non-redundant, and non-repeatable biotinylpeptides of FDR <0.01 and delta score ≥10 in *MKK2-ID* line in both experiments and the number (873) of unambiguous, non-redundant, and repeatable biotinylpeptides of both FDR < 0.01 and delta score ≥ 10 in *MKK2-ID* line in both experiments, respectively. This last group of biotinylpeptides is used for all the whole rest MKK2-ID biotinylated proteomic analysis (supplemental Table S1*m-r*, supplemental Table S2, and supplemental Table S3*a*,*b*). *D*, *E*, *F*, the distribution of the identified ID, MKK1-ID or MKK2-ID biotinylpeptides over their primary MS identified lengths, respectively (supplemental Table S3*b*). It is related to Figure 1.

Supplemental FIG. S10

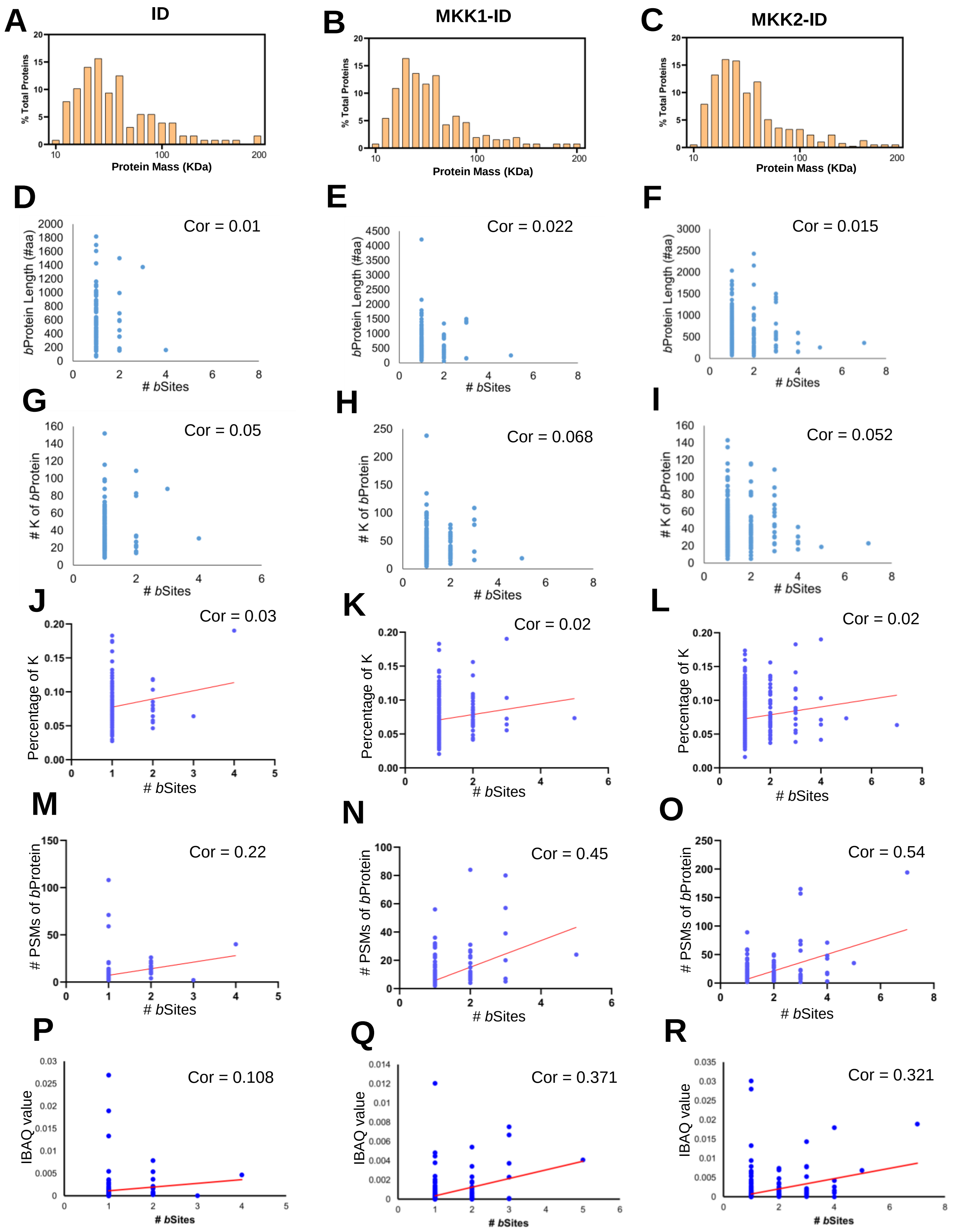

Supplemental FIG. S10. Computational analysis of LC-MS/MS data of biotinylproteins. *A*, *B* and *C*, the distribution of the molecular weight of leading biotinylproteins from *ID*, *MKK1-ID* or *MKK2-ID* plants, respectively. *D*, *E* and *F*, the correlation in between the primary amino acid sequence length and the specific number of biotinylation sites of a biotinylprotein from *ID*, *MKK1-ID* and *MKK2-ID* plants, respectively. The Cor represents the Pearson correlation number. *G*, *H* and *I*, the correlation in between the number of amino acid (K, lysine) with a specific number of biotinylation sites of biotinylproteins. The Cor represents the Pearson correlation number from *ID*, *MKK1-ID* and *MKK2-ID* plants, respectively. *J*, *K* and *L*, the correlation in between percentage of the amino acid (K, lysine) with a specific number of biotinylation sites of biotinylproteins. The Cor represents the Pearson correlation number from *ID*, *MKK1-ID* and *MKK2-ID* plants, respectively. *M*, *N* and *O*, distribution of PSMs of biotinylproteins over the number of biotinylation sites on a biotinylated protein. *P*, *Q* and *R*, distribution of IBAQ value of the biotinylproteins over the number of biotinylation sites on the biotinylated protein. All results were from Supplemental Table S3*c*. It is related to Figure 2.

Supplemental FIG. S11

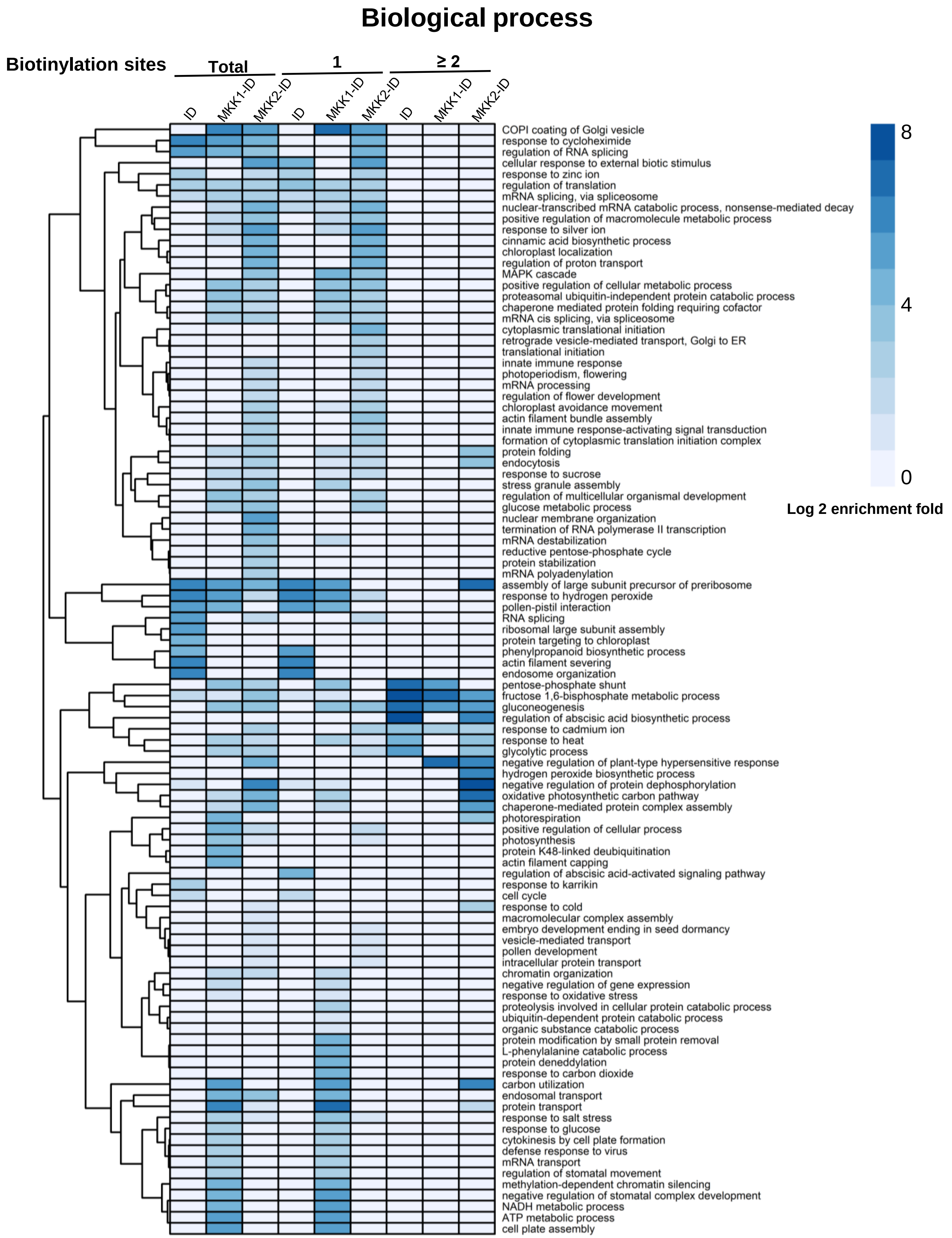

Supplemental FIG. S11. Biological process enrichment analysis of Arabidopsis ortholog of total biotinylproteins, and biotinylproteins with 1 biotinylation site and equal to or more than 2 biotinylation sites listed in Supplemental Table S3*c*. The biotinylproteins were labeled by ID, MKK1-ID and MKK2-ID, respectively. The blue color palette in each cell represents the logarithm of the fold enrichment (log2). It is related to Figure 2.

Supplemental FIG. S12

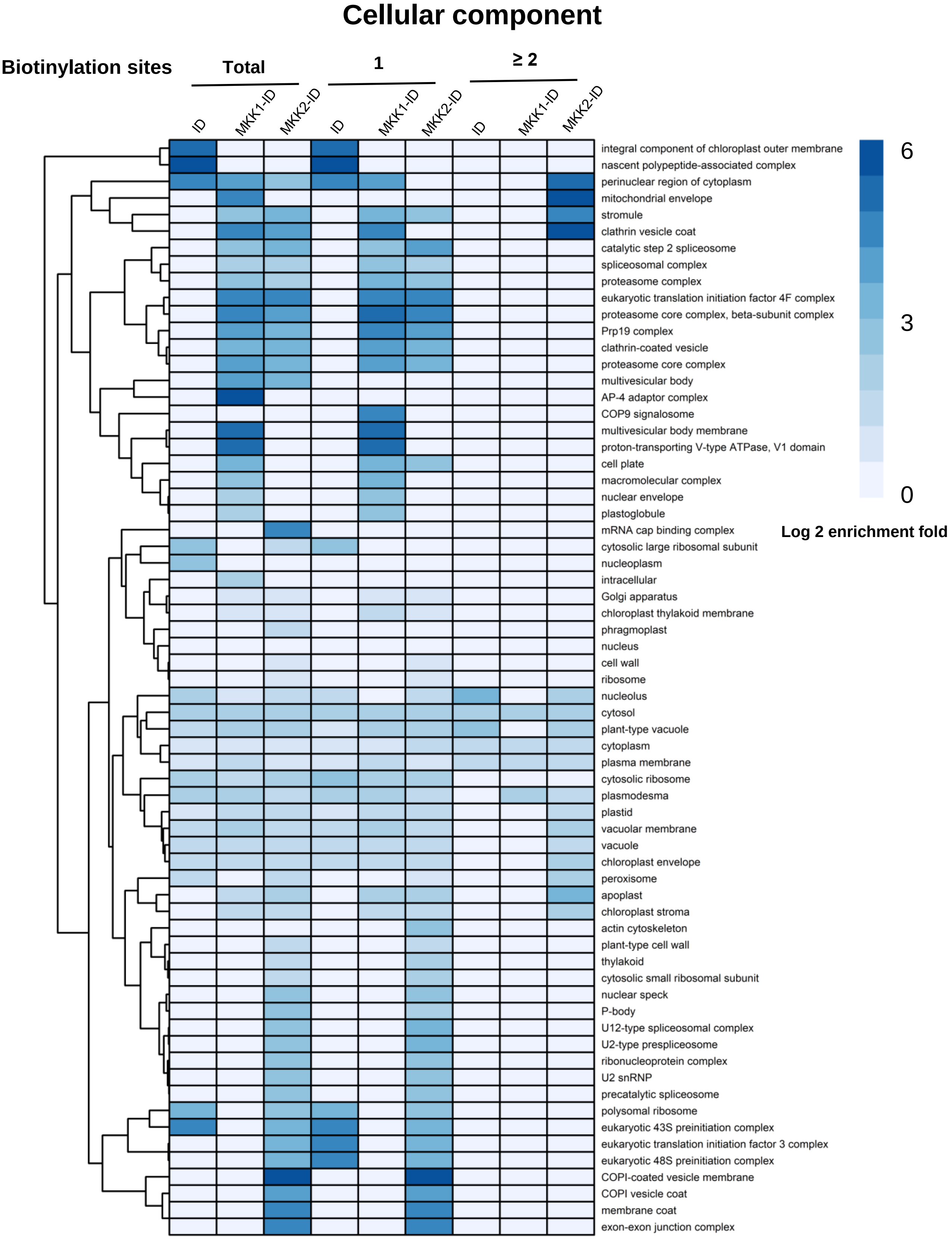

Supplemental FIG. S12. Cellular component enrichment analysis of Arabidopsis ortholog of total biotinylproteins, and biotinylproteins with 1 biotinylation site and equal to or more than 2 biotinylation sites listed in Supplemental Table S3*c*. The biotinylproteins were labeled by ID, MKK1-ID and MKK2-ID, respectively. The blue color palette in each cell represents the logarithm of the fold enrichment (log2). It is related to Figure 2.

Supplemental FIG. S13

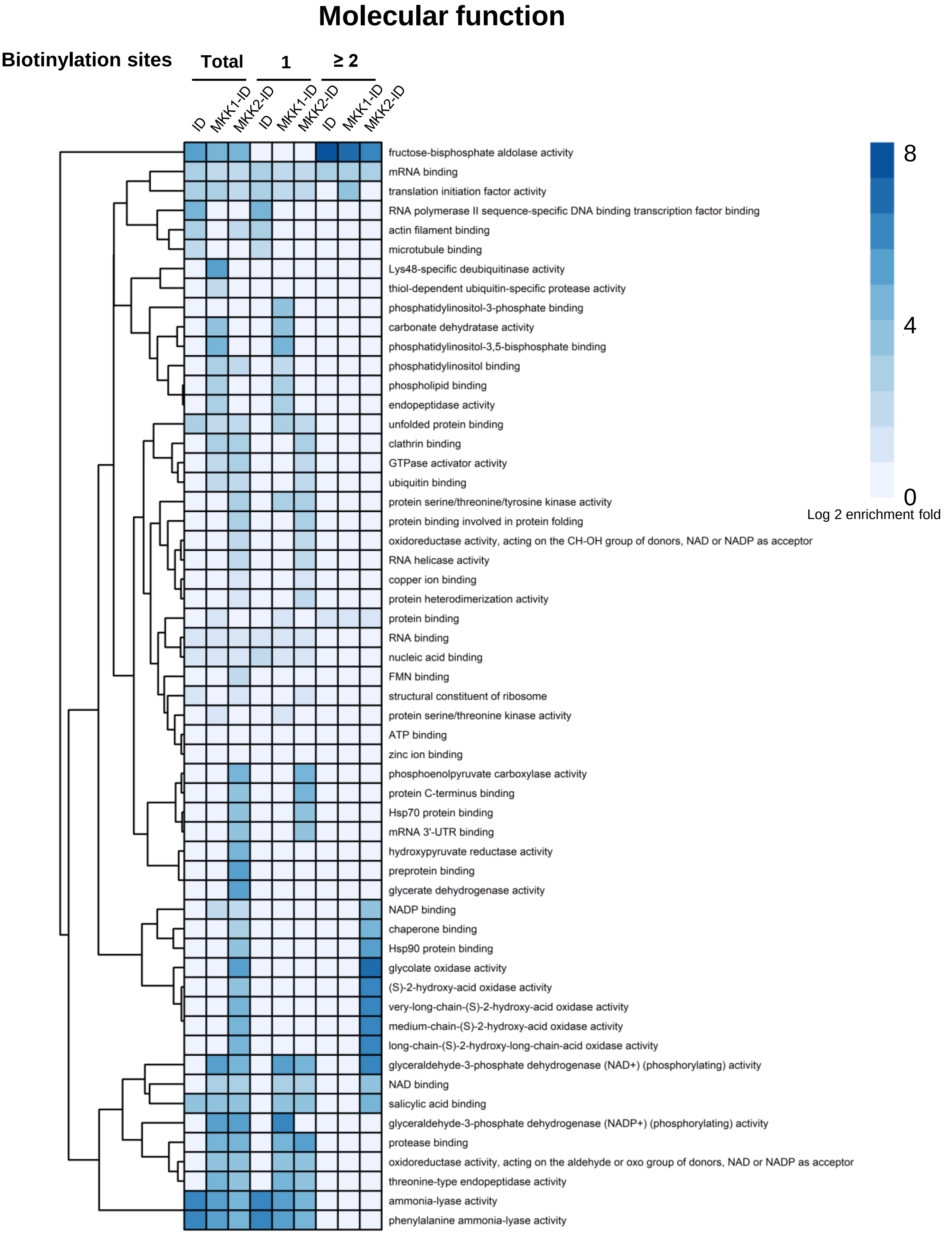

Supplemental FIG. S13. Molecular function enrichment analysis of Arabidopsis ortholog of total biotinylproteins, and biotinylproteins with 1 biotinylation site and equal to or more than 2 biotinylation sites listed in Supplemental Table S3*c*. The biotinylproteins were labeled by ID, MKK1-ID and MKK2-ID, respectively. The blue color palette in each cell represents the logarithm of the fold enrichment (log2). It is related to Figure 2.

Supplemental FIG. S14

A

```
WIRK1 MDEEATSWIRRAKFSQTVSYRLNSSKLASLPFMINQDKFSGLKAIPQRSSSSSSASSSDP  60
RAF43 MDGEVTSWIRRANFSHTVCYRMITPSLESMPFTVNQEKSSGLKRMTLSSPSDLK------  54
      ** *.*******:**:**.**: : .* *:** :**:* **** :    * *. .

WIRK1 KLVSSNSQTTGDTSSLEAADVYVVDSEIQTNPVTHKQRSVSPSPQMAVPDVFKEARSERK  120
RAF43 -SLVGICQSTQQLTGL--SCGSVVGTEVQRNPVTNKKRSVSPLPHMALSDAFIEAKSDIK  111
        : . .*:* : :.*   :    **.:*:* ****:*:***** *:**: *.* **:*: *

WIRK1 RFSTPHPRRVESEKGMKPKLSHK-NSFDKRSFNLR--SPSGPIRDLGTLRIQERVKSKKD  177
RAF43 RFSTPHPRRVEPEKGMKAKSSSRKDSSEKKSVNLRSLSHSGPIRDLSTQKVKERGKSKID  171
      *********** ***** * * : :* :*:*.***  * *******.* :::** *** *

WIRK1 TGWSKLFDNTGRRVSAVEASEEFRVDMSKLFFGLKFAHGLYSRLYHGKYEDKAVAVKLIT  237
RAF43 KKSSKSVDYRGSKVSSAGVLEECLIDVSKLSYGDRFAHGKYSQIYHGEYEGKAVALKIIT  231
      .  ** .*  * :**:. . **  :*:*** :* :**** **::***:**.****:*:**

WIRK1 VPDDDDNGCLGARLEKQFTKEVTLLSRLTHPNVIKFVGAYKDPPVYCVLTQYLPEGSLRS  297
RAF43 APEDSDDIFLGARLEKEFIVEATLLSRLSHPNVVKFVGVNT---GNCIITEYVPRGSLRS  288
      .*:*.*:  *******:*  *.******:****:****. .     *::*:*:*.*****

WIRK1 FLHKPENRSLPLKKLIEFAIDIARGMEYIHSRRIIHRDLKPENVLIDEEFHLKIADFGIA  357
RAF43 YLHKLEQKSLPLEQLIDFGLDIAKGMEYIHSREIVHQDLKPENVLIDNDFHLKIADFGIA  348
      :*** *::****::**:*.:***:********.*:*:**********::***********

WIRK1 CEEEYCDMLADDPGTYRWMAPEMIKRKPHGRKADVYSFGLVLWEMVAGAIPYEDMNP-IQ  416
RAF43 CEEEYCDVLGDNIGTYRWMAPEVLKRIPHGRKCDVYSFGLLLWEMVAGALPYEEMKFAEQ  408
      *******:*.*: *********::** *****.*******:********:***:*:   *

WIRK1 AAFAVVHKNIRPAIPGDCPVAMKALIEQCWSVAPDKRPEFWQIVKVLEQFAISLEREGNL  476
RAF43 IAYAVIYKKIRPVIPTDCPAAMKELIERCWSSQTDKRPEFWQIVKVLEHFKKSLTSEGKL  468
       *:**::*:***.** ***.*** ***:***   **************:*  **  **:*

WIRK1 NLSSSKICKDPRKGLKHWIQKLGPVHAGGGGGSSSSGLGGSALPKPKFA           525
RAF43 NLLPSQICPELKKCPKFWIHIFGSFHHHSSGGGSSS--NNSALPKPKFA           515
      **  *:** : :*  *.**: :* .*  ..**.***  ..*********
```

B

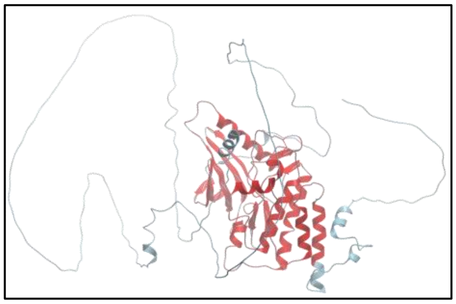

C

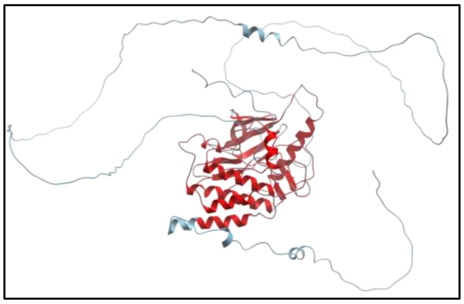

D

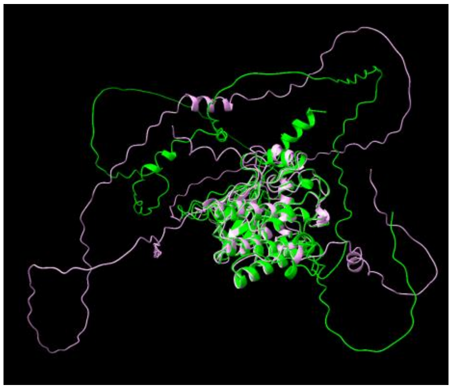

Supplemental FIG. S14. Homolog of WIRK1. *A*, the alignment results between WIRK1 and RAF43. By compositional matrix adjustment method, 59% of the amino acid sequences of RAF43 are identical, and 72% of the sequences are positive. *B and C*, the predicted 3D structures of the WIRK1 and RAF43. Red color represents the kinase domain of the kinase. *D*, the structural superposition of WIRK1 and RAF43 by UCSF ChimeraX . Green and pink colored structure represent WIRK1 and RAF43, respectively. RMSD (The Root-Mean-Square Deviation) between 255 pruned atom pairs is 0.745 angstroms (across all 511 pairs: 36.639).

Supplemental FIG. S15

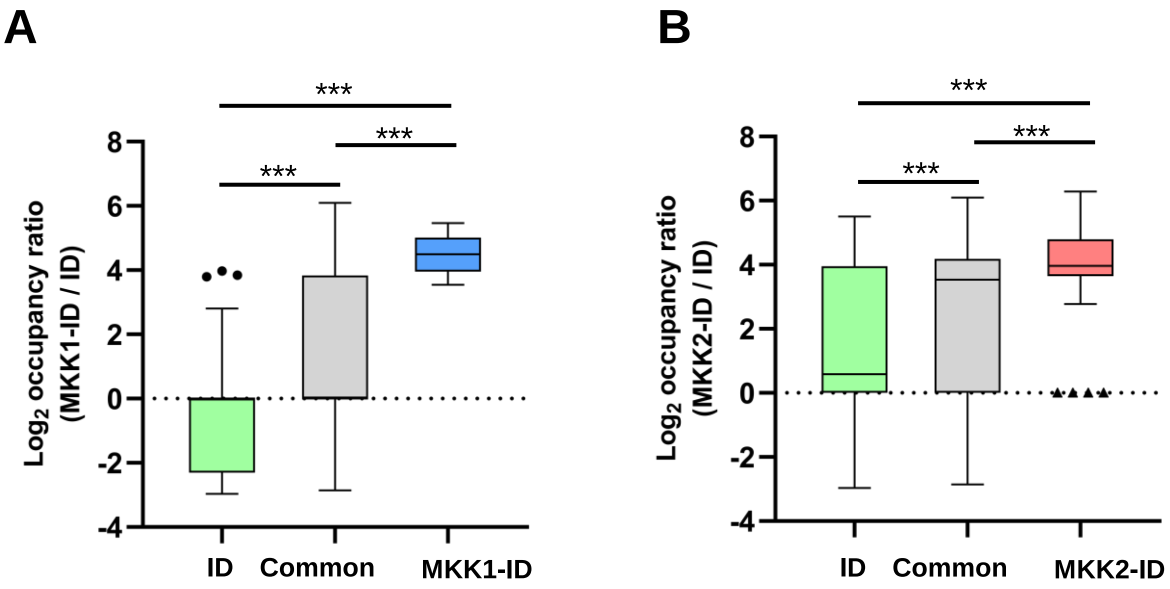

Supplemental FIG. S15. Comparison the BOR among quantitative analysis results. *A* and *B*, statistical analysis of significantly quantified ID associated proteins, MKK1 or MKK2 interactors and other proteins. The box and whisker plots show the distribution of log2-BOR in each group. Unpaired student's t test was applied: ***P < 0.001. (supplemental Table S3*d* and supplemental Table S5*e*). It is related to Figure 3G-3J.

Supplemental FIG. S16

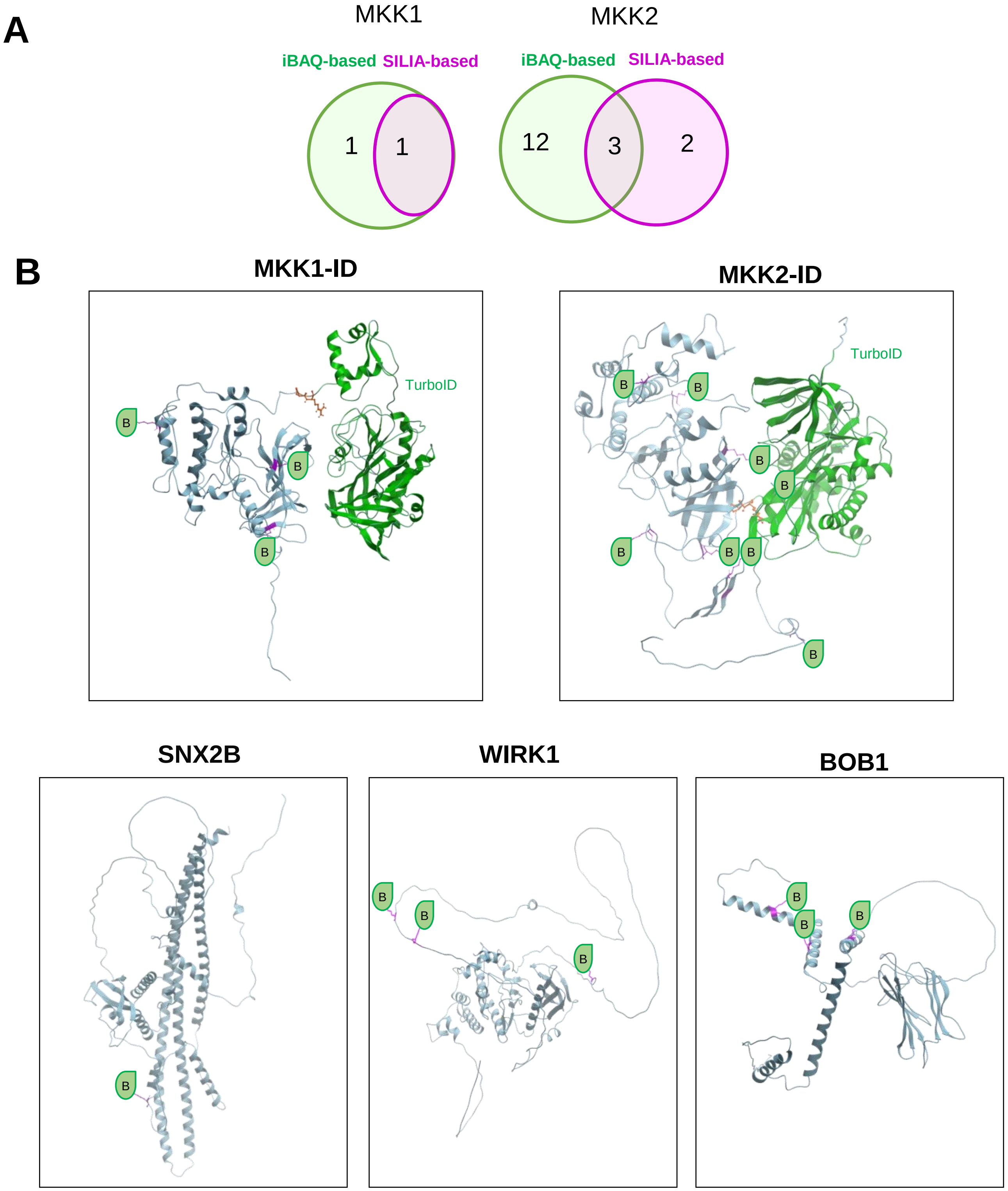

Supplemental FIG. S16. The predicted 3D structures of the MKK1-ID, MKK2-ID and MKK1- and MKK2 interactors. *A*, a venn diagram showing the number of MKK1-specific and MKK2-specific interactors from two quantitation methods. All specific interactors were filtered using the definition of MKK1 and MKK2 interactors (supplemental Table S5*e*). *B*, predicted structure of MKK1-ID, MKK2-ID and MKK1- and MKK2 interactors. Biotinylated lysine residues are annotated in purple and linked to biotin shown in the green diagram. The orange amino acids indicate the short linker between TurboID and MKK1 or MKK2. It is related to Figure 4.

Supplemental FIG. S17

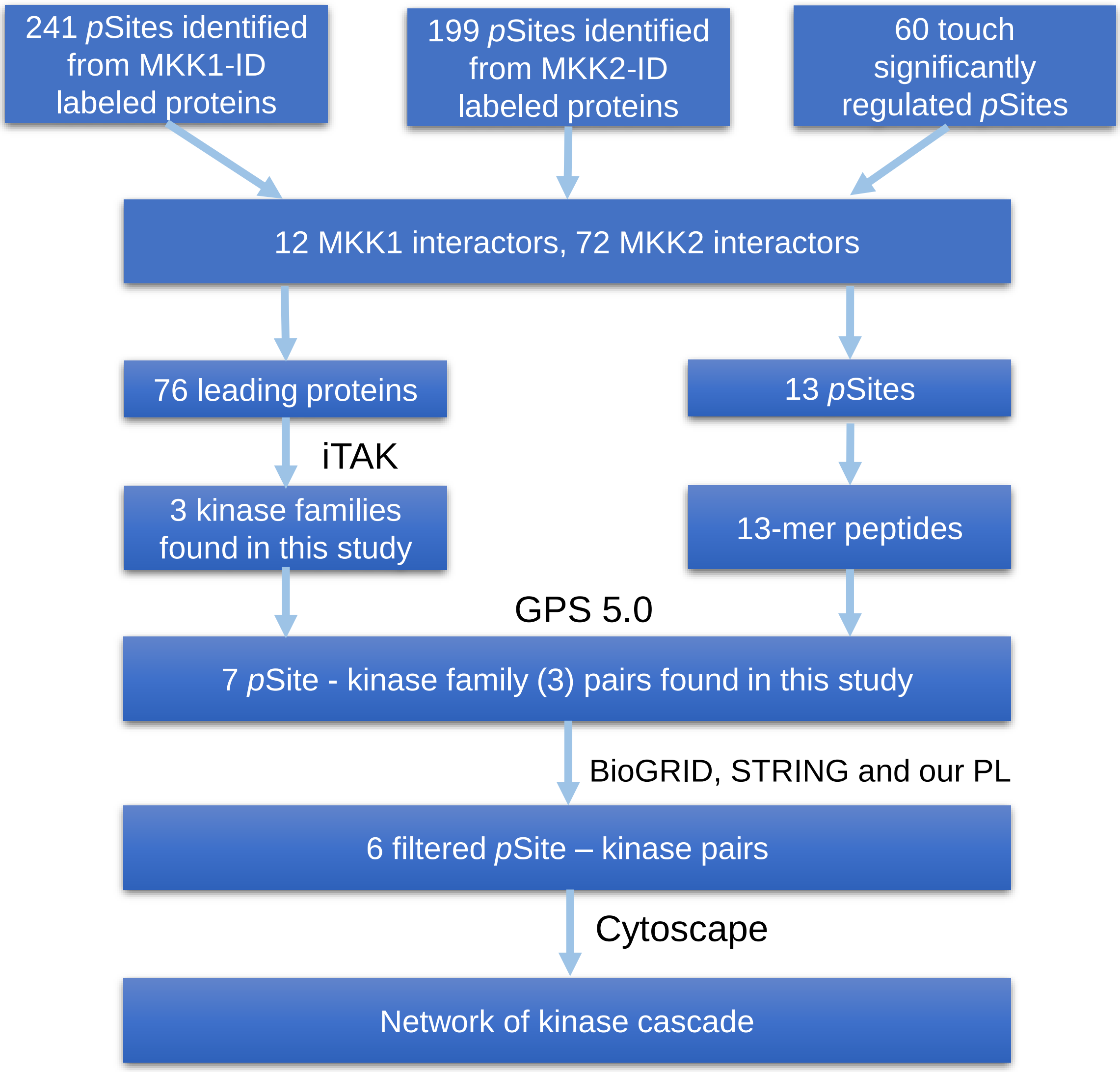

Supplemental FIG. S17. The mapping of the relationship in between kinase family and the kinase docking site motif. 241 *p*Sites identified from MKK1-ID labeled proteins, 199 *p*Sites identified from MKK2-ID labeled proteins and 60 touch significantly regulated *p*Sites were first filtered with 12 MKK1 and 72 MKK2 interactors, and 13 *p*Site were obtained and generated 13-mer peptide sequence though extending to 6 aa long at both ends. 76 leading proteins from 12 MKK1 and 72 MKK2 interactors were classified as 3 kinase families using software iTAK. The possible kinase families that may catalyze these phosphosites are predicated by GPS using the 3-kinase families with 13 13-mer peptide sequences. Consequently, the kinase families grouped by iTAK and those kinase families - phosphosites pairing results predicted by GPS 5.0 were integrated together to map the relationship of phosphosites-dependent kinase families. The total number of phosphosites-kinase family mapping results were further filtered with the protein-protein interaction data predicted either by STRING, BioGrid or our PL data. Finally, 6 phosphosites-kinase pairs were obtained (supplemental Table S8 and supplemental Table S9). It is related to Figure 5*B*.

Supplemental FIG. S18

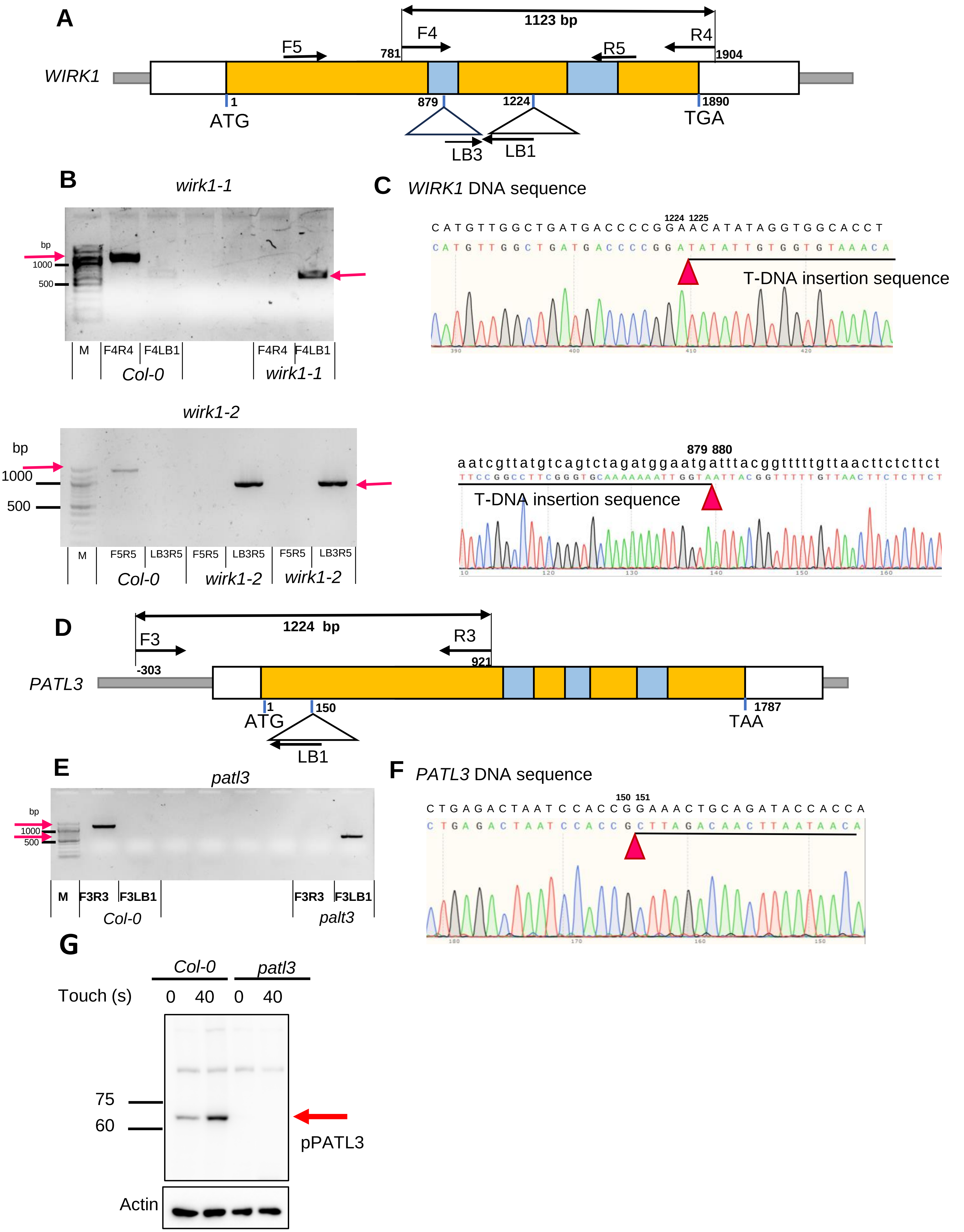

Supplemental FIG. S18. Genotyping of T-DNA insertion mutants of *wirk1-1, wirk1-2* and *patl3*. *A* and *D*, DNAs schematic representation of *WIRK1* and *PATL3*, respectively. Triangles represent T-DNA insertion sites in T-DNA insertional *wirk1-1, wirk1-2* or *patl3* mutants. Primers used to genotype were presented with arrows. White boxes indicated as 5' UTR or 3'UTR. Yellow boxes were exons of WIRK1 or PATL3 including blue intron. ATG and TAA/TGA represent the start and stop codon, respectively. *B*, genotyping of *wirk1-1* and *wirk1-2* T-DNA insertions by PCR. Primer pairs used to verify *wirk1-1* mutant were F4+R4 and LB1+F4. The size of F4+R4 was 1123 bp. Primer pairs used to verify *wirk1-2* mutant were F5+R5 and LB3+R5. The size of F5+R5 was 1433 bp. *C*, T-DNA insertion site verification of *wirk1-1* and *wirk1-2* by DNA sequencing. The insertion site of *wirk1-1* is at 1224 bp of the second exon. The insertion site of *wirk1-2* is at 879 bp of the first intron. *E*, genotyping of *patl3* T-DNA insertions by PCR. Primer pairs used to verify *patl3* mutant were F3+R3 and LB1+F3 The size of F3+R3 was 1214 bp. *F*, T-DNA insertion site verification of *palt3* by DNA sequencing. The insertion site is at 150 bp of the first exon. *G*, negative control of pPATL3 phosphorylation antibody in touch-treated mutant *patl3* compared with Col0. All primers are summarized in Supplemental Table S10. It is related to Figure 6.

Supplemental FIG. S19

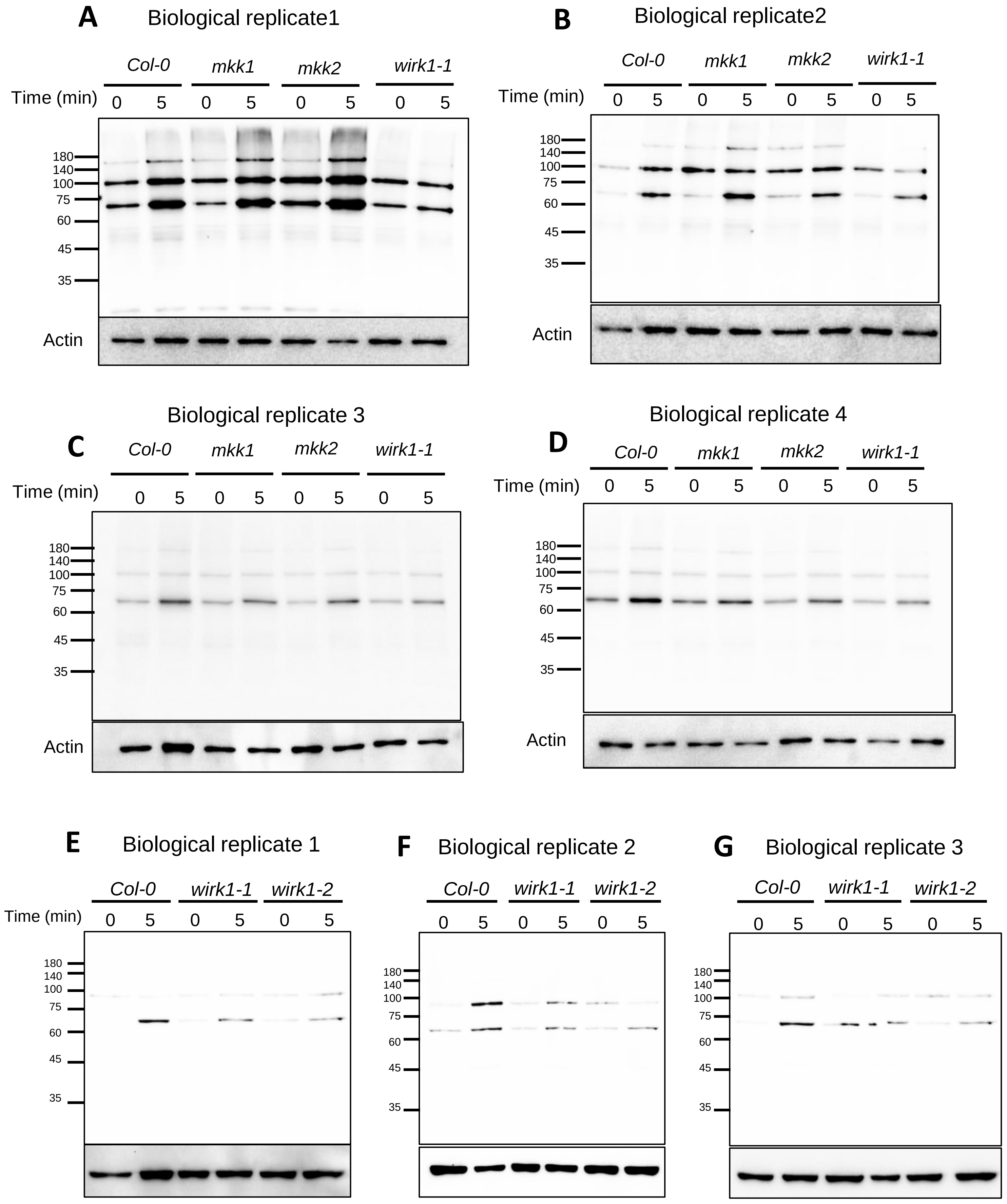

Supplemental FIG. S19. Immunoblot analysis of phosphorylation of PATL3 proteins using phosphosite specific polyclonal antibodies. *A* - *D*, the immunoblots of the whole protein gel showing 5-min wind enhanced phosphorylation of S108 of PATL3 in *Col-0*, *mkk1*, *mkk2* and *wirk1-1* mutant plants for four biological replicates. *E* - *G*, the immunoblots of the whole protein gel showing 5-min wind enhanced phosphorylation of S108 of PATL3 in *Col-0*, *wirk1-1* and *wirk1-2* mutant plants for three biological replicates.

Supplemental FIG. S20

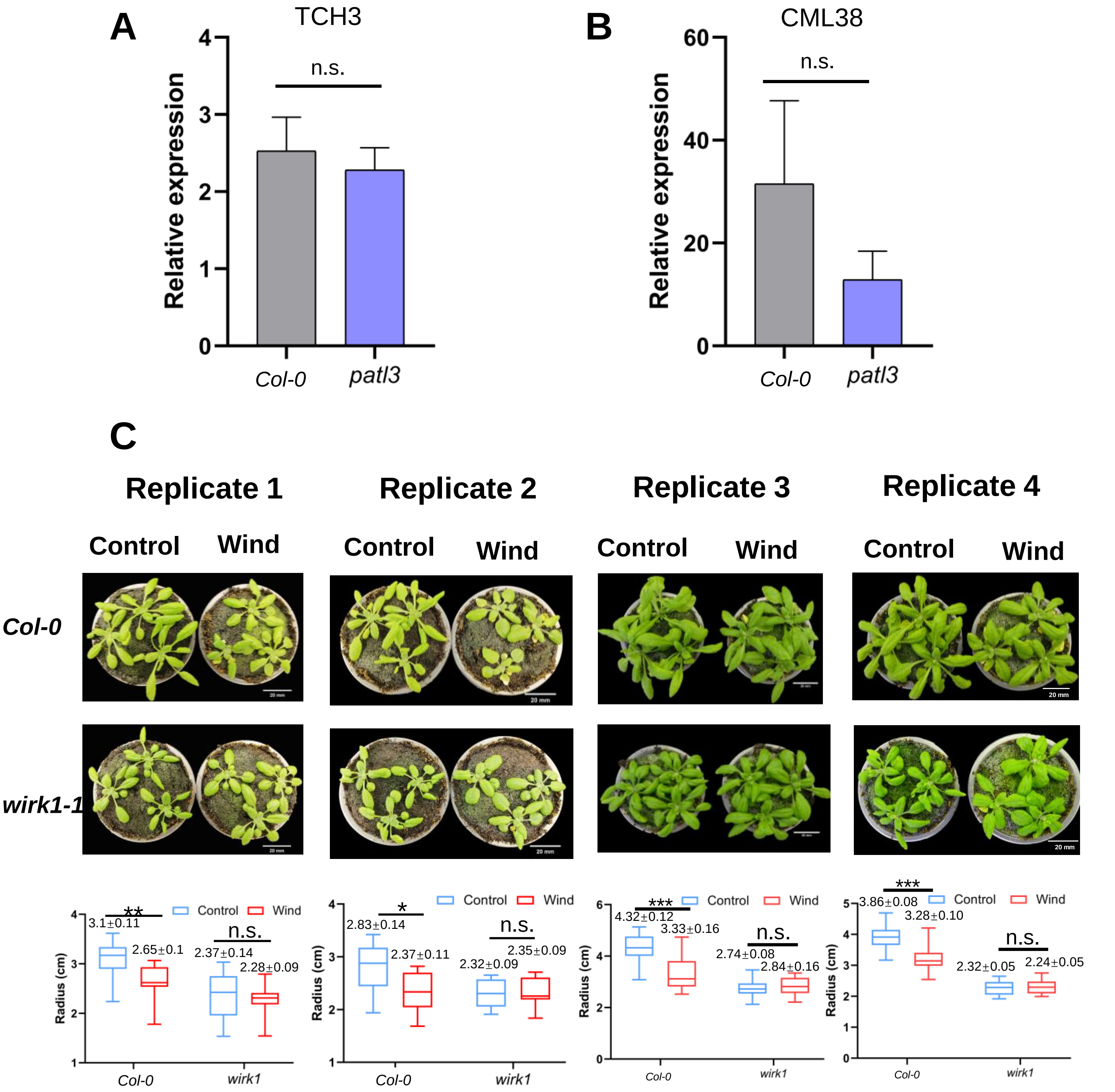

Supplemental FIG. S20. The effects of PATL3 in transcriptional regulation during wind induction and thigmomorphogenesis of *Col-0* and *wirk1-1* mutant plants under wind stimulation. *A* and *B*, TCH3 or CML38 was induced within 30 minutes after 1-min wind treatment in *Col-0* and *patl1* plants. mRNA levels were quantified by RT-qPCR. All primers are summarized in Supplemental Table S10. *C*, four biological replicates for wind response. The charts above are the phenotypes of 10-day-treated plants of *Col-0* and *wirk1-1* mutant plants. The bottom plots are box-and-whisker plots for wind blown and untreated plants. Four biological replicates were performed, and each group contained 9 to 16 plants. It is related to Figure 6 and Supplemental Figure S21.

Supplemental FIG. S21

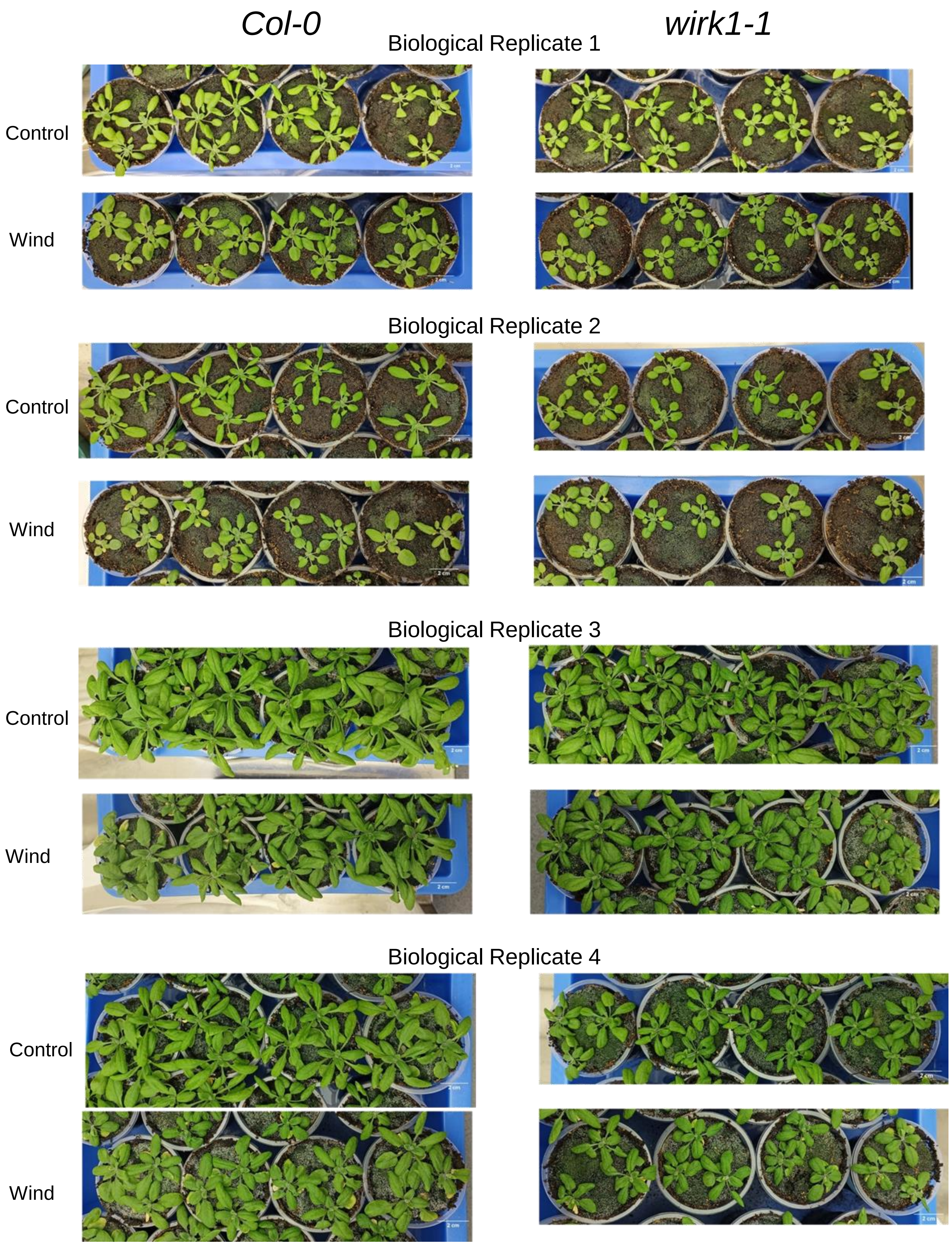

Supplemental FIG. S21. Thigmomorphogenesis of *Col-0* and *wirk1-1* mutant plants under wind stimulation for four biological replicates. It is related to Figure 6*F*.

Supplemental FIG. S22

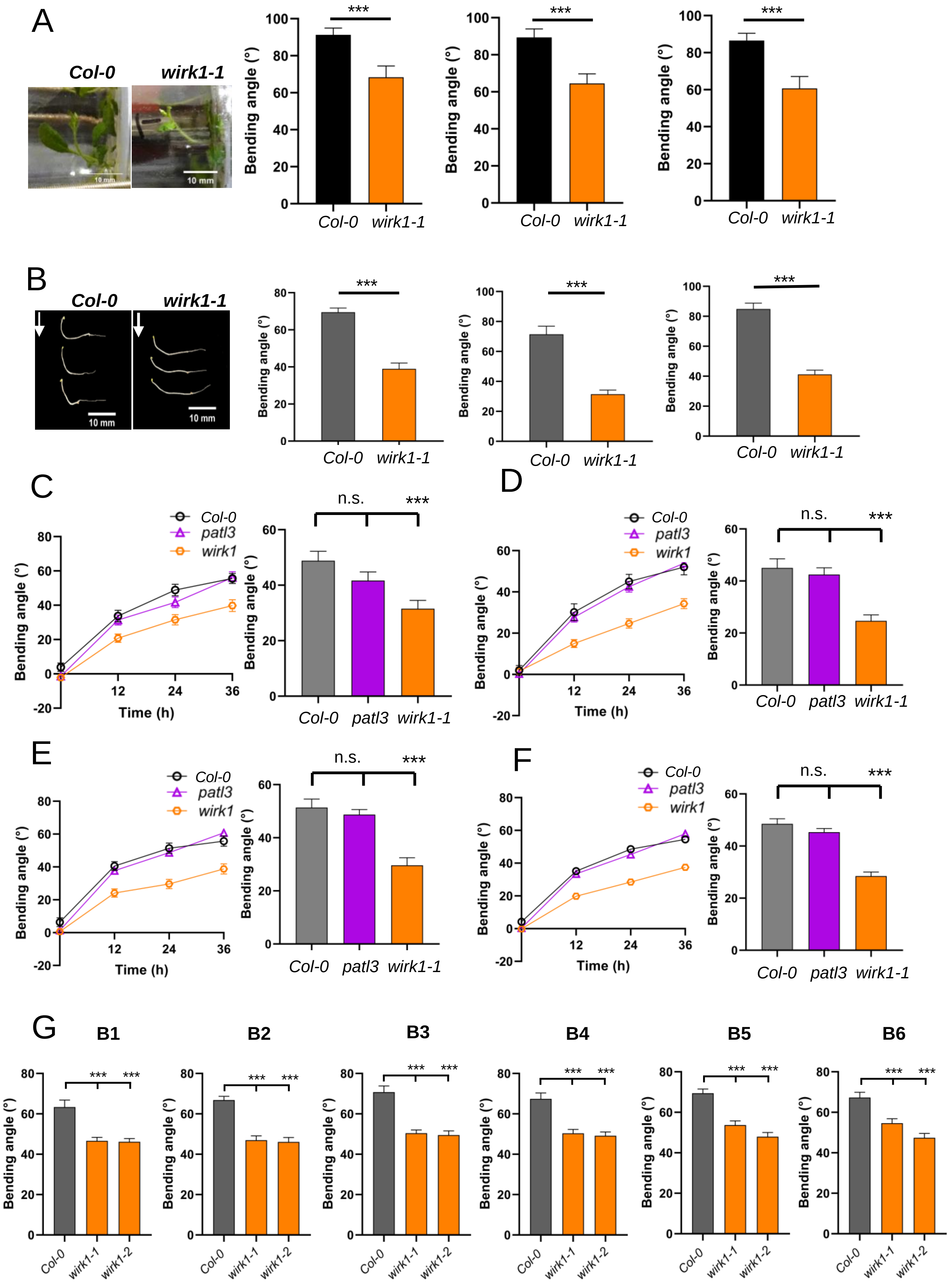

Supplemental FIG. S22. Gravitropic response of *Col-0* and *wirk1-1* and *palt3* mutant plants. *A*, inflorescence stem bending curves of *Col-0* and *wirk1-1* mutant plants for three biological replicates and representative photo. *B*, hypocotyl bending curves of *Col-0* and *wirk1-1* mutant plants for three biological replicates and representative photos. *C* - *F*, hypocotyl bending curves of *Col-0, wirk1-1* and *patl3* plants within 36 h of gravity vector change for four biological replicates (n > 30 per genotype per replicate in each replicate). *G*, hypocotyl bending curves of *Col-0, wirk1-1* and *wirk1-2* plants after gravity vector change for 24 hours in biological replicate experiments (n > 30 per genotype per replicate in each replicate). The statistical test was performed employing student's *t*-test. Significance of $p < 0.05$, $p < 0.01$ and $p < 0.001$ are shown as *, ** and ***, respectively. It is related to Figure 7.

Supplemental FIG. S23

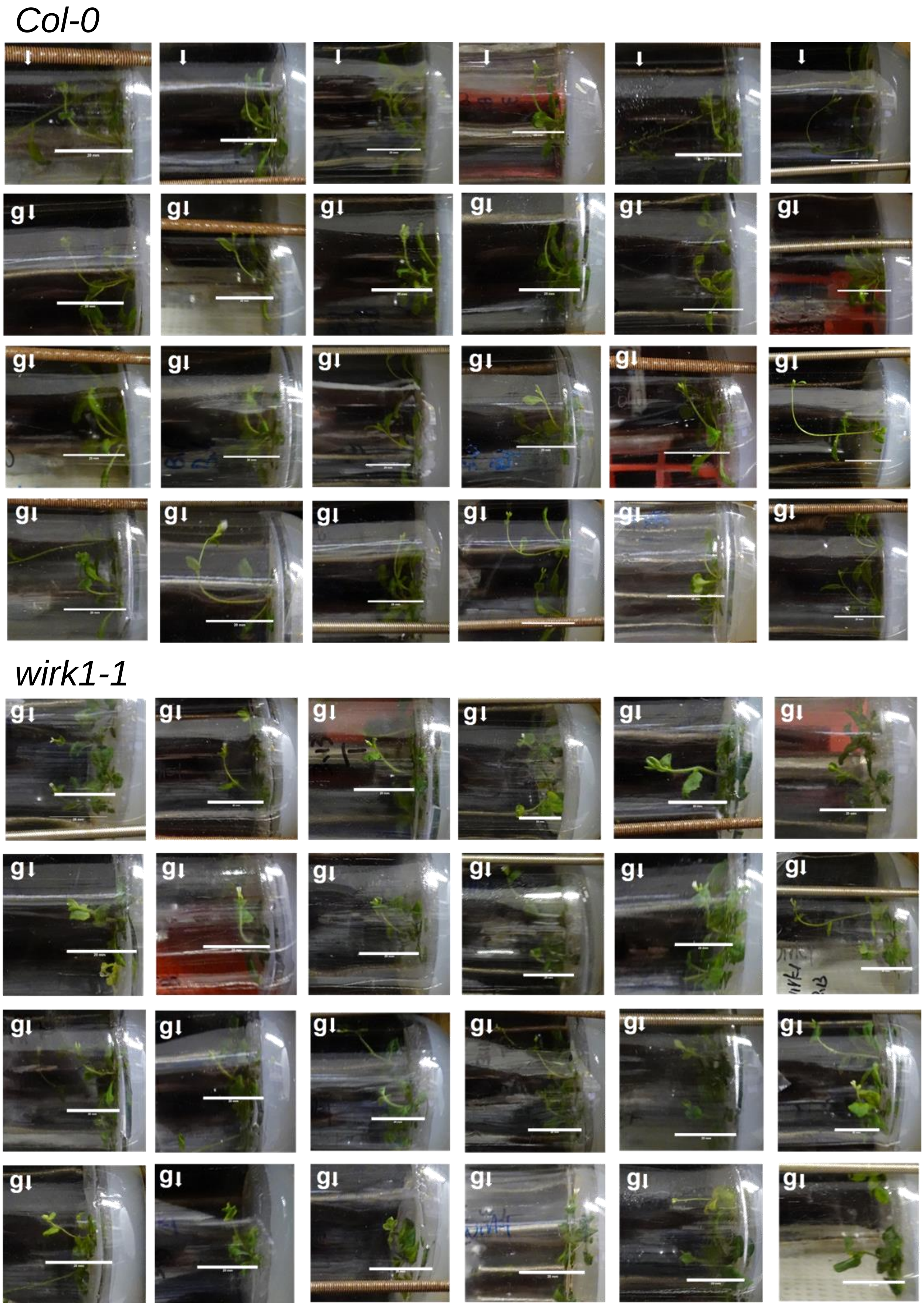

Supplemental FIG. S23. Representative photographs of inflorescence stem bending curves of *Col-0* and *wirk1-1* mutants after changing the gravity vector for 150 min. It is related to Figure 7*A*.

Supplemental FIG. S24

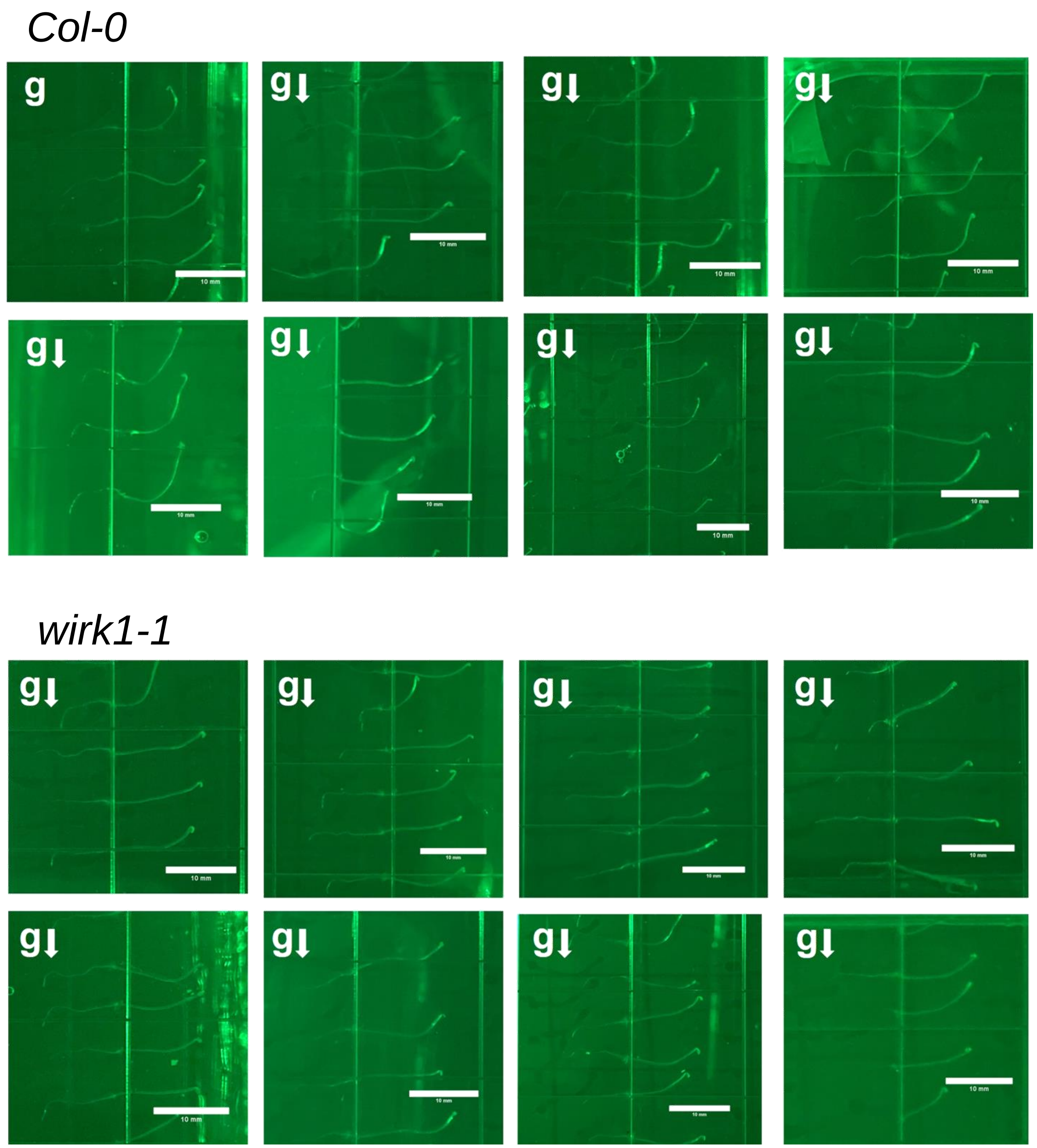

Supplemental FIG. S24. Representative photographs of the hypocotyl bending curves of seedlings of *Col-0* and *wirk1-1* mutants after changing the gravity vector for 36 h. It is related to Figure 7*B*.

Supplemental FIG. S25

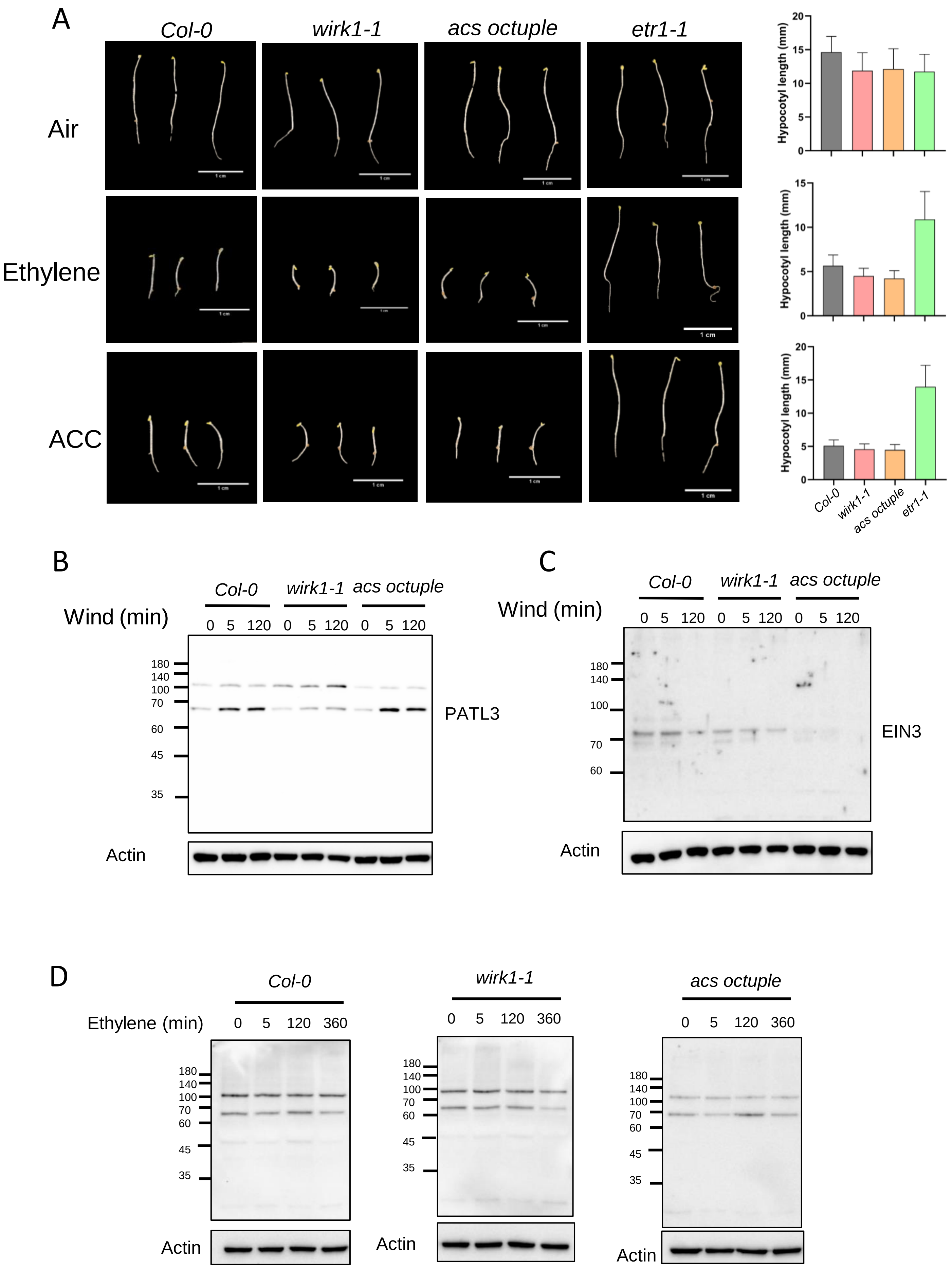

Supplemental FIG. S25. Ethylene effects in wind-induced WIRK1-PATL3 signaling pathway.

A, Hypocotyl phenotype of 4-day-old seedlings grown on MS medium under air conditions, on MS medium under 5 ppm ethylene, and on MS medium with 2.5 μM ACC under air conditions. The representative figures of etiolated seedlings of *Col-0*, *wirk1-1*, *acs octuple* and *etr1-1*mutant plants under the three kinds of treatment were shown in the right panel. The mean hypocotyl lengths of all genotypes from three independent replicates were shown in the right panel. The statistical test was performed employing student's *t*-test. Significance of $p < 0.05$, $p < 0.01$ and $p < 0.001$ are shown as *, ** and ***, respectively. *B*, the immunoblots of the whole protein gel showing 0-, 5- and 120-min wind enhanced phosphorylation of S108 at PATL3 in *Col-0*, *wirk1-1* and *acs octuple* mutant plants. *C*, the immunoblots of the whole protein gel showing 0-, 5- and 120-min wind enhanced expression of EIN3 in *Col-0*, *wirk1-1* and *acs octuple* mutant plants. *D*, the immunoblots of the whole protein gel showing 0-, 5-, 120 and 360-min ethylene enhanced phosphorylation of S108 at PATL3 in *Col-0*, *wirk1-1* and *acs octuple* mutant plants.
